# Biosynthesis of High‐Active Hemoproteins by the Efficient Heme‐Supply *Pichia Pastoris* Chassis

**DOI:** 10.1002/advs.202302826

**Published:** 2023-08-30

**Authors:** Fei Yu, Xinrui Zhao, Jingwen Zhou, Wei Lu, Jianghua Li, Jian Chen, Guocheng Du

**Affiliations:** ^1^ Key Laboratory of Industrial Biotechnology Ministry of Education School of Biotechnology Jiangnan University 1800 Lihu Road Wuxi Jiangsu 214122 China; ^2^ Science Center for Future Foods Jiangnan University 1800 Lihu Road Wuxi Jiangsu 214122 China; ^3^ Jiangsu Province Engineering Research Center of Food Synthetic Biotechnology Jiangnan University 1800 Lihu Road Wuxi Jiangsu 214122 China; ^4^ Engineering Research Center of Ministry of Education on Food Synthetic Biotechnology Jiangnan University 1800 Lihu Road Wuxi Jiangsu 214122 China; ^5^ Dongsheng Biotech Co., Ltd. 91–92 Junmin Road Taixing Jiangsu 225432 China; ^6^ Key Laboratory of Carbohydrate Chemistry and Biotechnology Ministry of Education Jiangnan University 1800 Lihu Road Wuxi Jiangsu 214122 China

**Keywords:** artificial meat, heme‐supply chassis, high‐cell‐density fermentation, highly active hemoproteins, Pichia pastoris, whole‐cell biocatalysts

## Abstract

Microbial synthesis of valuable hemoproteins has become a popular research topic, and *Pichia pastoris* is a versatile platform for the industrial production of recombinant proteins. However, the inadequate supply of heme limits the synthesis of high‐active hemoproteins. Here a strategy for enhancing intracellular heme biosynthesis to improve the titers and functional activities of hemoproteins is reported. After selecting a suitable expressional strategy for globins, the efficient heme‐supply *P. pastoris* chassis is established by removing the spatial segregation during heme biosynthesis, optimizing precursor synthesis, assembling rate‐limiting enzymes using protein scaffolds, and inhibiting heme degradation. This robust chassis produces several highly active hemoproteins, including porcine myoglobin, soy hemoglobin, *Vitreoscilla* hemoglobin, and P450‐BM3, which can be used in the development of artificial meat, high‐cell‐density fermentation, and whole‐cell catalytic synthesis of high‐value‐added compounds. Furthermore, the engineered chassis strain has great potential for producing and applying other hemoproteins with high activities in various fields.

## Graphical abstract

Here, the development of an engineered heme‐supply *P. pastoris* chassis capable of synthesizing high‐active hemoproteins is reported. After refining the expression of globin, the intracellular heme level is improved by removing spatial segregation, optimizing precursor synthesis, and assembling enzymes with protein scaffolds. These metabolic strategies will be generally useful for producing hemoproteins suitable for artificial meat and several fields.

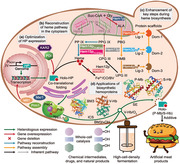

## 1 Introduction

Hemoproteins are heme‐binding proteins, including hemoglobin, myoglobin, cytochrome P450 enzyme, catalase, and peroxidase, which play vital roles in diverse cellular functions.^[^ ^1^, ^2^, ^3^, ^4^ ^]^ Currently, these hemoproteins have been extensively applied in the fields of food,^[^ ^5^ ^]^ medicine,^[^ ^6^ ^]^ high‐cell‐density fermentation,^[^ ^7^ ^]^ and biocatalysts.^[^ ^8^ ^]^ Among them, porcine myoglobin (P‐Mb) is closely related to the red color and metallic taste of meat,^[^ ^9^ ^]^ and soy hemoglobin (S‐Hb) has been employed as a color additive in producing artificial meat.^[^ ^10^ ^]^ In addition, *Vitreoscilla* hemoglobin (V‐Hb), with strong oxygen transfer capacity, is a powerful tool in metabolic engineering to enhance cell growth and product synthesis.^[^ ^11^, ^12^ ^]^ Furthermore, the cytochrome P450‐BM3 monooxygenase derived from *Bacillus megaterium* can catalyze regio‐ and stereo‐selective hydroxylation reactions, making it an efficient biocatalyst to synthesize various valuable compounds such as drugs (steroidal C7β alcohols),^[^ ^13^ ^]^ the natural product (phenol),^[^ ^14^ ^]^ and chemical intermediates (cyclooctanone and hydroquinone).^[^ ^15^, ^16^ ^]^

With the growing demand for hemoproteins, including P‐Mb, S‐Hb, V‐Hb, and P450‐BM3 in the food industry, high‐cell‐density fermentation, and the synthesis of high‐value‐added compounds, it is necessary to develop a microbial platform that can efficiently synthesize these hemoproteins with high bioactivity. Although P‐Mb and S‐Hb have been synthesized in *Escherichia coli*,^[^ ^17^, ^18^ ^]^ this host is unsuitable for food production because of the high risk from endotoxins. Alternatively, our recent research synthesized various Hb and Mb in *Saccharomyces cerevisiae*, including S‐Hb, clover‐Hb, bovine‐Hb/Mb, and P‐Hb/Mb.^[^ ^19^ ^]^ However, the expressional level of these Hb and Mb in *S. cerevisiae* is much lower than in *Pichia pastoris* (also known as *Komagataella phaffii*).^[^ ^20^ ^]^ In addition, the inefficient secretory expression capacity of *S. cerevisiae* increases the cost of purification and limits the industrial‐scale production of hemoproteins. Thus, the USA Food and Drug Association (FDA)‐approved Generally Recognized as Safe (GRAS) strain of *P. pastoris* with a strong expressional and secretory capacity^[^ ^21^, ^22^ ^]^ is a promising candidate for producing hemoproteins.

Currently, S‐Hb and bovine Mb synthesized in *P. pastoris* have been approved by FDA (GRN No. 737 and No. 1001) and applied by Impossible Foods Inc.^[^ ^23^ ^]^ and Motif FoodWorks Inc. to develop the popular artificial meat products “Impossible Burgers” and “HEMAMI”, respectively. In addition, *P. pastoris* has an advantage over *E. coli* and *S. cerevisiae* in high‐cell‐density fermentation because it can grow on simple and inexpensive carbon sources to high densities.^[^ ^21^ ^]^ In the case of high‐cell‐density, efficient oxygen transfer is particularly significant. Hence, V‐Hb with powerful oxygen transport capacity was frequently expressed in *P. pastoris* to promote cell growth and product synthesis, including lipase 2,^[^ ^24^ ^]^ β‐mannanase mutant,^[^ ^25^ ^]^ and xylanase.^[^ ^26^ ^]^ As for whole‐cell P450s catalysis, *E. coli* is a commonly utilized host to heterologously express soluble P450s.^[^ ^13^, ^14^, ^15^, ^16^ ^]^ However, *P. pastoris* has good tolerance to organic solvents and more significant potential for producing hydrophobic compounds than *E. coli* and *S. cerevisiae*.^[^ ^27^, ^28^ ^]^ Notably, a previous study found that among four commonly used microbial hosts (*E. coli*, *S. cerevisiae*, *P. pastoris*, and *Yarrowia lipolytica*), *P. pastoris* exhibited the highest catalytic activity and stability for human cytochrome P450 2D6.^[^ ^29^ ^]^

Although three other hemoproteins (P‐Mb, S‐Hb, and V‐Hb) have been expressed successfully in *P. pastoris*,^[^ ^9^, ^20^, ^25^ ^]^ except for P450‐BM3, there are still three bottlenecks that hinder the efficient synthesis of these high‐active hemoproteins. The first is the low expressional level of the globin component in multitudinous hemoproteins. In our previous report, P‐Mb titer could only reach 7.73 mg L^−1^ at shaking‐flask level using the moderate constitutive promoter P*_GAP_*.^[^ ^9^ ^]^ Therefore, attempting other expressional strategies for hemoproteins is necessary, such as using the powerful methanol‐inducible promoter P*_AOX1_*. Recent research also found that the high inducible expression significantly increased the titer of S‐Hb.^[^ ^20^ ^]^ However, the integrated expression of multi‐copy *S‐Hb* genes inhibited cell growth due to the excess metabolic burdens.^[^ ^20^ ^]^ Therefore, the enhanced strength of the P*_AOX1_* promoter by overexpressing its specific activators (Mit1, Mxr1, and Prm1)^[^ ^30^ ^]^ can be considered an alternative approach to reduce the number of integrated heterologous genes. Additionally, the result of SDS‐PAGE showed that the secreted S‐Hb was severely degraded during the process of fed‐batch fermentation (≈40%).^[^ ^20^ ^]^ Thus, the proteases that result in the degradation of hemoproteins should be inhibited. Furthermore, a previous study found that the expression level of V‐Hb was inversely proportional to the ethanol production in *E. coli*.^[^ ^31^ ^]^ This suggests that the expression of V‐Hb must be controlled at a suitable level to facilitate cell growth and the synthesis of products. However, little attention has been paid to this important issue in *P. pastoris*.^[^ ^24^, ^25^, ^26^ ^]^

**Figure 1:**
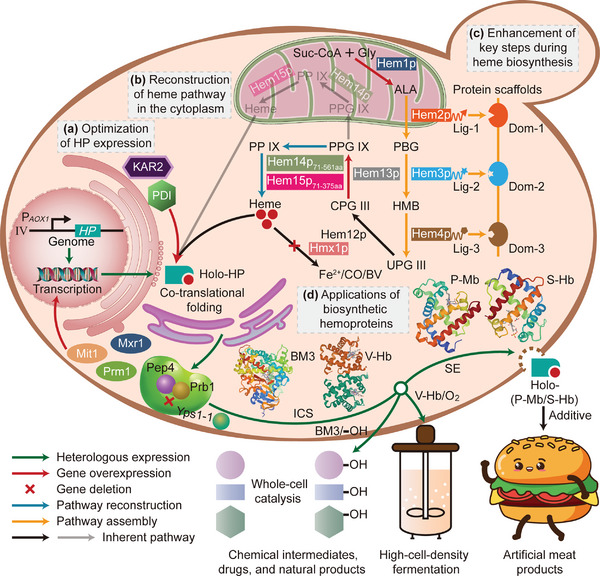
Engineering strategies for efficiently synthesizing highly active hemoproteins through optimizing globin expression and moderately enhancing heme supply. a) Optimization of globin expression by overexpressing P*_AOX1_* transcriptional activators (*Mit1*, *Mxr1*, and *Prm1*) and chaperones (*PDI* and *KAR2*), and knocking out proteases (*pep4*, *prb1*, and *yps1‐1*). b) Reconstruction of the heme biosynthetic pathway in the cytoplasm by co‐expressing MLS‐truncated versions of Hem14p and Hem15p (Hem14p_71‐561aa_ and Hem15p_71‐375aa_). c) Enhancement of key steps during heme biosynthesis by overexpressing *HEM1* and *HEM13*, assembling Hem2p, Hem3p, and Hem4p, and deleting heme oxygenase *HMX1*. Protein scaffolds harboring interaction domains specifically accumulate pathway enzymes tagged with their cognate peptide ligands.^[^ ^39^ ^]^ d) Biochemical properties and potential applications of several hemoproteins synthesized in the efficient heme‐supply chassis of *P. pastoris*. This includes the common characteristic absorption peak and heme‐binding ratio, the specific peroxidase activity of P‐Mb and S‐Hb, the oxygen‐binding property of V‐Hb, and the whole‐cell catalytic efficiency of P450‐BM3. The 3D structure of P‐Mb, S‐Hb, V‐Hb, and P450‐BM3 was obtained from the PDB database (1MWD, 1BIN, 2VHB, and 1FAG, respectively). Abbreviations: HP, hemoprotein; IV, chromosome IV; aa, amino acid; Suc‐CoA, succinyl‐CoA; Gly, glycine; Hem1p, ALA synthase; ALA, 5‐aminolevulinic acid; Hem2p, porphobilinogen synthase; PBG, porphobilinogen; Hem3p, porphobilinogen deaminase; HMB, hydroxymethylbilane; Hem4p, uroporphyrinogen‐III synthase; UPG III, uroporphyrinogen‐III; Hem12p, uroporphyrinogen‐III decarboxylase; CPG III, coproporphyrinogen‐III; Hem13p, coproporphyrinogen‐III oxidase; PPG IX, protoporphyrinogen‐IX; Hem14p, protoporphyrinogen oxidase; PP IX, protoporphyrin‐IX; Hem15p, ferrochelatase; CO, carbon monoxide; BV, biliverdin; Lig, ligand; Dom, domain; ICS, intracellular synthesis; SE, secretory expression.

The second bottleneck is the spatial isolation of heme biosynthesis (**Figure** **1**). Previous studies and some websites (http://www.weizmann.ac.il/molgen/loqate/) reported that the eight heme biosynthetic enzymes (HBS) were distributed between the cytoplasm (Hem2p, Hem3p, Hem4p, Hem12p, and Hem13p) and mitochondria (Hem1p, Hem14p, and Hem15p) in *S. cerevisiae*.^[^ ^19^ ^]^ Heme intermediates must cross the inner and outer mitochondrial membranes several times during heme biosynthesis, reducing the efficiency of heme synthesis and utilization.^[^ ^32^ ^]^ Thus, reconstruction of the heme synthetic pathway in the cytoplasm is vital to efficiently synthesizing both heme and hemoproteins in yeast. However, it has been shown that the localization of intracellular enzymes between *S. cerevisiae* and *P. pastoris* is not entirely consistent.^[^ ^33^ ^]^ Additionally, the bioinformatic tool (TPpred 3.0) can predict the possible mitochondrial localization signal (MLS) of Hem15p in *S. cerevisiae*.^[^ ^19^ ^]^ However, a similar prediction is not feasible for the MLS of Hem15p for *P. pastoris*. Hence, the accurate subcellular localization and MLS of HBS in *P. pastoris* are still needed to be validated for the further metabolic engineering of the heme biosynthetic pathway.

The third bottleneck is the moderate enhancement of the rate‐limiting steps in the heme biosynthetic pathway to meet the demand for synthesizing hemoproteins. In *P. pastoris*, all genes involved in heme biosynthesis had been overexpressed to enhance the production of S‐Hb.^[^ ^20^ ^]^ However, this strategy is inappropriate for improving hemoprotein production because it occupies too much metabolic flux.^[^ ^16^ ^]^ Furthermore, although *S. cerevisiae* could synthesize the highest titer of heme at 53.7 mg L^−1^ by genome‐scale modeling,^[^ ^34^ ^]^ this strategy is also unsuitable for synthesizing hemoproteins because only 4.62 mg L^−1^ heme is needed for 100 mg L^−1^ S‐Hb at the theoretical level and surplus free heme is toxic to cells.^[^ ^16^ ^]^ Therefore, in our recent research, the titer of intracellular heme was increased to improve the production of P‐Mb and S‐Hb through the moderate integration of the *HEM1* gene and the assembly of Hem13p, Hem14p, and Hem15p in *S. cerevisiae*.^[^ ^19^ ^]^ Although these strategies led to the improved production of P‐Mb and S‐Hb, the heme‐binding ratio remains low (20% for P‐Mb and 27% for S‐Hb). The main reason for this effect is that the contribution of other HBS to heme synthesis, including Hem2p, Hem3p, and Hem4p, was not taken into consideration. It has been reported that Hem2p and Hem3p are the rate‐limiting steps during heme biosynthesis in yeast.^[^ ^35^, ^36^ ^]^ In addition, hydroxymethylbilane (HMB) can spontaneously convert to the byproduct uroporphyrinogen I when the activity of Hem4p is limited.^[^ ^37^ ^]^ Therefore, more attention should be paid to eliminating rate‐limiting steps catalyzed by Hem2p, Hem3p, and Hem4p in *P. pastoris*.

Here we report the development of an efficient heme‐supply *P. pastoris* chassis capable of synthesizing high‐active hemoproteins (Figure 1). Initially, the issues of low expressional levels and severe degradation of globin observed in previous studies^[^ ^9^, ^20^ ^]^ were addressed by strengthening the transcription of the P*_AOX1_*‐driven globin gene and knocking out proteases. Next, the low efficiency of heme synthesis and utilization, caused by spatial segregation,^[^ ^32^ ^]^ was overcome by reconstructing the heme biosynthetic pathway in the cytoplasm. Subsequently, the inadequate heme supply resulting from the rate‐limiting steps during heme biosynthesis,^[^ ^19^ ^]^ and the significant accumulation of heme intermediates caused by the overexpression of all HBS,^[^ ^38^ ^]^ were resolved by assembling rate‐limiting enzymes using protein scaffolds. Finally, the titers and functional activities of P‐Mb, S‐Hb, V‐Hb, and P450‐BM3 were significantly increased by combining a suitable expressional platform for globin and with an optimized heme biosynthetic pathway. Thus, the strategies designed in this study are advantageous for producing hemoproteins that can be used in the development of artificial meat, high‐cell‐density fermentation, and the synthesis of high‐value‐added compounds.

## 2 Results

### 2.1 Selecting a Suitable Expressional Platform for the Globin Component

**Figure 2:**
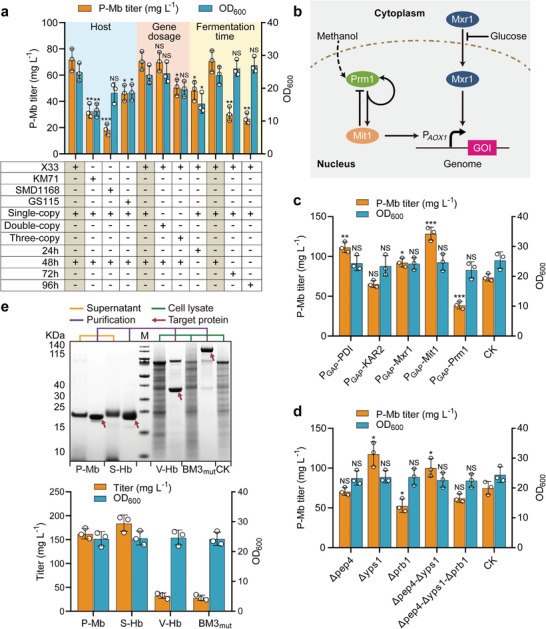
The optimal expressional platform for the globin component of hemoproteins. a) Selecting a suitable expressional system for P‐Mb globin. SDS‐PAGE analysis is presented in Figure S1a (Supporting Information). The three boxes with brown indicate the control groups for optimizing the *P. pastoris* host, gene dosage, and fermentation time, respectively. b) Cascade regulation of Mxr1, Prm1, and Mit1 activates the P*_AOX1_* promoter.^[^ ^30^ ^]^ Glucose inhibits P*_AOX1_* through cytoplasmic Mxr1. Methanol triggers the nuclear translocation of Mxr1, leading to the derepression of P*_AOX1_*. Prm1 responds to methanol and induces its own expression and that of Mit1, which activates the transcription of the P*_AOX1_*‐driven genes. Mit1 feedback inhibits Prm1. GOI, gene of interest. c) Changes in P‐Mb titer after overexpressing chaperones and P*_AOX1_* transcriptional activators. d) Changes in P‐Mb titer after deleting proteases. CK c,d) indicates the X33‐*Δku70* strain harboring the P‐Mb gene. SDS‐PAGE analyses c,d) are presented in Figure S1b (Supporting Information). e) The secretory expression of P‐Mb and S‐Hb and intracellular synthesis of V‐Hb and BM3_mut_ in the P1 strain. The molecular weight of P‐Mb, S‐Hb, V‐Hb, and BM3_mut_ containing 6 × His tags predicted by ExPASy were 17.91, 16.35, 16.60, and 118.76 KDa, respectively. Among them, V‐Hb is a homodimer composed of two identical subunits and two heme molecules, thus its molecular weight is 33.20 KDa. CK stands for the cell lysate of the X33‐*Δku70* strain without the hemoprotein gene, and M stands for protein ladder. Data presented as mean values ± SD from three independent biological replicates (*n* = 3). Statistical evaluation (*p*‐value) compared to the control was conducted by a two‐tailed t‐test. *p < 0.05, **p < 0.01, ***p < 0.001 and NS representing non‐significance (*p* ≥ 0.05).

First, P‐Mb was chosen as the model hemoprotein to construct an appropriate expressional system for globins because it had previously been expressed at a specific level.^[^ ^9^ ^]^ The effects of different factors on the expression of P‐Mb globin were investigated, including the type of *P. pastoris* host, the dosage of the integrated gene, and fermentation time. The results showed that the optimal expressional system for globins was based on the P*_AOX1_* promoter in the *P. pastoris* X33 host with a single copy of the P‐Mb gene, and the higher titer of P‐Mb (70.76 ± 8.15 mg L^−1^) was obtained at 48 h (**Figure** **2**a; Figure S1a, Supporting Information), which was 8.15‐fold higher than that of our previous report using P*_GAP_* (7.73 mg L^−1^).^[^ ^9^ ^]^ Furthermore, the analysis of cell lysates at 24, 48, 72, and 96 h showed that there was no intracellular accumulation of P‐Mb, indicating that the secretion of P‐Mb mediated by the α‐factor signal peptide was efficient (Figure S1a, Supporting Information).

Second, the effects of overexpressing P*_AOX1_* activators and chaperones on P‐Mb expression were investigated. The transcription of the P*_AOX1_*‐driven gene is regulated by a cascade of transcriptional activators,^[^ ^30^ ^]^ Mit1, Mxr1, and Prm1 (Figure 2b). In addition, protein disulfide isomerase (PDI) and endoplasmic reticulum‐resident chaperone (KAR2) can assist in correctly folding the target proteins,^[^ ^40^ ^]^ especially for the co‐translational folding of heme and apo‐hemoprotein (Figure 1). To improve the efficiency of gene integration, the key gene *ku70* responsible for the non‐homologous‐end‐joining repair mechanism^[^ ^41^ ^]^ was knocked out in the X33 strain. Subsequently, the cassettes of five genes (P*_GAP_*‐*PDI*, P*_GAP_*‐*KAR2*, P*_GAP_*‐*Mxr1*, P*_GAP_*‐*Mit1*, and P*_GAP_*‐*Prm1*) were integrated into the P*_AOX1_* UP‐gRNA2 locus^[^ ^41^ ^]^ of the X33‐*Δku70* strain genome, respectively. Comparatively, the higher titer of P‐Mb at 128.65 ± 8.27 mg L^−1^ was achieved by the X33‐*Δku70*‐P*_GAP_*‐*Mit1*‐(P‐Mb) strain, which is 74.37% higher than that of the control strain X33‐*Δku70*‐(P‐Mb) (Figure 2c; Figure S1b, Supporting Information). Next, the transcriptional level of P‐Mb was investigated in the test strain X33‐*Δku70*‐P*_GAP_*‐*Mit1*‐(P‐Mb) and the control strain X33‐*Δku70*‐(P‐Mb) using quantitative real‐time PCR (RT‐PCR). The results showed a 3.22‐fold increase in P‐Mb transcription in the X33‐*Δku70*‐P*_GAP_*‐*Mit1*‐(P‐Mb) strain compared to the X33‐*Δku70*‐(P‐Mb) strain. These findings indicated that overexpression of Mit1 enhanced the transcription of the P*_AOX1_*‐driven gene (Figure S2, Supporting Information).

Third, the titer of P‐Mb presented a trend of increasing and then decreasing from 24 to 96 h (Figure S1a, Supporting Information), indicating that longer fermentation times will result in more proteolytic digestion.^[^ ^42^ ^]^ Additionally, the secreted S‐Hb from *P. pastoris* is severely degraded during the fed‐batch fermentation,^[^ ^20^ ^]^ mainly due to the action of proteases produced by the organism. During fed‐batch fermentation, vacuolar aspartyl protease (Pep4) and GPI‐anchored aspartyl protease (Yps1‐1) were found in the secretome proteins.^[^ ^43^ ^]^ Moreover, the engineered *P. pastoris* with deleted *prb1* (encoding vacuolar serine protease) also exhibited low proteolytic activity.^[^ ^44^ ^]^ Therefore, these three proteases were deleted in the X33‐*Δku70* strain to examine their impacts on P‐Mb expression. Among five protease‐deficient strains (*Δpep4*, *Δyps1*, *Δprb1*, *Δpep4‐Δyps1*, and *Δpep4‐Δyps1‐Δprb1*), the higher titer of P‐Mb at 117.67 ± 15.24 mg L^−1^ was executed in the X33‐*Δku70*‐*Δyps1*‐(P‐Mb) strain, which was 56.79% higher than that of the control strain X33‐*Δku70*‐(P‐Mb) (Figure 2d; Figure S1b, Supporting Information). Subsequently, the time‐dependent behavior of the P‐Mb titer in the fermentation supernatant of the X33‐*Δku70*‐*Δyps1*‐(P‐Mb) strain was investigated. The results showed that the P‐Mb titer still reached its maximum at 48 h, while there was no significant difference between the titers of P‐Mb at 48, 72, and 96 h (*p* > 0.05). These findings indicated that knocking out the *yps1‐1* gene inhibited the degradation of globin (Figure S3, Supporting Information).

Finally, the *Mit1* gene was integrated into the X33‐*Δku70*‐*Δyps1* strain to construct the optimal platform for globin expression (P1 strain). The P1‐(P‐Mb) strain achieved the highest titer of P‐Mb at 162.46 ± 11.12 mg L^−1^ (Figure 2e), which was 26.28% and 38.06% higher than that in the X33‐*Δku70*‐P*_GAP_*‐*Mit1*‐(P‐Mb) and X33‐*Δku70*‐*Δyps1*‐(P‐Mb) strains, respectively. Considering the excellent ability of the P1 strain to express globin efficiently, the secretory expression of S‐Hb and intracellular synthesis of V‐Hb and BM3_mut_ (a double mutant A82F/A328F of wild‐type P450‐BM3 with enhanced catalytic activity^[^ ^16^ ^]^) were also attempted in the P1 strain. The results showed that the P1 strain was also suitable for expressing S‐Hb, V‐Hb, and BM3_mut_, with titers of 184.35 ± 17.14 mg L^−1^, 32.32 ± 5.94 mg L^−1^, and 28.31 ± 5.44 mg L^−1^, respectively (Figure 2e).

### 2.2 Removing the Spatial Segregation During Heme Biosynthesis

After the globin component was efficiently expressed, the supply of heme should be improved to synthesize holo‐hemoproteins. Although the intracellular deficiency of heme can be relieved through the exogenous addition of heme or its key precursor, 5‐aminolevulinic acid (ALA),^[^ ^9^, ^20^ ^]^ the high cost of ALA makes it unsuitable for large‐scale industrial production. In addition, because *P. pastoris* has a limited ability to uptake exogenous heme, despite supplementing heme at a concentration of 150 mg L^−1^, the heme‐binding ratio of the synthesized P‐Mb could only reach 22%.^[^ ^9^ ^]^ Hence, enhancing intracellular heme biosynthesis is a feasible approach for producing holo‐hemoproteins.^[^ ^19^, ^20^ ^]^

**Figure 3:**
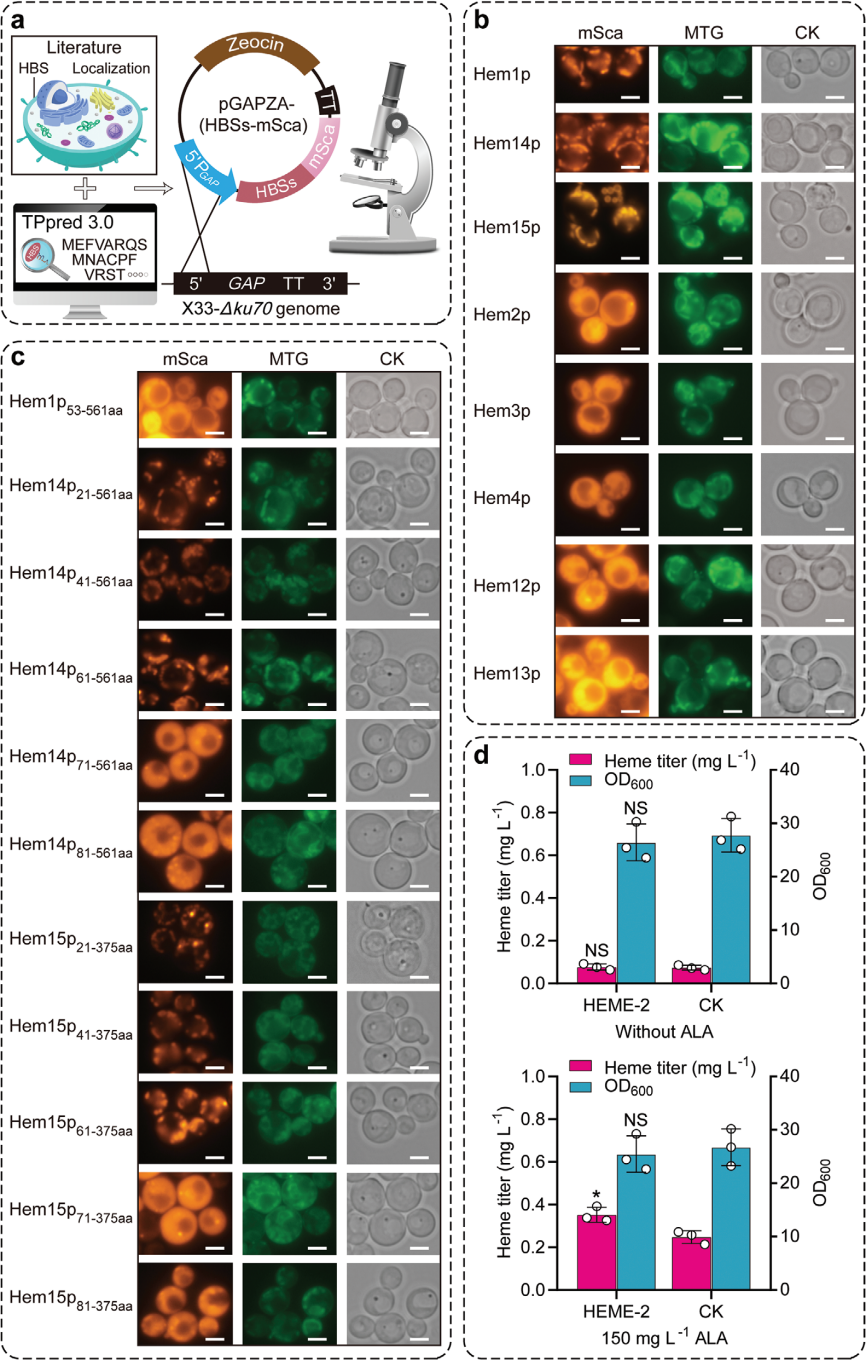
Reconstruction of the heme biosynthesis pathway in the cytoplasm. a) Schematic overview of validating the subcellular localization of HBS and their potential MLS. HBSs stands for HBS and their truncated mutants. b) Intracellular localization of native HBS by fluorescence microscopy analysis. CK indicates the recombinant strains of HBS fused with m‐Scarlet observed by white light in the same field of view. The scale bars represent 2 µm. c) Subcellular localization of N‐terminal truncated versions of Hem1p, Hem14p, and Hem15p. d) Effect of cytoplasmic co‐expression of *HEM14_71‐561_* and *HEM15_71‐375_* on cell growth and heme synthesis. CK represents the control strain X33‐*Δku70*. Data presented as mean values ± SD from three independent biological replicates (*n* = 3). Statistical evaluation (*p*‐value) compared to the control strain was conducted by a two‐tailed t‐test. *p < 0.05, **p < 0.01, ***p < 0.001 and NS representing non‐significance (*p* ≥ 0.05). Abbreviations: mSca, m‐Scarlet; MTG, Mitotracker Green FM; aa, amino acid.

As spatial segregation is a huge obstacle to synthesizing and utilizing heme in *P. pastoris*, it is necessary to determine the accurate intracellular localization of all HBS and the detailed MLS of HBS located in the mitochondria at first. Eight HBS were fused with the monomeric red fluorescent protein (m‐Scarlet) and integrated into the genome of the X33‐*Δku70* strain, respectively (**Figure** **3**a). Using mitochondrion‐specific fluorescent dyes (Mitotracker Green FM) and fluorescent microscopy, we found that five HBS were present in the cytoplasm (Hem2p, Hem3p, Hem4p, Hem12p, and Hem13p), while the other three enzymes (Hem1p, Hem14p, and Hem15p) were localized in the mitochondria (Figure 3b). This result was consistent with the previous observation in *S. cerevisiae*.^[^ ^19^ ^]^ In the following, the bioinformatic tool (TPpred 3.0, https://tppred3.biocomp.unibo.it) was used to predict the MLS of Hem1p, Hem14p, and Hem15p for relocating these three mitochondrial enzymes to the cytoplasm. However, only MLS_Hem1p_ could be predicated (52 residues in the N‐terminal). Thus, a series of N‐terminal truncated mutants of Hem1p, Hem14p, and Hem15p (Hem1p_53‐561aa_; Hem14p_21‐561aa_, Hem14p_41‐561aa_, Hem14p_61‐561aa_, Hem14p_71‐561aa_, and Hem14p_81‐561aa_; Hem15p_21‐375aa_, Hem15p_41‐375aa_, Hem15p_61‐375aa_, Hem15p_71‐375aa_, and Hem15p_81‐375aa_) were designed and fused with m‐Scarlet, respectively (Figure 3a). Compared with the localization of Hem1p, Hem14p_61‐561aa_, and Hem15p_61‐375aa_, it was found that Hem1p_53‐561aa_, Hem14p_71‐561aa_, and Hem15p_71‐375aa_ exhibited apparent changes in the subcellular localization from mitochondria to the cytoplasm (Figure 3c). This suggested that the real MLS_Hem1p_ was consistent with the prediction, and the MLS_Hem14p_ and MLS_Hem15p_ located between the 61 and 70 residues in the N‐terminal were also confirmed in *P. pastoris* for the first time.

The reconstruction of the heme biosynthetic pathway in the cytoplasm was attempted based on the detailed information on the subcellular localization and MLS of HBS. First, the transfer of ALA synthesis from mitochondria to the cytoplasm was performed by removing the MLS from Hem1p or introducing alternative ALA biosynthetic enzymes from bacteria^[^ ^45^ ^]^ (glutamyl‐tRNA reductase, GluTR; glutamate‐1‐semialdehyde 2,1‐aminomutase, GSAM; Figure S4a, Supporting Information). Six cytoplasmic ALA‐producing strains were constructed using plasmids pPICZA and pGAPZA (Figure S4b, Supporting Information), including P*_GAP_*‐*HEM1_53‐561_*, P*_AOX1_*‐*HEM1_53‐561_*, P*_GAP_*‐*GluTR_E_^fbr^*‐P*_GAP_*‐*GSAM_E_*, P*_AOX1_*‐*GluTR_E_^fbr^*‐P*_AOX1_*‐*GSAM_E_*, P*_GAP_*‐*GluTR_B_^fbr^*‐P*_GAP_*‐*GSAM_B_*, and P*_AOX1_*‐*GluTR_B_^fbr^*‐P*_AOX1_*‐*GSAM_B_* (*E*, *E. coli*; *B*, *Bacillus subtilis*; *GluTR^fbr^*, the feedback‐resistant version of *GluTR* ^[^ ^45^ ^]^). The results showed that the titers of ALA in these engineered strains were extremely low (Table S1, Supporting Information). In contrast, the control strains P*_GAP_*‐*HEM1* and P*_AOX1_*‐*HEM1* exhibited efficient synthesis of ALA, with titers of 100.09 ± 1.29 mg L^−1^ and 57.70 ± 8.80 mg L^−1^, respectively. This phenomenon may be caused by the substrate of Hem1p (succinyl‐CoA) predominantly existing in the mitochondria, making it difficult to be utilized by the MLS‐truncated Hem1p in the cytoplasm. In addition, the low expressional level limited the application of these bacterial ALA biosynthetic enzymes to synthesize heme in *P. pastoris*.^[^ ^19^ ^]^ Hence, the synthesis of ALA in the mitochondria should be maintained during the reconstruction of the heme biosynthetic pathway. To facilitate the accumulation of ALA, our previous study investigated the effect of multi‐copy integrated expression of the *HEM1* gene on ALA synthesis in *S. cerevisiae*.^[^ ^19^ ^]^ However, the results showed no significant difference in intracellular heme levels between low‐ and high‐copy expression of *HEM1*. Therefore, a single cassette of native *HEM1* (P*_GAP_*‐*HEM1*) was incorporated into the *AOXTT*DOWN‐gRNA locus^[^ ^41^ ^]^ of the X33‐*Δku70* genome, generating the HEME‐1 strain.

Next, the functional expression of the mitochondrial enzymes Hem14p and Hem15p was assessed in the cytoplasm. The HEME‐2 strain was generated by replacing the wild‐type *HEM14* and *HEM15* genes in the X33‐*Δku70* genome with their MLS‐truncated versions, *HEM14_71‐561_* and *HEM15_71‐375_*. The results showed that the co‐expression of the *HEM14_71‐561_* and *HEM15_71‐375_* genes could synthesize sufficient heme in the cytoplasm to maintain cell growth without the addition of ALA (Figure 3d). Furthermore, in the presence of 150 mg L^−1^ of ALA supplementation, the HEME‐2 strain also displayed a significant increase in the intracellular titer of heme (41.94%) compared to the control strain X33‐*Δku70*. This result indicated that Hem14p_71‐561aa_‐Hem15p_71‐375aa_ could utilize their substrates (PPG IX and PP IX) more efficiently to synthesize heme (Figure 3d).

### 2.3 Eliminating the Rate‐Limiting Steps During Heme Biosynthesis

**Figure 4:**
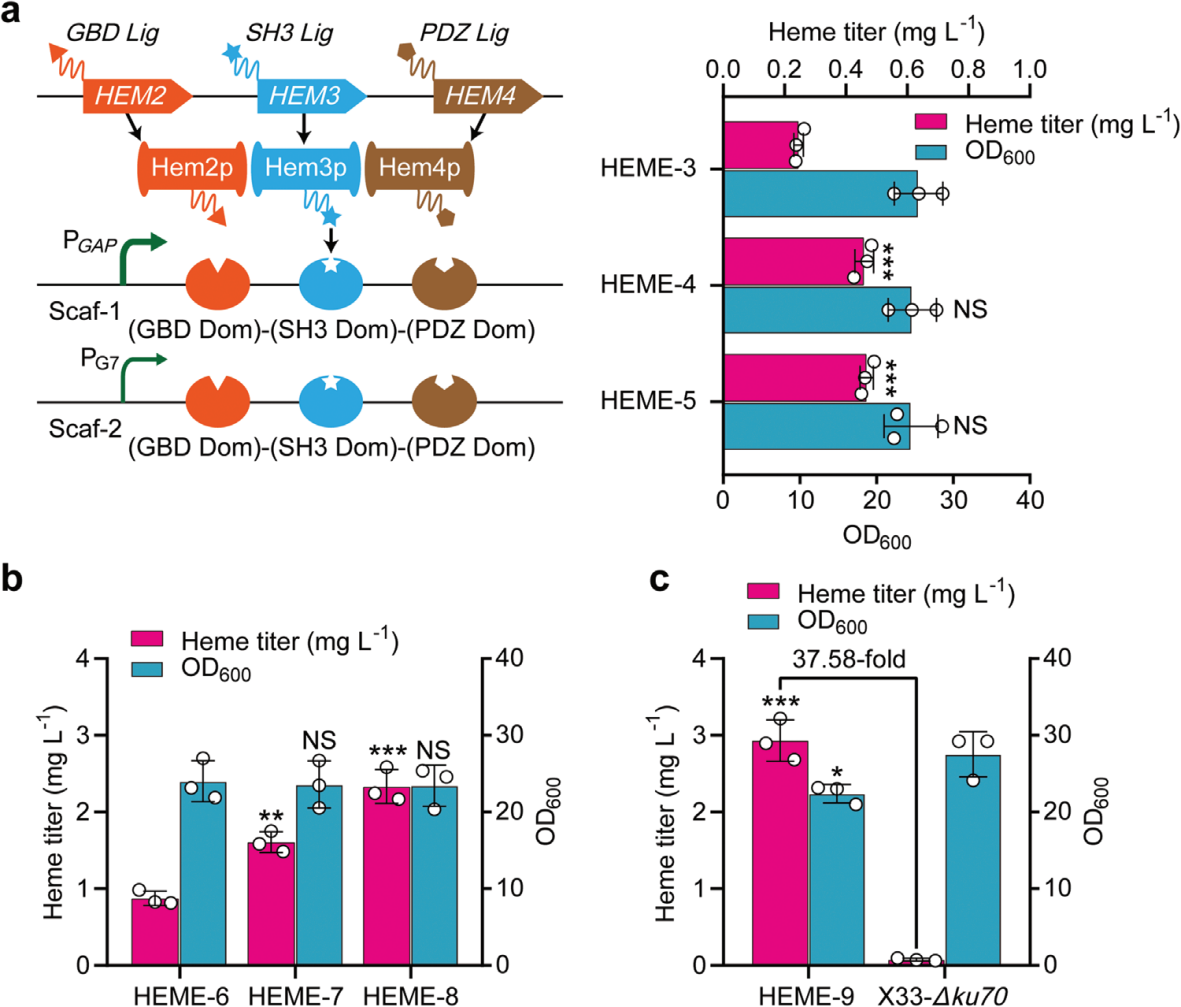
Moderate enhancement of the reconstructed heme biosynthesis pathway. a) Enhancement of key steps in heme biosynthesis (Hem2p, Hem3p, and Hem4p) using multi‐enzyme assembly. Hem2p, Hem3p, and Hem4p were fused with specific ligands and fixed to protein scaffolds driven by the constitutive promoters P*_GAP_* (medium strength, HEME‐4 strain) and P_G7_ (weak, HEME‐5 strain).^[^ ^46^ ^]^ The HEME‐3 strain without the synthetic protein scaffold was used as the control. b) The effects of overexpressing *HEM13* (HEME‐7 strain) and deleting *HMX1* (HEME‐8 strain) on the intracellular accumulation of heme. HEME‐6 indicates the control strain. c) Comparison between the final heme‐supplying strain HEME‐9 and the original strain X33‐*Δku70* in heme biosynthesis. Data presented as mean values ± SD from three independent biological replicates (*n* = 3). Statistical evaluation (*p*‐value) compared to the control strain was conducted by a two‐tailed t‐test. *p < 0.05, **p < 0.01, ***p < 0.001 and NS representing non‐significance (*p* ≥ 0.05). Abbreviations: Scaf, scaffold; Lig, ligand; Dom, domain.

To moderately enhance the biosynthesis of heme, it is essential to further eliminate the rate‐limiting steps in the reconstructed heme biosynthetic pathway. According to previous studies, Hem2p, Hem3p, and Hem4p have been identified as the rate‐limiting steps during heme biosynthesis in yeast.^[^ ^35^, ^36^, ^37^ ^]^ Hence, protein scaffolds^[^ ^39^ ^]^ were used to spatially assemble these three HBS to optimize substrate trafficking and facilitate metabolic flux (**Figure** **4**a). The native *HEM2*, *HEM3*, and *HEM4* genes in the genome of X33‐*Δku70* were replaced by their corresponding genes fused with specific ligands (*HEM2‐GBD* ligand, *HEM3‐SH3* ligand, and *HEM4‐PDZ* ligand),^[^ ^39^ ^]^ generating the HEME‐3 strain. To avoid potential adverse impacts of excess protein scaffolds on the expression of hemoproteins, protein scaffolds containing metazoan signaling protein interaction domains (GBD, SH3, and PDZ)^[^ ^39^ ^]^ were integrated into the P*_TEF1_* UP‐gRNA1 locus^[^ ^41^ ^]^ of the HEME‐3 genome using P*_GAP_* and a modified weak constitutive promoter P_G7_,^[^ ^46^ ^]^ respectively (HEME‐4 and HEME‐5 strains, Figure 4a). Due to the overexpression of the native *HEM1* gene that could produce 100.09 ± 1.29 mg L^−1^ of ALA (Table S1, Supporting Information), 150 mg L^−1^ of ALA was exogenously added to examine the effects of protein scaffolds driven by P*_GAP_* and P_G7_ on heme biosynthesis. When supplemented with 150 mg L^−1^ of ALA, the HEME‐4 and HEME‐5 strains showed an 86.99% and 90.24% enhancement in heme accumulation, respectively, compared to the HEME‐3 strain (Figure 4a). The results indicated that both synthetic protein scaffolds driven by P*_GAP_* and P_G7_ could significantly boost heme synthesis. Therefore, the HEME‐5 strain was selected for the following metabolic engineering.

Combining the strategies applied to HEME‐1 (overexpression of *HEM1*), HEME‐2 (cytoplasmic localization of Hem14p and Hem15p), and HEME‐5 (assembly of Hem2p, Hem3p, and Hem4p) strains, HEME‐6 strain was constructed. In addition, it was reported that the overexpression of the *HEM13* gene could enhance heme synthesis,^[^ ^34^ ^]^ and heme degradation catalyzed by Hmx1p (heme oxygenase) in yeast was detrimental to the synthesis of hemoproteins.^[^ ^36^ ^]^ Therefore, strategies of replacing the native promoter of the *HEM13* gene with P*_GAP_* to increase its expression and knocking out the *HMX1* gene were implemented in the HEME‐6 strain, allowing for a 1.83‐fold and 2.66‐fold titer of heme in HEME‐7 and HEME‐8 strains, respectively (Figure 4b). This indicated that the increased expression of Hem13p and the deletion of the *HMX1* gene could significantly improve intracellular heme supply. Subsequently, the knockout of *HMX1* was performed in the HEME‐7 strain to generate the final heme‐supplying strain HEME‐9. As shown in Figure 4c, the HEME‐9 strain achieved the highest heme titer of 2.93 ± 0.27 mg L^−1^, which was 37.58‐fold higher than that of the original strain X33‐*Δku70*. Thus, the HEME‐9 strain was selected to synthesize hemoproteins in the subsequent experiments.

### 2.4 The Biochemical Properties of Biosynthetic Hemoproteins

**Figure 5:**
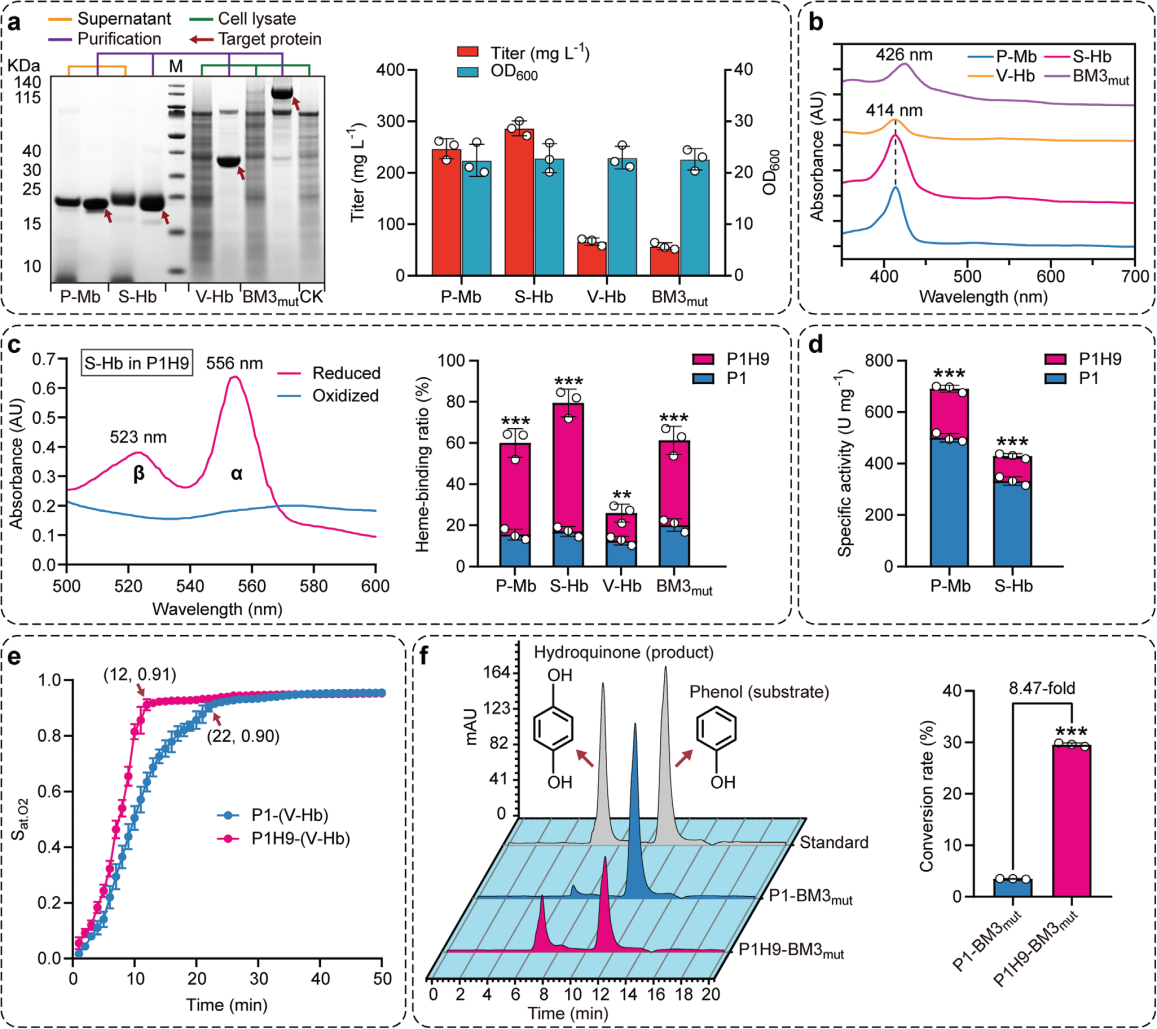
The biochemical properties of biosynthetic hemoproteins. a) The expression of several hemoproteins in the P1H9 strain. CK stands for the cell lysate of the X33‐*Δku70* strain without the hemoprotein gene, and M stands for protein ladder. b) The spectral characteristics of purified hemoproteins. c) Detection of the heme‐binding ratio of purified hemoproteins using the difference spectrum between reduced and oxidized samples.^[^ ^54^ ^]^ d) The specific POD activity of purified P‐Mb and S‐Hb. e) The time‐course oxygen binding behavior of V‐Hb in the presence of sodium dithionite (reductant and oxygen scavenger). *S*_at.O2_ stands for oxygen saturation degree.^[^ ^55^ ^]^ f) HPLC analysis of the catalytic efficiency of cells expressing BM3_mut_ in converting phenol to hydroquinone. Data presented as mean values ± SD from three independent biological replicates (*n* = 3). Statistical evaluation (*p*‐value) compared to the control strain was conducted by a two‐tailed t‐test. *p < 0.05, **p < 0.01, ***p < 0.001 and NS representing non‐significance (*p* ≥ 0.05).

To efficiently synthesize highly active holo‐hemoproteins, the strategies employed in the P1 strain were applied in the heme‐supplying strain HEME‐9, generating the final production strain P1H9. Due to the sufficient intracellular availability of heme, the synthesis of various hemoproteins was significantly enhanced in the P1H9 strain compared to the P1 strain, including an increase of 52.01% in P‐Mb (246.95 ± 19.46 mg L^−1^), 55.43% in S‐Hb (286.53 ± 14.29 mg L^−1^), 1.05‐fold in V‐Hb (66.30 ± 7.05 mg L^−1^), and 1.02‐fold in BM3_mut_ (57.27 ± 6.79 mg L^−1^), respectively (**Figure** **5**a).

As the extensive applications of these hemoproteins in the fields of artificial meat alternatives,^[^ ^10^, ^47^ ^]^ high‐cell‐density fermentation,^[^ ^24^, ^25^, ^26^ ^]^ and biocatalysts,^[^ ^13^, ^14^, ^15^, ^16^ ^]^ it is imperative to investigate their biochemical properties thoroughly. First, the absorption spectrum of these hemoproteins was detected between 350 and 700 nm (Figure 5b). It was found that P‐Mb, S‐Hb, and V‐Hb exhibited an intense Soret absorption band at 414 nm, which was consistent with the previous studies that the incorporated heme in globin was responsible for the characteristic spectrum.^[^ ^18^, ^19^, ^48^ ^]^ However, the Soret band for BM3_mut_ was slightly red‐shifted (λ = 426 nm) compared to wild‐type BM3 (λ = 418 nm),^[^ ^49^ ^]^ indicating that the double mutation of A82F/A328F may affect its incorporation with heme. Second, the heme‐binding ratios of these hemoproteins synthesized in both the P1 and P1H9 strains were investigated (Figure 5c). From the P1 to P1H9 strains, the heme‐binding ratios of P‐Mb, S‐Hb, V‐Hb, and BM3_mut_ improved by 2.87‐fold, 3.67‐fold, 1.07‐fold, and 2.04‐fold, respectively, reaching 60.02 ± 6.98%, 79.47 ± 6.72%, 25.94 ± 4.36%, and 61.27 ± 6.86%. Among them, the elevated heme‐binding ratio of V‐Hb was not very significant, which may be related to the low assembly efficiency of apo‐(V‐Hb) with heme at 30 °C.^[^ ^48^ ^]^

Finally, the functional activity of these biosynthetic hemoproteins was evaluated. Due to its peroxidase (POD) activity, Mb is often designed as artificial metalloenzymes or biocatalysts for dyeing,^[^ ^50^ ^]^ dye decolorization,^[^ ^51^ ^]^ biodegradation,^[^ ^52^ ^]^ and functionalization reactions.^[^ ^53^ ^]^ In addition, the POD activity of S‐Hb has also been identified.^[^ ^20^ ^]^ According to Figure 5d, the specific POD activities of P‐Mb and S‐Hb increased by 38.10% and 29.31% from the P1 to P1H9 strains, respectively, reaching 691.13 ± 13.37 U mg^−1^ and 429.35 ± 9.42 U mg^−1^. In addition, the oxygen‐binding property of V‐Hb was investigated. As shown in Figure 5e, the transition from deoxy‐ to fully‐oxygenated state occurred more rapidly in the biosynthetic V‐Hb by the P1H9 strain (12 min, 91.16 ± 2.00% oxygenated) than in the V‐Hb synthesized in the P1 strain (22 min, 90.22 ± 1.53% oxygenated), indicating that the oxygen‐binding capacity of V‐Hb was enhanced. Moreover, the catalytic efficiency of converting phenol into hydroquinone by BM3_mut_ expressed in the P1 and P1H9 strains was analyzed. It was found that the whole‐cell BM3_mut_ catalytic efficiency of the P1H9 strain was equivalent to 8.47 times that of the P1 strain, reaching 29.56 ± 0.33% (Figure 5f). Consequently, these results suggest that improving intracellular heme supply is beneficial for producing high‐active hemoproteins.

## 3 Discussion

Microbial chassis are essential platforms for eco‐friendly and sustainable biomanufacturing. *P. pastoris* has received much attention for its advantages in the secretory expression of proteins and its powerful capacity for methanol utilization. It has been widely used to produce recombinant proteins,^[^ ^21^ ^]^ industrial enzymes,^[^ ^22^, ^56^ ^]^ drugs,^[^ ^57^, ^58^ ^]^ and natural products.^[^ ^59^, ^60^, ^61^ ^]^ However, the synthesis of various hemoproteins in *P. pastoris* still faces many challenges, such as the low expressional level of globins,^[^ ^9^ ^]^ the severe degradation of globins,^[^ ^20^ ^]^ the insufficient heme supply,^[^ ^19^, ^48^ ^]^ and the metabolic burden and impaired growth caused by multi‐copy integrated expression of globin gene and overexpression of all genes involved in heme biosynthesis.^[^ ^20^ ^]^ To address these issues, a powerful expressional platform for the globin component was constructed in this study, based on the strong transcription of the single‐copy globin gene driven by the P*_AOX1_* promoter and its most effective activator, Mit1, as well as the weak degradation of globin. In addition, moderate enhancement of heme supply under low metabolic load was achieved by removing spatial segregation and strengthening key steps during heme biosynthesis. Using the suitable expressional platform for globin and the optimized heme supply, we established a *P. pastoris* chassis that produced several highly active hemoproteins (P‐Mb, S‐Hb, V‐Hb, and P450‐BM3). These hemoproteins can be used for the development of plant‐based meat, high‐cell density fermentation, and whole‐cell catalysis for synthesizing high‐value‐added compounds.

Although Impossible Foods Inc. previously produced S‐Hb by overexpressing the transcriptional activator Mxr1 for P*_AOX1_* in *P. pastoris*,^[^ ^23^ ^]^ Mxr1 may not be the optimal choice among the three activators (Mit1, Mxr1, and Prm1). In this study, we investigated the contributions of these three activators to the synthesis of P‐Mb and found that Mit1 was the most effective, resulting in a 74.37% increase in P‐Mb titer when overexpressed (Figure 2c). In addition, a previous study demonstrated that the α‐globin subunit of human‐Hb was susceptible to degradation during intracellular synthesis in *S. cerevisiae*. Deleting the *pep4* gene could prevent this degradation.^[^ ^36^ ^]^ Here we discovered that proteolytic digestion occurred during the secretory expression of P‐Mb, and knocking out the *pep4* gene did not resolve this issue. However, targeting the *yps1‐1* gene resulted in a 56.79% increase in P‐Mb titer (Figure 2d). This suggested that the GPI‐anchored aspartyl protease Yps1‐1 was primarily responsible for degrading globin during the secretory process, and deleting its gene reduced endogenous protease activity and extracellular protease levels. In the latest study, S‐Hb, maize‐Hb, rice‐Hb, and *S. cerevisiae*‐Hb were successfully synthesized in *Corynebacterium glutamicum*.^[^ ^62^ ^]^ Among them, the highest expression of S‐Hb, comprising 20% of the total protein, was achieved using a high‐throughput screening method based on the fusion of Hb with a green fluorescent protein. This method optimized the N‐terminal coding sequence of Hb genes, native inducible promoters, and plasmid copy number. However, the real titer of these hemoproteins remains unclear. In our research, the higher titers of P‐Mb, S‐Hb, V‐Hb, and P450‐BM3 were achieved at shake‐flask level.

Currently, the main strategy to enhance heme supply in *P. pastoris* is to overexpress all genes involved in heme biosynthesis.^[^ ^20^, ^23^ ^]^ However, a recent study found that a significant accumulation of heme intermediates (PBG, HMB, CPG III, PPG IX, and PP IX) rather than heme was present in the fermentation products from an engineered *P. pastoris* strain that overexpressed all heme biosynthetic pathway genes through an analysis of ESI‐MS/MS.^[^ ^38^ ^]^ These results indicated that this metabolic strategy was not suitable for hemoprotein production in *P. pastoris*. The expression of eight HBS should be optimized rather than overexpressed to reduce the accumulation of heme intermediates and potential metabolic burden to the host. Furthermore, the latest study showed that the heme‐binding ratio of S‐Hb, synthesized in C*. glutamicum*, could reach 28% by adding 1 g L^−1^ ALA to the fermentation medium.^[^ ^62^ ^]^ However, this heme‐binding ratio is still low, and this approach would increase the culture cost by over 60%.^[^ ^16^ ^]^ Therefore, in this study, the rate‐limiting steps catalyzed by Hem2p, Hem3p, and Hem4p were eliminated using protein scaffolds, promoting the transport of their substrates (ALA, PBG, and HMB) and improving the heme titer (90.24%, Figure 4a). Subsequently, the accumulation of the heme intermediate CPG III was alleviated through the overexpression of Hem13p (heme titer increased by 83.45%, Figure 4b). Finally, the relocation of Hem14p and Hem15p from the mitochondria into the cytoplasm by truncating their MLS was performed, increasing the utilization efficiency of their substrates (PPG IX and PP IX) and heme titer (41.94%, Figure 3d). By implementing these strategies, the final titer of heme was enhanced by 37.58‐fold, resulting in the titers of P‐Mb, S‐Hb, V‐Hb, and BM3_mut_ increased by 52.01%, 55.43%, 1.05‐fold, and 1.02‐fold, respectively (Figure 5). Moreover, the heme‐binding ratio of P‐Mb, S‐Hb, V‐Hb, and BM3_mut_ showed an increase of 2.87‐fold, 3.67‐fold, 1.07‐fold, and 2.04‐fold, respectively, reaching 60.02 ± 6.98%, 79.47 ± 6.72%, 25.94 ± 4.36%, and 61.27 ± 6.86%. Additionally, the specific POD activities of P‐Mb and S‐Hb, as well as the oxygen‐binding capability of V‐Hb (oxygen saturation per minute), were improved by 38.10%, 29.31%, and 85.24%, respectively. It is worth noting that the whole‐cell catalytic activity of BM3_mut_ exhibited a 7.47‐fold increase.

Based on the engineered *P. pastoris* chassis developed here, the N‐glycosylation and O‐glycosylation pathways within the host can be further designed^[^ ^63^, ^64^ ^]^ to mitigate the slight glycosylation of the P‐Mb and S‐Hb during secretory expression (Figures 2e and 5a). Finally, maintaining the balance between globin expression and heme synthesis is expected to further improve the activity of hemoproteins.^[^ ^16^ ^]^ The heme‐sensing regulators and CRISPRi/or sRNA can be combined to further develop heme‐sensitive biosensors for fine‐tuning the enhanced heme biosynthesis pathway. In conclusion, our study demonstrates the feasibility of producing high‐active hemoproteins through the metabolic engineering of the complicated heme biosynthetic pathway. Moreover, the engineered strains and strategies described here can be helpful in producing other hemoproteins with high activities.

## 4 Experimental Section

### Chemicals and Reagents

PrimeSTAR HS DNA polymerase used for PCR was purchased from Takara (Dalian, China). The kits for genomic DNA and plasmid extraction, and DNA gel purification were purchased from Takara, Sangon (Shanghai, China), and Thermo Scientific (Shanghai, China), respectively. Sangon performed oligonucleotide synthesis and Sanger sequencing. ALA, methanol, and acetonitrile were purchased from Sigma‐Aldrich (St. Louis, MO, USA). Phenol and hydroquinone were obtained from Macklin (Shanghai, China). Other chemicals were purchased from Sangon unless otherwise specified.

### Plasmids and the Cassettes for Gene Knocking‐In/Out

The plasmids, genes, and promoters involved in this study are shown in Table S2 (Supporting Information). The gene knock‐in/out cassettes and sgRNA utilized in this study are presented in Tables S3 and S4 (Supporting Information), respectively. The primers used in this study are listed in Table S5 (Supporting Information). The sequences of heterologous genes, P_G7_ promoter, and original CRISPR/Cas9 plasmid pPIC3.5K‐*ku70*‐gRNA1 included in this study were outlined in Notes, Supporting Information.

To construct a suitable expression system for the globin component in hemoproteins, the P‐Mb, S‐Hb, and V‐Hb genes were codon‐optimized and synthesized by GenScript (Nanjing, China). The BM3_mut_ gene was synthesized and expressed heterologously in the previous study.^[^ ^16^ ^]^ These hemoprotein genes were amplified with primers PMb‐F/PMb‐R, SHb‐F/SHb‐R, VHb‐F/VHb‐R, and BM3‐F/BM3‐R, respectively. Their corresponding backbone fragments were obtained by PCR using primers pPICZ‐PMb‐F/pPICZ‐PMb‐R, pPICZ‐SHb‐F/pPICZ‐SHb‐R, pPICZ‐VHb‐F/pPICZ‐VHb‐R, and pPICZ‐BM3‐F/pPICZ‐BM3‐R, respectively, with plasmid pPICZαA as the template. These hemoprotein genes carrying 6 × His tags and their corresponding backbone fragments were assembled using Gibson assembly^[^ ^65^ ^]^ to generate plasmids pPICZαA‐(P‐Mb)_cn = 1_, pPICZαA‐(S‐Hb)_cn = 1_, pPICZA‐(V‐Hb)_cn = 1_, and pPICZA‐(BM3_mut_)_cn = 1_ (cn, copy number). The multimers pPICZαA‐(P‐Mb)_cn = 2_ and pPICZαA‐(P‐Mb)_cn = 3_, which were used to construct double‐ and three‐copy *P. pastoris* strains of P‐Mb, were generated in vitro by digesting and assembling pPICZαA‐(P‐Mb)_cn = 1_ with isocaudamer *Bgl* II and *Bam*H I enzymes (Invitrogen manual).

To construct the knock‐in cassette of the P*_AOX1_* transcriptional activator gene *PDI*, the upstream and downstream homologous arms of the P*_AOX1_* UP‐gRNA2 (Ag2)^[^ ^41^ ^]^ locus (1 kb), P*_GAP_* promoter, *PDI* gene, and *AOX1* terminator (*AOX1TT*) were amplified with primers UpAg2‐F/UpAg2‐R, DoAg2‐F/DoAg2‐R, pGAP‐Ag2‐F/pGAP‐PDI‐R, PDI‐F/PDI‐R, and AOXTT‐PDI‐F/AOXTT‐Ag2‐R, respectively, with X33 genomic DNA and plasmid pGAPZαA as templates. The amplified products were fused using overlap extension PCR^[^ ^66^ ^]^ to generate the knock‐in cassette of the *PDI* gene, UpAg2‐P*_GAP_*‐*kozak*‐*PDI*‐*AOX1TT*‐DoAg2. Kozak sequence^[^ ^67^ ^]^ was used here, equivalent to the ribosome binding site in prokaryotes. Following this same way, gene knock‐in cassettes of other transcriptional activators (*KAR2*, *Mxr1*, *Mit1*, and *Prm1*) were constructed. The plasmid pPIC3.5K‐P*_AOX1_* UP‐gRNA2 was constructed by replacing the sgRNA sequence in the original CRISPR/Cas9 plasmid pPIC3.5K‐*ku70*‐gRNA1^[^ ^41^ ^]^ with primers pAOX1UP‐gRNA2‐F1/Cas9‐backbone‐R1 and Cas9‐backbone‐F2/pAOX1UP‐gRNA2‐R2.

To construct the knock‐out cassette of the protease gene *pep4*, the upstream and downstream homologous arms of the *pep4* gene (1 kb) were amplified with primers Uppep4‐F/Uppep4‐R and Dopep4‐F/Dopep4‐R, respectively, with X33 genomic DNA as the template. The amplified products were fused by overlap extension PCR to generate the knock‐out cassette of the *pep4* gene, Uppep4‐Dopep4. Subsequently, the same approach constructed the gene knock‐out cassettes of other proteases (*prb1* and *yps1‐1*), and gRNA targets of these protease genes were selected using CHOPCHOP (http://chopchop.cbu.uib.no/) to generate CRISPR/Cas9 plasmids pPIC3.5K‐*pep4*‐gRNA, pPIC3.5K‐*yps1*‐gRNA, and pPIC3.5K‐*prb1*‐gRNA.

To verify the subcellular localization of HBSs, the m‐Scarlet gene was codon‐optimized and synthesized by GenScript. The m‐Scarlet fragments were amplified using primers mScarlet‐H1‐F/mScarlet‐R, mScarlet‐H2‐F/mScarlet‐R, mScarlet‐H3‐F/mScarlet‐R, mScarlet‐H4‐F/mScarlet‐R, mScarlet‐H12‐F/mScarlet‐R, mScarlet‐H13‐F/mScarlet‐R, mScarlet‐H14‐F/mScarlet‐R, and mScarlet‐H15‐F/mScarlet‐R, respectively. The HBSs genes are amplified using primers listed in Table S5 (Supporting Information), with X33 genomic DNA as the template. The backbone fragment was obtained by PCR using primers pGAPZ‐F/pGAPZ‐R with plasmid pGAPZαA as the template. The backbone fragment, HBSs genes, and m‐Scarlet were assembled via Gibson assembly to generate plasmids pGAPZA‐*HBSs*‐*linker*‐*mSca* (Linker consists of flexible amino acids Gly‐Ala‐Gly‐Ala‐Gly‐Ala‐Gly‐Ala‐Gly‐Ala).

To examine the transfer of ALA synthesis from mitochondria to the cytoplasm, the genes of *HEM1*, *HEM1_53‐561_*, *GluTR_E_^fbr^*, *GSAM_E_*, *GluTR_B_^fbr^*, and *GSAM_B_* were inserted into the plasmids pPICZA and pGAPZA. These genes were amplified using primers pPICZ_HEM1‐F/pPICZ_HEM1‐R, pPICZ_Δ52HEM1‐F/pPICZ_HEM1‐R, pPICZ_GluTR_E_^fbr^‐F/pPICZ_GluTR_E_^fbr^‐R, pPICZ_GSAM_E_‐F/pPICZ_GSAM_E_‐R, pPICZ_GluTR_B_^fbr^‐F/pPICZ_GluTR_B_^fbr^‐R, pPICZ_GSAM_B_‐F/pPICZ_GSAM_B_‐R, pGAPZ_HEM1‐F/pGAPZ_HEM1‐R, pGAPZ_Δ52HEM1‐F/pGAPZ_HEM1‐R, pGAPZ_GluTR_E_^fbr^‐F/pGAPZ_GluTR_E_^fbr^‐R, pGAPZ_GSAM_E_‐F/pGAPZ_GSAM_E_‐R, pGAPZ_GluTR_B_^fbr^‐F/pGAPZ_GluTR_B_^fbr^‐R, and pGAPZ_GSAM_B_‐F/pGAPZ_ GSAM_B_‐R, respectively, with the genomic DNA of X33, *E. coli* BL21(DE3), and *B. subtilis* 168 as templates. The backbone fragments were obtained by PCR using primers pPICZ‐F/pPICZ‐R and pGAPZ‐F/pGAPZ‐R, respectively, with plasmid pPICZαA and pGAPZαA as templates. The backbone fragments and these genes were assembled by Gibson assembly to generate plasmids pPICZA‐*HEM1*, pPICZA‐*HEM1_53‐561_*, pPICZA‐*GluTR_E_^fbr^*, pPICZA‐*GSAM_E_*, pPICZA‐*GluTR_B_^fbr^*, pPICZA‐*GSAM_B_*, pGAPZA‐*HEM1*, pGAPZA‐*HEM1_53‐561_*, pGAPZA‐*GluTR_E_^fbr^*, pGAPZA‐*GSAM_E_*, pGAPZA‐*GluTR_B_^fbr^*, and pGAPZA‐*GSAM_B_*. The multimers pPICZA‐*GluTR_E_^fbr^*‐*GSAM_E_*, pPICZA‐*GluTR_B_^fbr^*‐*GSAM_B_*, pGAPZA‐*GluTR_E_^fbr^*‐*GSAM_E_*, and pGAPZA‐*GluTR_B_^fbr^*‐*GSAM_B_* were constructed in vitro by digesting and assembling pPICZA‐*GluTR_E_^fbr^*, pPICZA‐*GSAM_E_*, pPICZA‐*GluTR_B_^fbr^*, pPICZA‐*GSAM_B_*, pGAPZA‐*GluTR_E_^fbr^*, pGAPZA‐*GSAM_E_*, pGAPZA‐*GluTR_B_^fbr^*, and pGAPZA‐*GSAM_B_* with isocaudamer *Bgl* II and *Bam*H I enzymes (Invitrogen manual). To construct the knock‐in cassette of native *HEM1*, the upstream and downstream homologous arms of the *AOXTT*DOWN‐gRNA (ADo)^[^ ^41^ ^]^ locus (1 kb), P*_GAP_* promoter, and *HEM1* gene were amplified using primers UpADo‐F/UpADo‐R, DoADo‐F/DoADo‐R, pGAP‐ADo‐F/pGAP‐HEM1‐R, and HEM1‐F/HEM1‐R, respectively, with X33 genomic DNA and plasmid pGAPZαA as templates. These amplified fragments were fused by overlap extension PCR to generate the knock‐in cassette of the *HEM1* gene, UpADo‐P*_GAP_*‐*kozak*‐*HEM1*‐DoADo. The downstream homologous arm of the ADo locus carries the *AOX1TT*. Based on the Cas9 backbone of pPIC3.5K‐*ku70*‐gRNA1 and gRNA screening by CHOPCHOP website, the CRISPR/Cas9 plasmid pPIC3.5K‐*AOXTT*DOWN‐gRNA was produced.

To achieve the cytoplasmic co‐expression of Hem14p_71‐561aa_ and Hem15p_71‐375aa_, the upstream and downstream homologous arms of MLS_Hem14p_ and MLS_Hem15p_ (1 kb) were amplified using primers UpH14‐F/UpH14‐R, DoH14‐F/DoH14‐R, UpH15‐F/UpH15‐R, and DoH15‐F/DoH15‐R, respectively, with X33 genomic DNA as the template. These amplified products were fused by overlap extension PCR to generate knock‐out cassettes of MLS_Hem14p_ and MLS_Hem15p_, UpHEM14‐DoHEM14 and UpHEM15‐DoHEM15. Subsequently, gRNA targets of MLS_Hem14p_ and MLS_Hem15p_ were selected using the CHOPCHOP website to replace the sgRNA sequence in pPIC3.5K‐*ku70*‐gRNA1, resulting in CRISPR/Cas9 plasmids pPIC3.5K‐*HEM14*‐gRNA and pPIC3.5K‐*HEM15*‐gRNA.

To assemble three key cascade enzymes (Hem2p, Hem3p, and Hem4p) by protein‐guided scaffolds for improving heme synthesis, the genes of *HEM2*, *HEM3*, and *HEM4*, which were fused with specific ligands, replaced the native *HEM2*, *HEM3*, and *HEM4* in the X33‐*Δku70* genome. The *HEM2*‐*linker*‐*GBD ligand* fragment was obtained by three rounds of PCR using primers H2‐F/H2LG‐R1, H2‐F/H2LG‐R2, and H2‐F/H2LG‐R3, respectively, with X33 genomic DNA and amplified products of first and second PCR as templates. The upstream and downstream homologous arms of *HEM2* (1 kb) were amplified by primers UpH2‐F/UpH2‐R and DoH2‐F/DoH2‐R, respectively, with X33 genomic DNA as the template. These amplified fragments were fused by overlap extension PCR to generate the knock‐in cassette of the *HEM2* gene, UpH2‐*HEM2*‐*linker*‐*GBD ligand*‐DoH2 (Linker consists of flexible amino acids Gly‐Ser‐Gly‐Ser‐Gly‐Ser‐Gly‐Ser‐Gly). Using the same method, the other two gene knock‐in cassettes, UpH3‐*HEM3*‐*linker*‐*SH3 ligand*‐DoH3 and UpH4‐*HEM4*‐*linker*‐*PDZ ligand*‐DoH4 were constructed. Using CHOPCHOP, gRNA targets for the *HEM2*, *HEM3*, and *HEM4* genes were obtained. Next, CRISPR/Cas9 plasmids pPIC3.5K‐*HEM2*‐gRNA, pPIC3.5K‐*HEM3*‐gRNA, and pPIC3.5K‐*HEM4*‐gRNA were generated by replacing the sgRNA sequence in pPIC3.5K‐*ku70*‐gRNA1 with primers listed in Table S5 (Supporting Information). To construct the knock‐in cassettes of protein scaffolds driven by P*_GAP_* and P_G7_ promoters, the P_G7_ promoter and the codon‐optimized gene encoding the fusion GBD domain‐Linker‐SH3 domain‐Linker‐PDZ domain were synthesized by GenScript. The upstream and downstream homologous arms of P*_TEF1_* UP‐gRNA1 (Tg1) locus^[^ ^41^ ^]^ (1 kb), the P*_GAP_* and P_G7_ promoters, the domain fusion, and *AOX1TT* fragments were generated by PCR using primers UpTg1‐F/UpTg1‐R, DoTg1‐F/DoTg1‐R, pGAP_G7‐Tg1‐F/pGAP‐GSP‐R, pGAP_G7‐Tg1‐F/pG7‐GSP‐R, GSP‐F/GSP‐R, and AOXTT‐GSP‐F/AOXTT‐Tg1‐R, respectively, with X33 genomic DNA, plasmid pGAPZαA, and synthetic P_G7_ promoter and domain fusion as templates. These gene fragments were fused to generate UpTg1‐P*_GAP_*‐*kozak*‐*GBD domain*‐*linker*‐*SH3 domain*‐*linker*‐*PDZ domain*‐*AOX1TT*‐DoTg1 and UpTg1‐P_G7_‐*kozak*‐*GBD domain*‐*linker*‐*SH3 domain*‐*linker*‐*PDZ domain*‐*AOX1TT*‐DoTg1. The CRISPR/Cas9 plasmid pPIC3.5K‐P*_TEF1_* UP‐gRNA1 was constructed using the above approach.

To replace the native promoter of the *HEM13* gene with the P*_GAP_* promoter, the P*_GAP_* promoter and the upstream and downstream homologous arms of P*_HEM13_* (1 kb) were obtained by PCR using primers pGAP‐H13‐F/pGAP‐H13‐R, UpH13‐F/UpH13‐R, and DoH13‐F/DoH13‐R, respectively, with plasmid pGAPZαA and X33 genomic DNA as templates. These amplified products were fused by overlap extension PCR to generate the knock‐in cassette of the P*_GAP_* promoter, UpH13‐P*_GAP_*‐*kozak*‐DoH13. The downstream homologous arm of P*_HEM13_* contains the *HEM13* gene. The CRISPR/Cas9 plasmid pPIC3.5K‐P*_HEM13_*‐gRNA was generated using the method mentioned above.

To knock out the heme oxygenase gene *HMX1*, the upstream and downstream homologous arms of *HMX1* (1 kb) were amplified using primers UpHMX1‐F/UpHMX1‐R and DoHMX1‐F/DoHMX1‐R, with X33 genomic DNA as the template. These two fragments were fused by overlap extension PCR to generate the gene knock‐out cassette, UpHMX1‐DoHMX1. Subsequently, the CRISPR/Cas9 plasmid pPIC3.5K‐*HMX1*‐gRNA was constructed by replacing the sgRNA sequence in pPIC3.5K‐*ku70*‐gRNA1 with *HMX1*‐gRNA.

### Construction of Engineered *P. Pastoris* Strains

The strains used in this study are listed in Table S6 (Supporting Information). All genetic modifications to *P. pastoris* were made via three ways: i) the monomers with pPICZαA as the backbone, such as pPICZαA‐(P‐Mb)_cn = 1_, pPICZαA‐(S‐Hb)_cn = 1_, pPICZA‐(V‐Hb)_cn = 1_, pPICZA‐(BM3_mut_)_cn = 1_, pPICZA‐*HEM1*, and pPICZA‐*HEM1_53‐561_*, were digested by *Sac* I/or *Pme* I/or *Bst*X I (2‐5 µg DNA after digestion) and transformed into *P. pastoris 5′AOX1* locus by electroporation; the monomers with pGAPZαA as the backbone, including pGAPZA‐*HBSs*‐*linker*‐*mSca*, pGAPZA‐*HEM1*, and pGAPZA‐*HEM1_53‐561_*, were digested by *Avr* II/or *Bsp*H I (2‐5 µg DNA after digestion) and transformed into *P. pastoris 5′GAP* locus by electroporation. ii) the multimers with pPICZαA/or pGAPZαA as the backbone, such as pPICZαA‐(P‐Mb)_cn = 2_, pPICZαA‐(P‐Mb)_cn = 3_, pPICZA‐*GluTR_E_^fbr^*‐*GSAM_E_*, pPICZA‐*GluTR_B_^fbr^*‐*GSAM_B_*, pGAPZA‐*GluTR_E_^fbr^*‐*GSAM_E_*, and pGAPZA‐*GluTR_B_^fbr^*‐*GSAM_B_*, were directly transformed into *P. pastoris 5′AOX1*/or *5′GAP* locus by electroporation without digestion (50‐100 µg DNA). iii) CRISPR/Cas9‐mediated genomic integration.^[^ ^41^ ^]^ About 100 ng CRISPR/Cas9 plasmid and 1 µg donor DNA were co‐transformed into specific integration sites of *P. pastoris*. For the electrotransformation process, please refer to the Invitrogen manual. The transformants were incubated on YPD plates containing 100 µg mL^−1^ Zeocin for 2–3 days at 30 °C for further screening by colony PCR and DNA sequencing. The integrated rounds of expression cassettes based on pPICZA and pGAPZA in *P. pastoris* are verified by colony PCR using the primers listed in Table S5 (Supporting Information), which confirmed the presence of single integrations (Figures S5 and S6 and Table S7, Supporting Information). Positive clones obtained by the third way required the loss of CRISPR/Cas9 plasmids^[^ ^41^ ^]^ by continuous liquid cultivation in the YPD medium without the addition of Zeocin.

### Strains Cultivation

Recombinant *E. coli* DH5α was cultivated in low‐salt Luria‐Bertani medium containing 25 µg mL^−1^ zeocin (5 g L^−1^ yeast extract, 10 g L^−1^ tryptone, 5 g L^−1^ NaCl, pH 7.0) at 37 °C for preservation or extraction of plasmids. *P. pastoris* strains were grown on solid YPD medium (10 g L^−1^ yeast extract, 20 g L^−1^ tryptone, 2% (w/v) glucose, and 20 g L^−1^ agar) at 30 °C for isolating single colonies. For the expression of hemoproteins, a single colony of engineered *P. pastoris* strains was inoculated into 250 mL shake‐flask with 50 mL YPD medium and grown to log phase (OD_600_ = 2–6) at 30 °C with shaking at 250 rpm. Next, the seeding medium was centrifuged at 3000 × g for 5 min, and the supernatant was discarded. Subsequently, the cell pellets were resuspended twice with saline to remove residual sugars. Finally, the treated cells were transferred into 250 mL shake‐flask with 50 mL BMMY medium (1% (v/v) methanol, 10 g L^−1^ yeast extract, 20 g L^−1^ tryptone, 13.4 g L^−1^ YNB, 4 × 10^−5^% (w/v) biotin, 100 mm potassium phosphate buffer, pH 6.0) and cultured at 30 °C with shaking at 250 rpm; methanol was supplemented every 24 h to a final concentration of 1%. To verify the biosynthesis of heme, a single colony of engineered *P. pastoris* strains was inoculated into 50 mL culture tubes containing 5 mL YPD liquid medium and incubated overnight at 30 °C with shaking at 250 rpm. Subsequently, 0.1 mL of the overnight culture was inoculated into 250 mL shake‐flask with 50 mL YPD medium, and fermentation was performed at 30 °C and 250 rpm for 48 h. In studying the transfer of ALA synthesis from the mitochondria to the cytoplasm, the fermentation pattern of pPICZA‐based strains producing ALA was consistent with that of hemoprotein‐expressing strains, whereas pGAPZA‐based strains producing ALA had a fermentation pattern same as that of heme‐synthesizing strains.

### Hemoprotein Quantification

Cell growth was monitored by measuring optical density at 600 nm with a UV‐1280 Spectrophotometer (Shimadzu, Kyoto, Japan). The secreted P‐Mb and S‐Hb in the fermentation supernatant were identified by 10% (w/v) SDS‐PAGE. The titers of P‐Mb and S‐Hb were determined by combining the Bradford method (P0006, Beyotime Biotech, Shanghai, China) and quantitative analysis with Quantity‐One (Bio‐Rad, California, USA). To detect the concentration of intracellularly synthesized V‐Hb and BM3_mut_, cell pellets of 50 mL fermented samples were disrupted using a high‐pressure homogenizer (UH‐06, Union‐Biotech, Shanghai, China), and the corresponding supernatant was purified for analyzing by SDS‐PAGE, Bradford, and Quantity‐One. The purification of secreted or intracellularly synthesized hemoproteins was performed using BeaverBeads^TM^ His‐tag Protein Purification (70 501, Beaver Biomedical Engineering, Suzhou, China). Purified hemoproteins were desalted using Amicon Ultra 3 K (Millipore, Darmstadt, Germany).

### Quantitative Real‐Time PCR (RT‐PCR)

The yeast cells were harvested at the end of the methanol induction period (48 h). Total RNA was extracted from the groups expressing P‐Mb (control) and co‐expressing P‐Mb and Mit1 (test) using Yeast Lytic Enzyme (R1020, Solarbio, Beijing, China) and the RNAprep Pure Plant Kit (DP432, TIANGEN, Beijing, China). After DNase treatment, RNA was reverse transcribed into cDNA using the PrimeScript^TM^ RT reagent Kit with gDNA Eraser (RR047A, TaKaRa, Dalian, China). RT‐PCR was performed using the TB Green^®^ *Premix Ex Taq*™ II (RR820A, TaKaRa, Dalian, China), and reactions were run on a LightCycler 480 II Real‐time PCR instrument (Roche Applied Science, Mannheim, Germany). The procedure was as follows: pre‐denaturation at 95 °C for 30 s, followed by 40 cycles of amplification at 95 °C for 5 s and 60 °C for 30 s. The process concluded with a melt curve stage: 95 °C for 5 s, 60 °C for 60 s, and 95 °C for 1 s. The relative transcription level was calculated using the 2^−ΔΔCt^ method^[^ ^68^ ^]^ with *ARG4* as the reference gene.^[^ ^69^ ^]^ The primers used for RT‐PCR were designed using Beacon Designer 7.9 and were listed in Table S5 (Supporting Information).

### Detection of ALA and Heme

To measure the intracellular concentration of ALA, cells were harvested by centrifugation for 5 min at 12 000 rpm. Cell pellets were washed two times and re‐suspended in phosphate‐buffered saline (PBS) with pH 7.4 for disruption using a FastPrep‐24^TM^ bead‐beating grinder and lysis system (MP Biomedicals, Santa Ana, CA, USA). After removing the cellular debris by centrifugation (12 000 rpm, 5 min), the supernatant was filtered through a 0.22 µm membrane filter and was analyzed by high‐performance liquid chromatography (HPLC, Agilent 1260 series, Agilent Technologies, Waldbronn, Germany) using a C18 column (5 µm, 4.6 mm × 250 mm, Agilent, USA) with a flow rate of 1.0 mL min^−1^, detection wavelength of 338 nm and the column temperature was maintained at 40 °C. The derivatization procedure was performed as follows: 1 µL of the sample was mixed with 7 µL of boric acid, followed by adding and mixing of 2 µL of OPA. Next, 30 µL of ultrapure water was added to the mixture, and finally, 20 µL of the processed sample was injected. The gradient elution of mobile phases A and B proceeded as follows: a ratio of 92% A at 0 min, 40% A at 27.5 min, 0% A during 31.5‐34 min, and 92% A at 35.5 min. Mobile phase A: 3.01 g of anhydrous sodium acetate, 200 µL of triethylamine, 5 mL of tetrahydrofuran, 1 L of ultrapure water, and 5% acetic acid (used to adjust the pH to 7.2). Mobile phase B: 3.01 g of anhydrous sodium acetate, 200 mL of ultrapure water, 5% acetic acid (used to adjust the pH to 7.2), 400 mL of methanol (chromatographic purity), and 400 mL of acetonitrile (chromatographic purity). To detect the intracellular titer of heme, cell pellets were harvested by centrifugation (12 000 rpm, 5 min) and analyzed by the oxalic acid extraction method.^[^ ^34^, ^70^ ^]^

### Analysis by Fluorescence Microscopy

The recombinant strains of HBSs fused with m‐Scarlet were incubated to the log phase (OD_600_ = 2–6). A 10^6^ cells mL^−1^ sample was obtained by diluting cells with PBS buffer (pH 7.4). The cell pellets were collected by centrifugation (12 000 rpm, 5 min) and resuspended in PBS buffer containing 20 nm mitochondrion‐specific dye Mito‐Tracker Green FM (C1048, Beyotime). The stained cells were cultivated at 30 °C in the dark for 30 min and then centrifuged and washed three times with PBS buffer to remove the residual dye. The treated cells were observed using an Eclipse Ci‐L microscope (Nikon, Tokyo, Japan) equipped with a C‐HGFI Intensilight fluorescence illuminator. Fluorescence observation was performed using Mito‐Tracker Green FM (excitation, 490 nm; emission, 516 nm) and m‐Scarlet (excitation, 570 nm; emission, 605 nm) through the oil lens. The above strains observed with white light in the same field of view were used as controls. Micrographs were processed with Image J.

### Determination of the Heme‐Binding Ratio

The UV–vis absorption spectra of purified hemoproteins from 350–700 nm were recorded using a microplate reader (Synergy H1, BioTek Instruments, Winooski, USA). Using the difference spectrum between reduced and oxidized samples to investigate the proper incorporation of heme into hemoprotein.^[^ ^54^ ^]^ The 100 µL of purified hemoprotein was mixed with 100 µL of Solution I (40% (v/v) pyridine, 0.2 m NaOH, and 500 µm potassium ferricyanide) in a 96‐well microtiter plate. Scanning this mixture to obtain the oxidized spectrum. Then, 2 µL of Solution II (0.5 m sodium dithionite in 0.5 m NaOH) was added to the oxidized sample. Scanning the highest peak to generate the reduced spectrum. The heme content was calculated by the Beer‐Lambert Law using the extinction coefficient of pyridine hemochromagen^[^ ^54^ ^]^ of 23.98 mm ^−1^ cm^−1^. The heme‐binding ratio was presented as ([mol of heme]/[mol of globin]) × 100%.

### Assay of Specific Peroxidase Activity for P‐Mb and S‐Hb

The specific peroxidase activity in purified P‐Mb and S‐Hb samples was determined using 3,3′,5,5′‐Tetramethylbenzidine (TMB) Chromogen Solution (P0209, Beyotime).^[^ ^20^ ^]^

### Measurement of Oxygen‐Binding Capacity for V‐Hb

Sodium dithionite (SDT) was used as an oxygen scavenger and reducing reagent for V‐Hb. The purified V‐Hb samples synthesized in the P1 and P1H9 strains were diluted to the same concentration with PBS buffer (pH 7.4). Subsequently, 100 µL of each sample was mixed with 10 µL of SDT solution (10 mg mL^−1^) in a 96‐well microtiter plate. After SDT consumption, deoxy(V‐Hb) was gradually converted to oxy(V‐Hb). The absorbance at 556 nm (*A*_556_) dropped dramatically as oxy(V‐Hb) formed, and the isosbestic point was observed at 523 nm (*A*_523_). The oxygen binding measurement of V‐Hb was reported as the time course of *S*_at.O2_ (oxygen saturation degree).^[^ ^55^ ^]^

(1) $$\begin{array}{ccc} S_{\mathrm{at}.O2} & = & \frac{K_{\mathrm{Hb}}-K_{x}}{K_{\mathrm{Hb}}-K_{\mathrm{HbO}2}},\\ K_{\mathrm{Hb}} & = & \frac{A_{556}}{A_{523t=0}},K_{\mathrm{HbO}2}=\frac{A_{556}}{A_{523t=\infty }},K_{x}=\frac{A_{556}}{A_{523t=t}} \end{array}$$

Where *K*_Hb_ is the absorbance ratio of fully deoxygenated V‐Hb; *K*_HbO2_ is the absorbance ratio of fully oxygenated V‐Hb; *K*_x_ is the absorbance ratio at t = specified time after oxygenation began. The time courses of *A*_556_ and *A*_523_ were recorded using a microplate reader (BioTek Synergy H1).

### Whole‐Cell Catalysis by P450‐BM3_mut_ in Engineered *P. pastoris*

The 2 mL of fermentation broth from the engineered *P. pastoris* strains expressing BM3_mut_ were centrifuged at 6000 rpm for 5 min. The cell pellets were collected and resuspended in 4 mL of potassium phosphate buffer (100 mm, pH 8.0) containing 0.05 g mL^−1^ glucose and 10 mm substrate phenol. The reaction was performed in 50 mL of culture tubes at 30 °C for 8 h with shaking at 250 rpm. Then, 100 µL of the reaction solution was taken and mixed with 900 µL of methanol and centrifuged at 12 000 rpm for 5 min. The supernatant was then isolated and analyzed by HPLC.^[^ ^71^ ^]^ The conversion rate was presented as ([mm of product]/[10 mm of substrate]) × 100%.

### Statistical Analysis

All experiments were independently performed at least three times, and the data were shown as mean values ± standard deviation (SD). Two‐tailed‐Student's t‐test carried out statistical data analysis in GraphPad Prism 8.0. Significance was indicated by NS (non‐significance), *p < 0.05, **p < 0.01, and ***p < 0.001.

## Conflict of Interest

The authors declare no conflict of interest.

## Author Contributions

X.R.Z. and F.Y. designed research. X.R.Z., F.Y., and W.L. performed the experiments and data analysis. J.C., G.C.D., J.H.L., J.W.Z., and X.R.Z. conceived the project and supervised the research. F.Y. and X.R.Z. wrote and revised the manuscript.

## Supporting information

Supporting Information

Supporting Information

## Data Availability

The data that support the findings of this study are available from the corresponding author upon reasonable request.
