# Supplementary material for: Biosynthesis of High‐Active Hemoproteins by the Efficient Heme‐Supply Pichia Pastoris Chassis
Source: Adv Sci (Weinh). 2023 Aug 30;10(30):2302826. doi: 10.1002/advs.202302826 (PMC10602571; doi:10.1002/advs.202302826)
Supplement: Supplementary file 1 — Supporting Information [file ADVS-10-2302826-s002.pdf]

ADVANCED SCIENCE
Open Access

Supporting Information

for *Adv. Sci.*, DOI 10.1002/advs.202302826

Biosynthesis of High-Active Hemoproteins by the Efficient Heme-Supply *Pichia Pastoris* Chassis

Fei Yu, Xinrui Zhao, Jingwen Zhou, Wei Lu, Jianghua Li, Jian Chen and Guocheng Du**

Supporting Information

Biosynthesis of high-active hemoproteins by the efficient heme-supply *Pichia pastoris* chassis

Fei Yu, Xinrui Zhao, Jingwen Zhou, Wei Lu, Jianghua Li, Jian Chen, and Guocheng Du**

F. Yu, X. Zhao, J. Zhou, J. Li, J. Chen, G. Du
Key Laboratory of Industrial Biotechnology, Ministry of Education, School of Biotechnology, Jiangnan University, 1800 Lihu Road, Wuxi, Jiangsu 214122, China
E-mail: zhaoxinrui@jiangnan.edu.cn; gcdu@jiangnan.edu.cn

F. Yu, X. Zhao, J. Zhou, J. Li, J. Chen, G. Du
Science Center for Future Foods, Jiangnan University, 1800 Lihu Road, Wuxi, Jiangsu 214122, China

F. Yu, X. Zhao, J. Zhou, J. Li, J. Chen, G. Du
Jiangsu Province Engineering Research Center of Food Synthetic Biotechnology, Jiangnan University, 1800 Lihu Road, Wuxi, Jiangsu 214122, China

F. Yu, X. Zhao, J. Zhou, J. Li, J. Chen, G. Du
Engineering Research Center of Ministry of Education on Food Synthetic Biotechnology, Jiangnan University, 1800 Lihu Road, Wuxi, Jiangsu 214122, China

W. Lu
Dongsheng Biotech Co., Ltd., 91-92 Junmin Road, Taixing, Jiangsu 225432, China

G. Du
Key Laboratory of Carbohydrate Chemistry and Biotechnology, Ministry of Education, Jiangnan University, 1800 Lihu Road, Wuxi, Jiangsu 214122, China

Contents

Supplementary Figures 1-6

Supplementary Tables 1-7

Supplementary Notes 1-15

Supplementary Figures

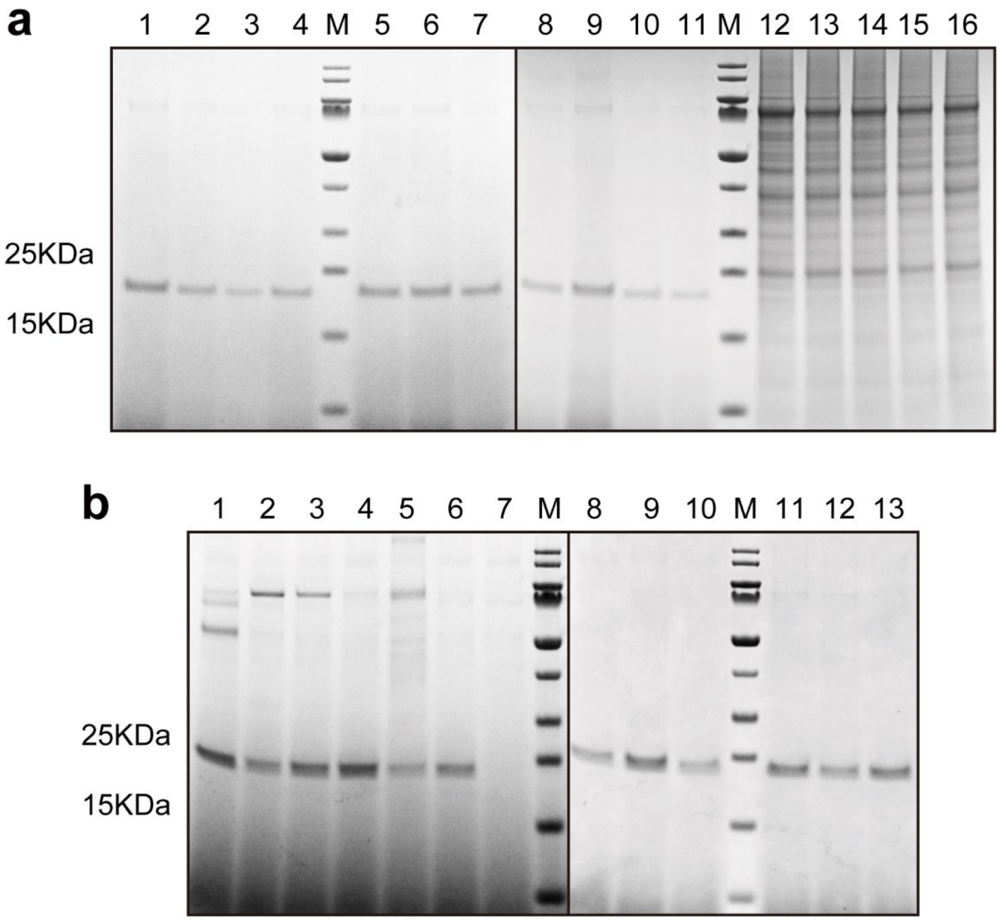

Figure S1. SDS-PAGE analysis of P-Mb titers under different factors and strategies. a) Effect of *P. pastoris* hosts, gene dosage, fermentation time, and α-factor signal peptide (α) on the expression of the globin component in P-Mb. Lane 1-4: the fermentation supernatant of X33-P_{AOX1}-α-$(P\text{-}Mb)_{cn=1}$, KM71-P_{AOX1}-α-$(P\text{-}Mb)_{cn=1}$, SMD1168-P_{AOX1}-α-$(P\text{-}Mb)_{cn=1}$, and GS115-P_{AOX1}-α-$(P\text{-}Mb)_{cn=1}$ at 48 h; Lane 5-7: the fermentation supernatant of X33-P_{AOX1}-α-$(P\text{-}Mb)_{cn=1}$ (single-copy strain), X33-P_{AOX1}-α-$(P\text{-}Mb)_{cn=2}$ (double-copy strain), and X33-P_{AOX1}-α-$(P\text{-}Mb)_{cn=3}$ (three-copy strain) at 48 h; Lane 8-11: the fermentation supernatant of X33-P_{AOX1}-α-$(P\text{-}Mb)_{cn=1}$ at 24, 48, 72, and 96 h; Lane 12-15: the cell lysate of X33-P_{AOX1}-α-$(P\text{-}Mb)_{cn=1}$ at 24, 48, 72, and 96 h; Lane 16: the cell lysate of X33 without the P-Mb gene at 48 h. The molecular weight of P-Mb with 6 × His tags is 17.91 kDa, predicted by ExPASy. M stands for protein ladder. b) Changes in P-Mb titer after overexpressing chaperones and P_{AOX1} transcriptional activators, as well as deleting proteases. Lane 1-7: the fermentation supernatant of X33-*Δku70*-P_{GAP}-*PDI*-(P-Mb), X33-*Δku70*-P_{GAP}-*KAR2*-(P-Mb), X33-*Δku70*-P_{GAP}-*Mxr1*-(P-Mb), X33-*Δku70*-P_{GAP}-*Mit1*-(P-Mb), X33-*Δku70*-P_{GAP}-*Prm1*-(P-Mb), X33-*Δku70*-(P-Mb), and X33-*Δku70* at 48 h; Lane 8-13: the fermentation supernatant of X33-*Δku70*-*Δpep4*-(P-Mb), X33-*Δku70*-*Δyps1*-(P-Mb), X33-*Δku70*-*Δprb1*-(P-Mb), X33-*Δku70*-*Δpep4*-*Δyps1*-(P-Mb), X33-*Δku70*-*Δpep4*-*Δyps1*-*Δprb1*-(P-Mb), and X33-*Δku70*-(P-Mb) at 48 h. cn, copy number.

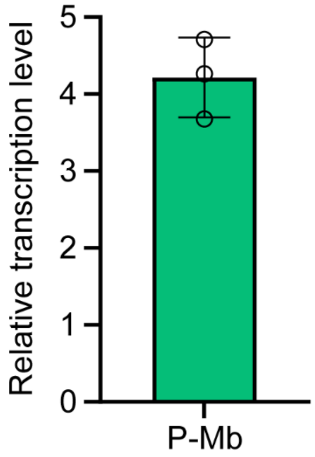

Figure S2. The transcriptional level of P-Mb was quantified using Quantitative Real-Time PCR (RT-PCR) in the X33-*Δku70*-P$_{GAP}$-*Mit1*-(P-Mb) strain at 48 h. The X33-*Δku70*-(P-Mb) strain was used as a control strain. Data presented as mean values ± SD from three independent biological replicates (n = 3).

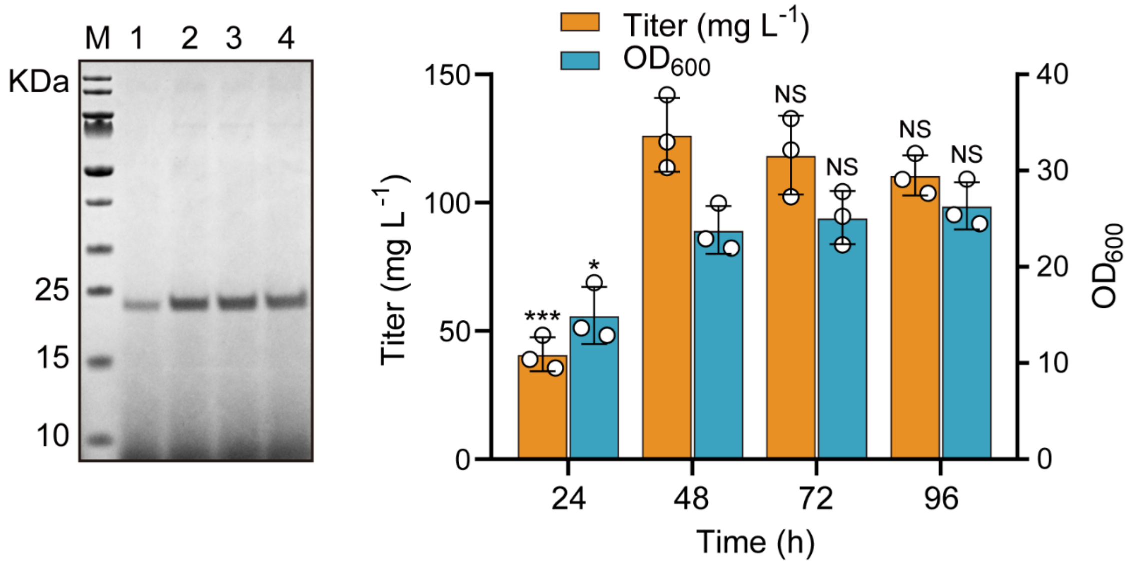

Figure S3. SDS-PAGE analysis of the time-dependent behavior of the P-Mb titer in the fermentation supernatant of the X33-*Δku70*-*Δyps1*-(P-Mb) strain. Lane 1-4: the fermentation supernatant of X33-*Δku70*-*Δyps1*-(P-Mb) at 24, 48, 72, and 96 h. M stands for protein ladder. Data presented as mean values ± SD from three independent biological replicates (n = 3). Statistical evaluation (*p*-value) compared to the control (48 h) was conducted by a two-tailed t-test. *p<0.05, **p<0.01, ***p<0.001 and NS representing non-significance ($p \geq 0.05$).

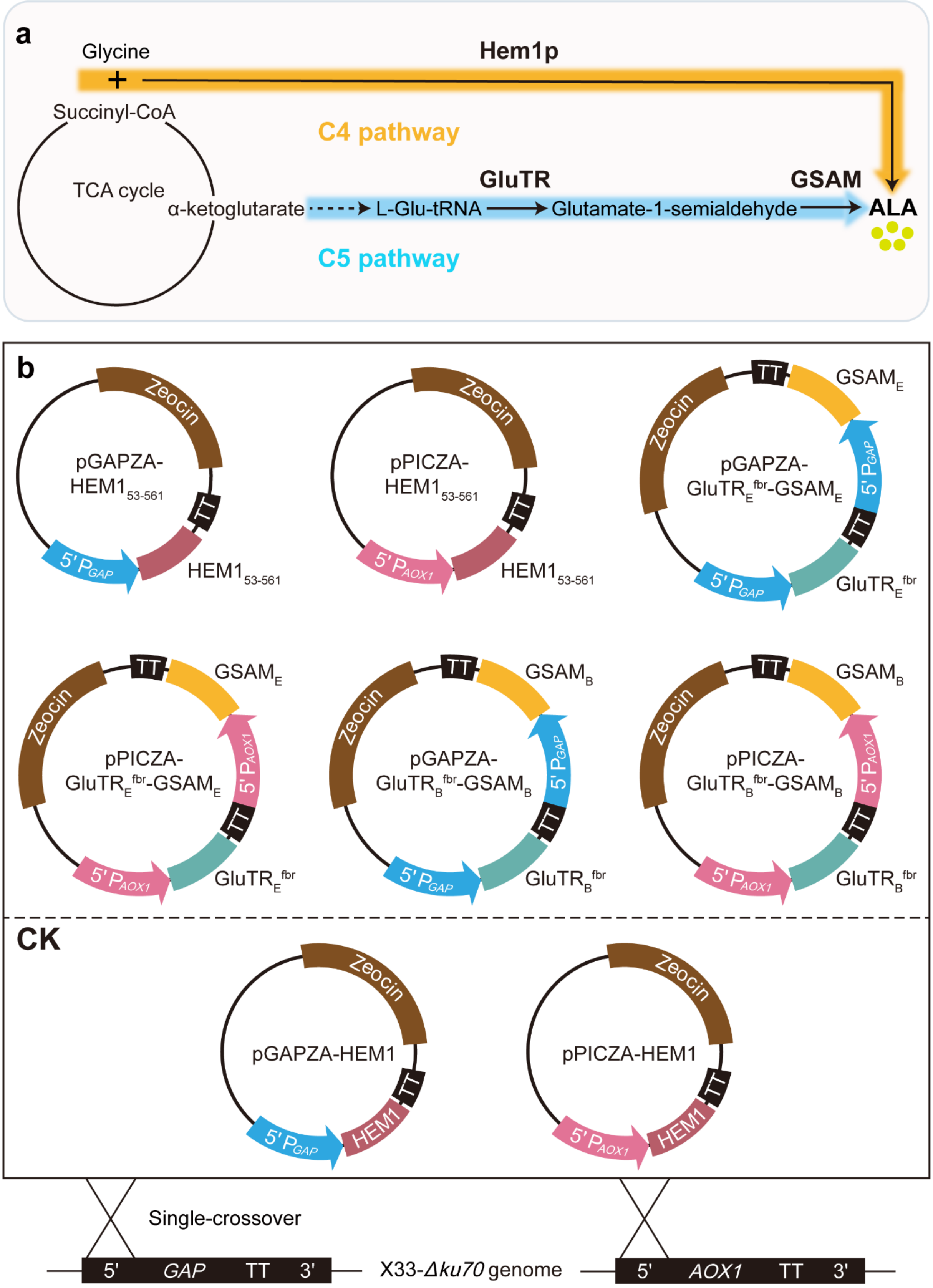

Figure S4. Investigation of the transfer of ALA synthesis from the mitochondria to the cytoplasm in *P. pastoris*. a) The two biosynthetic pathways for ALA found in nature. For the C4 pathway present in *P. pastoris*, Hem1p catalyzes the condensation of glycine and succinyl-CoA to form ALA. For the C5 pathway found in *E. coli* (E) and *B. subtilis* (B), ALA is

synthesized from α-ketoglutarate via a series of enzymatic reactions, including glutamyl-tRNA reductase (GluTR) and glutamate-1-semialdehyde 2,1-aminomutase (GSAM). L-Glu-tRNA, L-glutamate-tRNA. b) Construction of six cytoplasmic ALA-producing strains. These plasmids based on the backbones of pGAPZA or pPICZA were integrated into the *5'GAP* or *5'AOX1* locus of the X33-*Δku70* strain genome through single-crossover events (Invitrogen manual). CK indicates two mitochondrial ALA-producing strains.

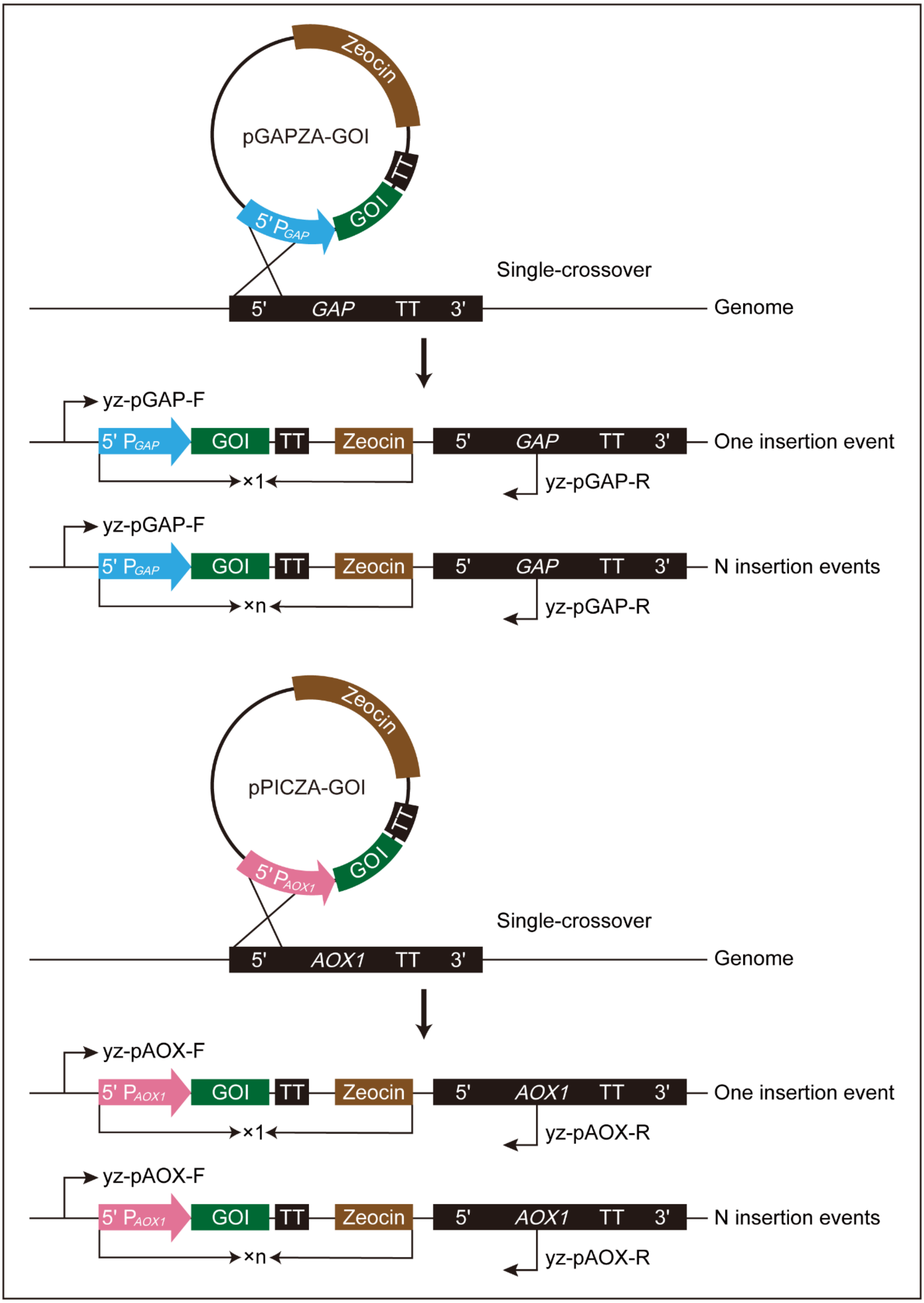

Figure S5. Schematic representation of colony PCR verifies the integration rounds of expression cassettes based on pPICZA and pGAPZA. GOI, gene of interest.

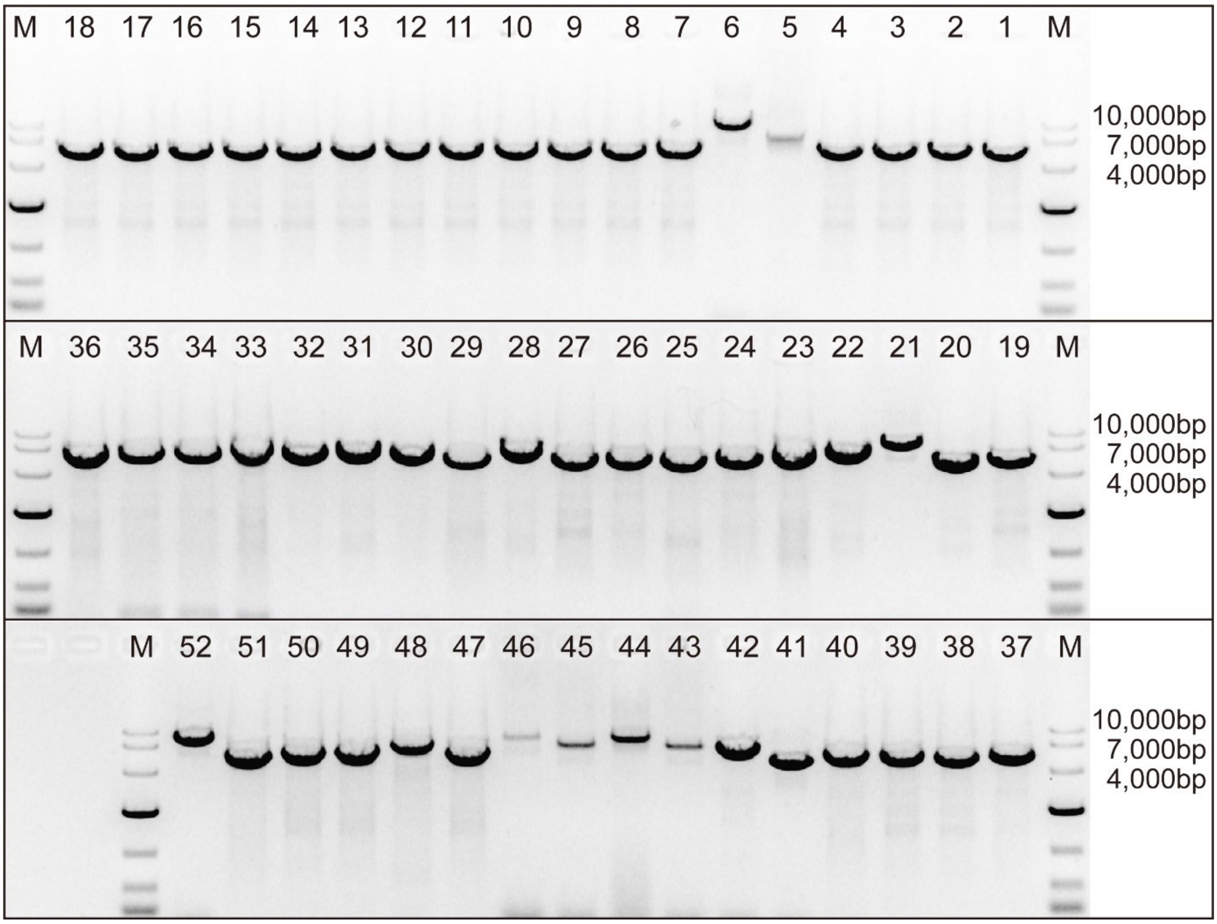

Figure S6. Colony PCR analysis of the integration rounds of expression cassettes based on pPICZA and pGAPZA. Lane 1-10: X33-P_{AOX1}-α-$(P\text{-}Mb)_{cn=1}$, KM71-P_{AOX1}-α-$(P\text{-}Mb)_{cn=1}$, SMD1168-P_{AOX1}-α-$(P\text{-}Mb)_{cn=1}$, GS115-P_{AOX1}-α-$(P\text{-}Mb)_{cn=1}$, X33-P_{AOX1}-α-$(P\text{-}Mb)_{cn=2}$, X33-P_{AOX1}-α-$(P\text{-}Mb)_{cn=3}$, X33-*Δku70*-(P-Mb), X33-*Δku70-Δpep4*-(P-Mb), X33-*Δku70-Δyps1*-(P-Mb), and X33-*Δku70-Δprb1*-(P-Mb); Lane 11-20: X33-*Δku70-Δpep4-Δyps1*-(P-Mb), X33-*Δku70-Δpep4-Δyps1-Δprb1*-(P-Mb), X33-*Δku70*-P_{GAP}-*PDI*-(P-Mb), X33-*Δku70*-P_{GAP}-*KAR2*-(P-Mb), X33-*Δku70*-P_{GAP}-*Mxr1*-(P-Mb), X33-*Δku70*-P_{GAP}-*Mit1*-(P-Mb), X33-*Δku70*-P_{GAP}-*Prm1*-(P-Mb), P1-(P-Mb), P1-(S-Hb), and P1-(V-Hb); Lane 21-30: P1-$BM3_{mut}$, X33-*Δku70*-P_{GAP}-*HEM1-linker-mSca*, X33-*Δku70*-P_{GAP}-*HEM2-linker-mSca*, X33-*Δku70*-P_{GAP}-*HEM3-linker-mSca*, X33-*Δku70*-P_{GAP}-*HEM4-linker-mSca*, X33-*Δku70*-P_{GAP}-*HEM12-linker-mSca*, X33-*Δku70*-P_{GAP}-*HEM13-linker-mSca*, X33-*Δku70*-P_{GAP}-*HEM14-linker-mSca*, X33-*Δku70*-P_{GAP}-*HEM15-linker-mSca*, and X33-*Δku70*-P_{GAP}-$HEM1_{53\text{-}561}$-*linker-mSca*; Lane 31-40: X33-*Δku70*-P_{GAP}-$HEM14_{21\text{-}561}$-*linker-mSca*, X33-*Δku70*-P_{GAP}-$HEM14_{41\text{-}561}$-*linker-mSca*, X33-*Δku70*-P_{GAP}-$HEM14_{61\text{-}561}$-*linker-mSca*, X33-*Δku70*-P_{GAP}-$HEM14_{71\text{-}561}$-*linker-mSca*, X33-*Δku70*-P_{GAP}-$HEM14_{81\text{-}561}$-*linker-mSca*, X33-*Δku70*-P_{GAP}-$HEM15_{21\text{-}375}$-*linker-mSca*, X33-*Δku70*-P_{GAP}-$HEM15_{41\text{-}375}$-*linker-mSca*, X33-*Δku70*-P_{GAP}-$HEM15_{61\text{-}375}$-*linker-mSca*, X33-*Δku70*-P_{GAP}-$HEM15_{71\text{-}375}$-*linker-mSca*, and X33-*Δku70*-P_{GAP}-$HEM15_{81\text{-}375}$-*linker-mSca*; Lane 41-52: X33-*Δku70*-P_{GAP}-$HEM1_{53\text{-}561}$, X33-*Δku70*-P_{AOX1}-$HEM1_{53\text{-}561}$, X33-*Δku70*-P_{GAP}-$GluTR_E^{fbr}$-P_{GAP}-$GSAM_E$, X33-*Δku70*-P_{AOX1}-$GluTR_E^{fbr}$-P_{AOX1}-$GSAM_E$, X33-*Δku70*-P_{GAP}-$GluTR_B^{fbr}$-P_{GAP}-$GSAM_B$, X33-*Δku70*-P_{AOX1}-$GluTR_B^{fbr}$-P_{AOX1}-$GSAM_B$, X33-*Δku70*-P_{GAP}-*HEM1*, X33-*Δku70*-P_{AOX1}-*HEM1*, P1H9-(P-Mb), P1H9-(S-Hb), P1H9-(V-Hb), and P1H9-$BM3_{mut}$. The theoretical amplification length of colony PCR for the single integration of expression cassettes based on pPICZA and pGAPZA was listed in Table S7.

Supplementary Tables

Table S1. Engineering the ALA biosynthesis pathway in the cytoplasm

Strains	Localization	ALA titer (mg L^{-1})
CK-1: P_{GAP}-*HEM1*<br>CK-2: P_{AOX1}-*HEM1*	Mitochondria	100.09 ± 1.29<br>57.70 ± 8.80
P_{GAP}-*HEM1*$_{53\text{-}561}$<br>P_{AOX1}-*HEM1*$_{53\text{-}561}$	Cytoplasm	N.D.
P_{GAP}-$GluTR_E^{fbr}$-P_{GAP}-$GSAM_E$<br>P_{AOX1}-$GluTR_E^{fbr}$-P_{AOX1}-$GSAM_E$<br>P_{GAP}-$GluTR_B^{fbr}$-P_{GAP}-$GSAM_B$<br>P_{AOX1}-$GluTR_B^{fbr}$-P_{AOX1}-$GSAM_B$		

CK stands for the control strain and N.D. represents not detectable. Data presented as mean values ± SD from three independent biological replicates (n = 3).

Table S2. Plasmids, genes, and promoters used in this study

Plasmids/Genes/Promoters	Description	Source
Hemoprotein expression plasmids		
pPICZαA	*P. pastoris* inducible expression plasmid; Zeo^R	Invitrogen
pGAPZαA	*P. pastoris* constitutive expression plasmid; Zeo^R	Invitrogen
pPICZαA-$(P\text{-}Mb)_{cn=1}$	pPICZαA harboring one copy of the *P-Mb* gene; Zeo^R	This study
pPICZαA-$(P\text{-}Mb)_{cn=2}$	pPICZαA harboring two copies of the *P-Mb* gene; Zeo^R	This study
pPICZαA-$(P\text{-}Mb)_{cn=3}$	pPICZαA harboring three copies of the *P-Mb* gene; Zeo^R	This study
pPICZαA-$(S\text{-}Hb)_{cn=1}$	pPICZαA harboring one copy of the *S-Hb* gene; Zeo^R	This study
pPICZA-$(V\text{-}Hb)_{cn=1}$	pPICZA harboring one copy of the *V-Hb* gene; Zeo^R	This study
pPICZA-$(BM3_{mut})_{cn=1}$	pPICZA harboring one copy of the $BM3_{mut}$ gene; Zeo^R	This study
Subcellular localization plasmids of HBS		
pGAPZA-*HEM1-linker-mSca*	pGAPZA carrying a fusion of *HEM1* and *mSca* genes; Zeo^R	This study
pGAPZA-*HEM2-linker-mSca*	pGAPZA carrying a fusion of *HEM2* and *mSca* genes; Zeo^R	This study
pGAPZA-*HEM3-linker-mSca*	pGAPZA carrying a fusion of *HEM3* and *mSca* genes; Zeo^R	This study
pGAPZA-*HEM4-linker-mSca*	pGAPZA carrying a fusion of *HEM4* and *mSca* genes; Zeo^R	This study
pGAPZA-*HEM12-linker-mSca*	pGAPZA carrying a fusion of *HEM12* and *mSca* genes; Zeo^R	This study
pGAPZA-*HEM13-linker-mSca*	pGAPZA carrying a fusion of *HEM13* and *mSca* genes; Zeo^R	This study
pGAPZA-*HEM14-linker-mSca*	pGAPZA carrying a fusion of *HEM14* and *mSca* genes; Zeo^R	This study
pGAPZA-*HEM15-linker-mSca*	pGAPZA carrying a fusion of *HEM15* and *mSca* genes; Zeo^R	This study
pGAPZA-$HEM1_{53\text{-}561}$-*linker-mSca*	pGAPZA carrying a fusion of $HEM1_{53\text{-}561}$ and *mSca* genes; Zeo^R	This study
pGAPZA-$HEM14_{21\text{-}561}$-*linker-mSca*	pGAPZA carrying a fusion of $HEM14_{21\text{-}561}$ and *mSca* genes; Zeo^R	This study
pGAPZA-$HEM14_{41\text{-}561}$-*linker-mSca*	pGAPZA carrying a fusion of $HEM14_{41\text{-}561}$ and *mSca* genes; Zeo^R	This study
pGAPZA-$HEM14_{61\text{-}561}$-*linker-mSca*	pGAPZA carrying a fusion of $HEM14_{61\text{-}561}$ and *mSca* genes; Zeo^R	This study
pGAPZA-$HEM14_{71\text{-}561}$-*linker-mSca*	pGAPZA carrying a fusion of $HEM14_{71\text{-}561}$ and *mSca* genes; Zeo^R	This study
pGAPZA-$HEM14_{81\text{-}561}$-*linker-mSca*	pGAPZA carrying a fusion of $HEM14_{81\text{-}561}$ and *mSca* genes; Zeo^R	This study
pGAPZA-$HEM15_{21\text{-}375}$-*linker-mSca*	pGAPZA carrying a fusion of $HEM15_{21\text{-}375}$ and *mSca* genes; Zeo^R	This study
pGAPZA-$HEM15_{41\text{-}375}$-*linker-mSca*	pGAPZA carrying a fusion of $HEM15_{41\text{-}375}$ and *mSca* genes; Zeo^R	This study
pGAPZA-$HEM15_{61\text{-}375}$-*linker-mSca*	pGAPZA carrying a fusion of $HEM15_{61\text{-}375}$ and *mSca* genes; Zeo^R	This study
pGAPZA-$HEM15_{71\text{-}375}$-*linker-mSca*	pGAPZA carrying a fusion of $HEM15_{71\text{-}375}$ and *mSca* genes; Zeo^R	This study
pGAPZA-$HEM15_{81\text{-}375}$-*linker-mSca*	pGAPZA carrying a fusion of $HEM15_{81\text{-}375}$ and *mSca* genes; Zeo^R	This study

Plasmids for synthesizing ALA		
pGAPZA-*HEM1*$_{53\text{-}561}$	pGAPZA harboring one copy of the *HEM1*$_{53\text{-}561}$ gene; Zeo^R	This study
pPICZA-*HEM1*$_{53\text{-}561}$	pPICZA harboring one copy of the *HEM1*$_{53\text{-}561}$ gene; Zeo^R	This study
pGAPZA-$GluTR_E^{fbr}$-$GSAM_E$	pGAPZA harboring one copy of the $GluTR_E^{fbr}$ and $GSAM_E$ genes; Zeo^R	This study
pPICZA-$GluTR_E^{fbr}$-$GSAM_E$	pPICZA harboring one copy of the $GluTR_E^{fbr}$ and $GSAM_E$ genes; Zeo^R	This study
pGAPZA-$GluTR_B^{fbr}$-$GSAM_B$	pGAPZA harboring one copy of the $GluTR_B^{fbr}$ and $GSAM_B$ genes; Zeo^R	This study
pPICZA-$GluTR_B^{fbr}$-$GSAM_B$	pPICZA harboring one copy of the $GluTR_B^{fbr}$ and $GSAM_B$ genes; Zeo^R	This study
pGAPZA-*HEM1*	pGAPZA harboring one copy of the *HEM1* gene; Zeo^R	This study
pPICZA-*HEM1*	pPICZA harboring one copy of the *HEM1* gene; Zeo^R	This study
CRISPR/Cas9 plasmids		
pPIC3.5K-*ku70*-gRNA1	gRNA-Cas9 all-in-one plasmid containing gRNA targeted *ku70*; Zeo^R; Amp^R	A gift from prof. Menghao Cai[1]
pPIC3.5K-*pep4*-gRNA	Replacement of the gRNA in pPIC3.5K-*Ku70*-gRNA1 with *pep4*-gRNA; Zeo^R; Amp^R	This study
pPIC3.5K-*yps1*-gRNA	Replacement of the gRNA in pPIC3.5K-*Ku70*-gRNA1 with *yps1*-gRNA; Zeo^R; Amp^R	This study
pPIC3.5K-*prb1*-gRNA	Replacement of the gRNA in pPIC3.5K-*Ku70*-gRNA1 with *prb1*-gRNA; Zeo^R; Amp^R	This study
pPIC3.5K-P_{AOX1}UP-gRNA2	Replacement of the gRNA in pPIC3.5K-*Ku70*-gRNA1 with P_{AOX1}UP-gRNA2; Zeo^R; Amp^R	[1]
pPIC3.5K-*AOXTT*DOWN-gRNA	Replacement of the gRNA in pPIC3.5K-*Ku70*-gRNA1 with *AOXTT*DOWN-gRNA; Zeo^R; Amp^R	[1]
pPIC3.5K-*HEM14*-gRNA	Replacement of the gRNA in pPIC3.5K-*Ku70*-gRNA1 with *HEM14*-gRNA; Zeo^R; Amp^R	This study
pPIC3.5K-*HEM15*-gRNA	Replacement of the gRNA in pPIC3.5K-*Ku70*-gRNA1 with *HEM15*-gRNA; Zeo^R; Amp^R	This study
pPIC3.5K-*HEM2*-gRNA	Replacement of the gRNA in pPIC3.5K-*Ku70*-gRNA1 with *HEM2*-gRNA; Zeo^R; Amp^R	This study
pPIC3.5K-*HEM3*-gRNA	Replacement of the gRNA in pPIC3.5K-*Ku70*-gRNA1 with *HEM3*-gRNA; Zeo^R; Amp^R	This study
pPIC3.5K-*HEM4*-gRNA	Replacement of the gRNA in pPIC3.5K-*Ku70*-gRNA1 with *HEM4*-gRNA; Zeo^R; Amp^R	This study
pPIC3.5K-P_{TEF1}UP-gRNA1	Replacement of the gRNA in pPIC3.5K-*Ku70*-gRNA1 with P_{TEF1}UP-gRNA1; Zeo^R; Amp^R	[1]
pPIC3.5K-P_{HEM13}-gRNA	Replacement of the gRNA in pPIC3.5K-*Ku70*-gRNA1 with P_{HEM13}-gRNA; Zeo^R; Amp^R	This study
pPIC3.5K-*HMX1*-gRNA	Replacement of the gRNA in pPIC3.5K-*Ku70*-gRNA1 with *HMX1*-gRNA; Zeo^R; Amp^R	This study
Genes		
P-Mb	Codon optimization of the gene derived from *Sus scrofa* myoglobin (397467) [a]	This study
S-Hb	Codon optimization of the gene derived from *Glycine max* hemoglobin (100527379) [a]	This study
V-Hb	Codon optimization of the gene derived from *Vitreoscilla* hemoglobin (WP_019959060.1) [b]	This study

$BM3_{mut}$	The double mutant A82F/A328F of *Bacillus megaterium* P450-BM3	[2]
ku70	A key gene responsible for the non-homologous end-joining repair mechanism (*PAS_chr3_0329*, 8199462) [a]	This study
pep4	Vacuolar aspartyl proteinase A (*PAS_chr3_1087*, 8200047) [a]	This study
prb1	Vacuolar serine proteinase B (*PAS_chr1-1_0226*, 8196728) [a]	This study
yps1-1	GPI-anchored aspartyl protease (*PAS_chr1-1_0379*, 8196641) [a]	This study
PDI	Protein disulfide isomerase (*PAS_chr4_0844*, 8201243) [a]	This study
KAR2	Endoplasmic reticulum-resident chaperone (*PAS_chr2-1_0140*, 8198455) [a]	This study
Mit1	Methanol-induced transcription factor 1 (*PAS_chr3_0836*, 8200325) [a]	This study
Mxr1	Methanol expression regulator 1 (*PAS_chr4_0487*, 8201433) [a]	This study
Prm1	A positive regulator of genes involved in the methanol utilization pathway (*PAS_chr4_0203*, 8201109) [a]	This study
HEM1	ALA synthase (*PAS_chr2-1_0716*, 8198334) [a]	This study
HEM2	Porphobilinogen synthase (*PAS_chr4_0418*, 8200701) [a]	This study
HEM3	Porphobilinogen deaminase (*PAS_chr1-4_0303*, 8197753) [a]	This study
HEM4	Uroporphyrinogen-III synthase (*PAS_chr2-1_0533*, 8197972) [a]	This study
HEM12	Uroporphyrinogen-III decarboxylase (*PAS_chr3_0954*, 8199701) [a]	This study
HEM13	Coproporphyrinogen-III oxidase (*PAS_chr3_0943*, 8200294) [a]	This study
HEM14	Protoporphyrinogen-IX oxidase (*PAS_chr4_0055*, 8201145) [a]	This study
HEM15	Ferrochelatase (*PAS_chr3_0264*, 8200092) [a]	This study
mScarlet	The codon-optimized gene encoding monomeric red fluorescent protein (QBH90981.1) [b]	This study
$GluTR_E$	Glutamyl-tRNA reductase from *E. coli* BL21(DE3) (ACT43077.1) [b]	This study
$GSAM_E$	Glutamate-1-semialdehyde 2,1-aminomutase from *E. coli* BL21(DE3) (ACT42054.1) [b]	This study
$GluTR_B$	Glutamyl-tRNA reductase from *B. subtilis* 168 (937443) [a]	This study
$GSAM_B$	Glutamate-1-semialdehyde 2,1-aminomutase from *B. subtilis* 168 (937490) [a]	This study
GBD domain	The codon-optimized gene encoding GTPase binding domain from rat actin polymerization switch N-WASP (BAA21534.1, residues from 196 to 274) [b]	[3]
GBD ligand	Interaction ligand of GBD domain (LVGALMHVMQKRSRAIHSSDEGEDQAGDEDED) [c]	[3]
SH3 domain	The codon-optimized gene encoding interaction domain from mouse Crk protein (AAH31149.1, residues from 133 to 190) [b]	[3]
SH3 ligand	Interaction ligand of SH3 domain (PPPALPPKRRR) [c]	[3]

PDZ domain	The codon-optimized gene encoding interaction domain from mouse α-syntrophin (EDL06069.1, residues from 77 to 171) [b]	[3]
PDZ ligand	Interaction ligand of PDZ domain (GVKESLV) [c]	[3]
HMX1	Heme oxygenase (*PAS_chr3_0140*, 8199419) [a]	This study
Promoters		
P_{AOX1}	Powerful methanol-inducible promoter	Invitrogen
P_{GAP}	Moderate constitutive promoter	Invitrogen
P_{G7}	A modified weak constitutive promoter derived from P_{GAP}	[4]

Linker, Gly-Ala-Gly-Ala-Gly-Ala-Gly-Ala-Gly-Ala.
[a] Gene IDs from Genebank.
[b] Protein IDs from GenPept.
[c] Amino acid sequence (5'-3').
Abbreviations: cn, copy number; Zeo^R, Zeocin resistance; HBS, heme biosynthetic enzymes; mSca, monomeric red fluorescent protein (m-Scarlet).

Table S3. Gene knock-in/out cassettes used in this study

Description	Cassettes	Locus
Knockout of *ku70*	Upku70-Doku70 (2 kb)	*ku70*-gRNA1
Knockout of *pep4*	Uppep4-Dopep4 (2 kb)	*pep4*-gRNA
Knockout of *yps1-1*	Upyps1-Doyps1 (2 kb)	*yps1*-gRNA
Knockout of *prb1*	Upprb1-Doprb1 (2 kb)	*prb1*-gRNA
Overexpression of *PDI*	UpAg2-P_{GAP}-*kozak-PDI-AOX1TT*-DoAg2 (4.3 kb)	
Overexpression of *KAR2*	UpAg2-P_{GAP}-*kozak-KAR2-AOX1TT*-DoAg2 (4.8 kb)	
Overexpression of *Mxr1*	UpAg2-P_{GAP}-*kozak-Mxr1-AOX1TT*-DoAg2 (6.2 kb)	P_{AOX1}UP-gRNA2
Overexpression of *Mit1*	UpAg2-P_{GAP}-*kozak-Mit1-AOX1TT*-DoAg2 (5.4 kb)	
Overexpression of *Prm1*	UpAg2-P_{GAP}-*kozak-Prm1-AOX1TT*-DoAg2 (5.7 kb)	
Overexpression of *HEM1*	UpADo-P_{GAP}-*kozak-HEM1*-DoADo (4.2 kb)	*AOXTT*DOWN-gRNA
Removal of MLS_{Hem14p}	UpHEM14-DoHEM14 (2 kb)	*HEM14*-gRNA
Removal of MLS_{Hem15p}	UpHEM15-DoHEM15 (2 kb)	*HEM15*-gRNA
Replacement of *HEM2* with *HEM2-linker-GBD ligand*	UpH2-*HEM2-linker-GBD ligand*-DoH2 (3.1 kb)	*HEM2*-gRNA
Replacement of *HEM3* with *HEM3-linker-SH3 ligand*	UpH3-*HEM3-linker-SH3 ligand*-DoH3 (3.1 kb)	*HEM3*-gRNA
Replacement of *HEM4* with *HEM4-linker-PDZ ligand*	UpH4-*HEM4-linker-PDZ ligand*-DoH4 (2.8 kb)	*HEM4*-gRNA
Introduction of the synthetic protein scaffold driven by P_{GAP}	UpTg1-P_{GAP}-*kozak-GBD domain-linker-SH3 domain-linker-PDZ domain-AOX1TT*-DoTg1 (3.5 kb)	P_{TEF1}UP-gRNA1
Introduction of the synthetic protein scaffold driven by P_{G7}	UpTg1-P_{G7}-*kozak-GBD domain-linker-SH3 domain-linker-PDZ domain-AOX1TT*-DoTg1 (3.5 kb)	
Replacement of the native promoter of *HEM13* with P_{GAP}	UpH13-P_{GAP}-*kozak*-DoH13 (2.5 kb)	P_{HEM13}-gRNA
Knockout of *HMX1*	UpHMX1-DoHMX1 (2 kb)	*HMX1*-gRNA

Kozak, DNA sequence (TTCGAAACG, 5'-3'); Linker, Gly-Ser-Gly-Ser-Gly-Ser-Gly-Ser-Gly.

Table S4. sgRNA sequences used in this study

Target sites	Guide sequence (5'-3') [a]	Source
ku70-gRNA1	CATCTTAGAGAATGTCAGTGAGG	[1]
pep4-gRNA	ACTGGGCAAAGCAATCAATGAGG	This study
yps1-gRNA	GATCAAAATTACCAATGACGAGG	This study
prb1-gRNA	AAACTCACCAGTATCGTCGGAGG	This study
P_{AOX1}UP-gRNA2	GCGCCTACAATGATGACATTTGG	[1]
*AOXTT*DOWN-gRNA	TGACGCTTATTATACCCTTTTGG	[1]
HEM14-gRNA	TGAACCTAAAACGGCAATTGAGG	This study
HEM15-gRNA	ATAGTGTTCATGAATATGGGTGG	This study
HEM2-gRNA	GTACTCACATAGACAGACATCGG	This study
HEM3-gRNA	GATGTGATGTACCACTCCGTCGG	This study
HEM4-gRNA	GCTGGCTTCGTGGATCTACGAGG	This study
P_{TEF1}UP-gRNA1	GCAAGATGGTTAAAAGGTGAAGG	[1]
P_{HEM13}-gRNA	GAATGAAACAACTAAGTGTGGGG	This study
HMX1-gRNA	AGCCACCAAGGGTTACTACGTGG	This study

[a] PAMs are underlined.

Table S5. Primers used in this study

Name (-F/-R) [a]	Sequence (5'-3')	Notes [b]
Upku70-F	ACTTTAATGGGGTAAAATATCAGCAGTAGGCTGA	Construction of Upku70-Doku70
Upku70-R	GAGGAAAAAGTAAGGAACACGAACTTGCAATG CTTTTTATTATTCTCTGTGTTAGGGC	
Doku70-F	AGAATAATAAAAAGCATTGCAAGTTCGTGTTCCT TACTTTTTCCTCGCAACGT	
Doku70-R	AAATGGATTCCGAGAGACTAGTTACGTTCTGAT	
Uppep4-F	AGAAGCGAGTTTCTCCGTATCTAATCT	Construction of Uppep4-Dopep4
Uppep4-R	TCAGCTGAGCAACTTTTATTCTTGCCTTTAAATTG ATTATCAGTAATTGAGAAATGGAACTCT	
Dopep4-F	TCAATTACTGATAATCAATTTAAAGGCAAGAATA AAAGTTGCTCAGCTGAACT	
Dopep4-R	TCATCTATACCCCAGGACCAGGT	
Upyps1-F	TCTCTCGTAGAAATTTTGTCAATACATTACTCCCA TATGAGTGCCGGA	Construction of Upyps1-Doyps1
Upyps1-R	AAAGTCATAAACGCGTATTTCGGTAAGGCACTTG ATCAAGTTGGACGAGT	
Doyps1-F	AACTTGATCAAGTGCCTTACCGAAATACGCGTTT ATGACTTTCAATTGACTTGCAACTATTAGATTTCA TGCGT	
Doyps1-R	ATTTGTGGTATCTGGAGTATTGTCCTTGAGAACG TCTTTGGT	
Upprb1-F	AATAACTTCATGACTGCATTTGTTCAATTGAACT GGTGTTTGAACAACGCCT	Construction of Upprb1-Doprb1
Upprb1-R	ACTCCTTGTGTATTCCTTTTCTCCAATAGTTTAAA TAATATGGAGATTTGTTTGAAGAGAGTTAAAAGG GACAGTAAAACAACACTAGA	
Doprb1-F	TTTTAACTCTCTTCAAACAAATCTCCATATTATTT AAACTATTGGAGAAAAGGAATACACAAGGAGTT AAAAAAAGTGTGGTAGAAAGT	
Doprb1-R	AGTATCCTGCTCATCTTCCCGTACAGCTTTAATTG CCT	
UpAg2-F	CGTTTCGAATAATTAGTTGTTTTTGATCTTCTCA AGTTGTCGTT	Construction of UpAg2-P_{GAP}-*kozak-PDI-AOX1TT*-DoAg2
UpAg2-R	ACACCAAGACATTTCTACAAAAAGTTGGTATTGT GAAATAGACGCAGATCGGGA	
pGAP-Ag2-F	TGCGTCTATTTCACAATACCAACTTTTTGTAGAA ATGTCTTGGTGTCCTCGTCCA	
pGAP-PDI-R	ATTCCAGTTGAATTGCATCGTTTCGAAATAGTTG TTCAATTGATTGAAATAGGGACAAATAAATTAAA TTTAAAGTCTTTGGGT	
PDI-F	TGAACAACTATTTCGAAACGATGCAATTCAACTG GAATATTAAAACTGTGGCAAGT	
PDI-R	GGCATTCTGACATCCTCTTGATTAAAGCTCGTCG TGAGCGTCTGCCTCA	
AOXTT-PDI-F	CTCACGACGAGCTTTAATCAAGAGGATGTCAGA ATGCCATTTGCCTGAGAGAT	
AOXTT-Ag2-R	AGCAATTAGGAGAAAACTATTAATCTACATTCTC ACTTAATCTTCTGTACTCTGAAGAGGAGTGGGA	
DoAg2-F	TCTTCAGAGTACAGAAGATTAAGTGAGAATGTA GATTAATAGTTTTCTCCTAATTGCTCTAAAACCCT AGCCCT	

DoAg2-R	GATTTATCGGATGAAGGCTACTATATCAAAGACTTAGCACTAAGGA	
UpAg2-F	CGTTTCGAATAATTAGTTGTTTTTTGATCTTCTCAAGTTGTCGTT	Construction of UpAg2-P$_{GAP}$-*kozak-KAR2-AOX1TT*-DoAg2
UpAg2-R	ACACCAAGACATTTCTACAAAAAGTTGGTATTGTGAAATAGACGCAGATCGGGA	
pGAP-Ag2-F	TGCGTCTATTTCACAATACCAACTTTTTGTAGAAATGTCTTGGTGTCCTCGTCCA	
pGAP-KAR2-R	CAAGATGGTTTTAACGACAGCATCGTTTCGAAATAGTTGTTCAATTGATTGAAATAGGGACAAATAAATTAAATTTAAAGTCTTTGGGT	
KAR2-F	TGAACAACTATTTCGAAACGATGCTGTCGTTAAAACCATCTTGGCTGACT	
KAR2-R	GGCATTCTGACATCCTCTTGACTACAACTCATCATGATCATAGTCATAGTCGTAGTCAAAGTCT	
AOXTT-KAR2-F	CATGATGAGTTGTAGTCAAGAGGATGTCAGAATGCCATTTGCCTGAGAGAT	
AOXTT-Ag2-R	AGCAATTAGGAGAAAACTATTAATCTACATTCTCACTTAATCTTCTGTACTCTGAAGAGGAGTGGGA	
DoAg2-F	TCTTCAGAGTACAGAAGATTAAGTGAGAATGTAGATTAATAGTTTTCTCCTAATTGCTCTAAAACCCTAGCCCT	
DoAg2-R	GATTTATCGGATGAAGGCTACTATATCAAAGACTTAGCACTAAGGA	
UpAg2-F	CGTTTCGAATAATTAGTTGTTTTTTGATCTTCTCAAGTTGTCGTT	Construction of UpAg2-P$_{GAP}$-*kozak-Mit1-AOX1TT*-DoAg2
UpAg2-R	ACACCAAGACATTTCTACAAAAAGTTGGTATTGTGAAATAGACGCAGATCGGGA	
pGAP-Ag2-F	TGCGTCTATTTCACAATACCAACTTTTTGTAGAAATGTCTTGGTGTCCTCGTCCA	
pGAP-Mit1-R	GCTGCGGTACTCATCGTTTCGAAATAGTTGTTCAATTGATTGAAATAGGGACAAATAAATTAAATTTAAAGTCTTTGGGT	
Mit1-F	TGAACAACTATTTCGAAACGATGAGTACCGCAGCCCCAATCAAGGA	
Mit1-R	GGCATTCTGACATCCTCTTGACTATTCTTCAACATTCCAGTAGTCAATTAACTCCTTGCCCT	
AOXTT-Mit1-F	GGAATGTTGAAGAATAGTCAAGAGGATGTCAGAATGCCATTTGCCTGAGAGAT	
AOXTT-Ag2-R	AGCAATTAGGAGAAAACTATTAATCTACATTCTCACTTAATCTTCTGTACTCTGAAGAGGAGTGGGA	
DoAg2-F	TCTTCAGAGTACAGAAGATTAAGTGAGAATGTAGATTAATAGTTTTCTCCTAATTGCTCTAAAACCCTAGCCCT	
DoAg2-R	GATTTATCGGATGAAGGCTACTATATCAAAGACTTAGCACTAAGGA	
UpAg2-F	CGTTTCGAATAATTAGTTGTTTTTTGATCTTCTCAAGTTGTCGTT	Construction of UpAg2-P$_{GAP}$-*kozak-Mxr1-AOX1TT*-DoAg2
UpAg2-R	ACACCAAGACATTTCTACAAAAAGTTGGTATTGTGAAATAGACGCAGATCGGGA	
pGAP-Ag2-F	TGCGTCTATTTCACAATACCAACTTTTTGTAGAAATGTCTTGGTGTCCTCGTCCA	
pGAP-Mxr1-R	GGGGGTAGATTGCTCATCGTTTCGAAATAGTTGTTCAATTGATTGAAATAGGGACAAATAAATTAAATTTAAAGTCTTTGGGT	

Mxr1-F	TGAACAACTATTTCGAAACGATGAGCAATCTACCCCCAACTTTTGGTTCCA	
Mxr1-R	GGCATTCTGACATCCTCTTGACTAGACACCACCATCTAGTCGGTTTTCTAGGT	
AOXTT-Mxr1-F	TGGTGGTGTCTAGTCAAGAGGATGTCAGAATGCCATTTGCCTGAGAGAT	
AOXTT-Ag2-R	AGCAATTAGGAGAAAACTATTAATCTACATTCTCACTTAATCTTCTGTACTCTGAAGAGGAGTGGGA	
DoAg2-F	TCTTCAGAGTACAGAAGATTAAGTGAGAATGTAGATTAATAGTTTTCTCCTAATTGCTCTAAAACCCTAGCCCT	
DoAg2-R	GATTTATCGGATGAAGGCTACTATATCAAAGACTTAGCACTAAGGA	
UpAg2-F	CGTTTCGAATAATTAGTTGTTTTTGATCTTCTCAAGTTGTCGTT	
UpAg2-R	ACACCAAGACATTTCTACAAAAAGTTGGTATTGTGAAATAGACGCAGATCGGGA	
pGAP-Ag2-F	TGCGTCTATTTCACAATACCAACTTTTTGTAGAAATGTCTTGGTGTCCTCGTCCA	
pGAP-Prm1-R	CCGATGTTTAGGAGGCATCGTTTCGAAATAGTTGTTCAATTGATTGAAATAGGGACAAATAAATTAAATTTAAAGTCTTTGGGT	
Prm1-F	TGAACAACTATTTCGAAACGATGCCTCCTAAACATCGGCTGGAGCA	Construction of UpAg2-P$_{GAP}$-*kozak-Prm1-AOX1TT*-DoAg2
Prm1-R	GGCATTCTGACATCCTCTTGATTAACTGTCAAAATTTATTGTATCTGGCGCAGTGGTCCA	
AOXTT-Prm1-F	CCAGATACAATAAATTTTGACAGTTAATCAAGAGGATGTCAGAATGCCATTTGCCTGAGAGAT	
AOXTT-Ag2-R	AGCAATTAGGAGAAAACTATTAATCTACATTCTCACTTAATCTTCTGTACTCTGAAGAGGAGTGGGA	
DoAg2-F	TCTTCAGAGTACAGAAGATTAAGTGAGAATGTAGATTAATAGTTTTCTCCTAATTGCTCTAAAACCCTAGCCCT	
DoAg2-R	GATTTATCGGATGAAGGCTACTATATCAAAGACTTAGCACTAAGGA	
UpADo-F	TGATTCATACTGCTTCATTGTTGCATGATGATGTTATTGATTTTTCTGACT	
UpADo-R	AGACATTTCTACAAAAACAACTTGAGGGGCACTATCTTGTTTTAGAGAAATTTGCGGA	
pGAP-ADo-F	ACAAGATAGTGCCCCTCAAGTTGTTTTTGTAGAAATGTCTTGGTGTCCTCGTCCAATCAGGTAG	
pGAP-HEM1-R	GGCGACAAACTCCATCGTTTCGAAATAGTTGTTCAATTGATTGAAATAGGGACAAATAAATTAAATTTAAAGTCTTTGGGT	Construction of UpADo-P$_{GAP}$-*kozak-HEM1*-DoADo
HEM1-F	TGAACAACTATTTCGAAACGATGGAGTTTGTCGCCCGTCAGT	
HEM1-R	TGTAACCTATATAGTATAGGATTTTTTTTGCTACAATCTGACTCCTGATGAGGTTTCGATCTGCTCAA	
DoADo-F	ACCTCATCAGGAGTCAGATTGTAGCAAAAAAAATCCTATACTATATAGGTTACAAATAAAAAAGTAATCAAAAATGAAGCCTGCAT	
DoADo-R	ACCAACACCAGAAGAACTCTCTCAAATGGACGAAT	
UpH14-F	TTTACAGTCAGTCAAAATAAGAGTAGAGAATATTATTAGACTAAAAAGTACGTTTACCAAACGT	

UpH14-R	ACAACATCAAGGTACCCGTCATCTATTGTACCACTAGCTCTCAGCACCCA	Construction of UpHEM14-DoHEM14
DoH14-F	TGGTACAATAGATGACGGGTACCTTGATGTTGTTGGATCTTCT	
DoH14-R	TCTCGATGTCTGAGTCAAATATGACACCGAGAAGT	
UpH15-F	ATATAAGCATTTAAGATTTGAACTTTTTGGGGTATTATTACACTCCTTGAAACT	Construction of UpHEM15-DoHEM15
UpH15-R	CTCCGATAGCTTTGTAGTAGGACATCTTGTAAGTTGTCTAGAAGGAAGGAAATAATTTTGCGGG	
DoH15-F	AGACAACTTACAAGATGTCCTACTACAAAGCTATCGGAGGTGGGTCT	
DoH15-R	GCGGAAGGGTTCAAAGTAGTACAGGCTAGGT	
UpH2-F	GACTGCTGCCCTTTTGGTACCCCGTTT	Construction of UpH2-*HEM2-linker-GBD ligand*-DoH2
UpH2-R	AAGTATTCAGCCTTATGCACCATTTTGAAAATAGTTTAGATGAAGGTGAGTTTGCCTAGTGAGGAAGCA	
H2-F	CTCACCTTCATCTAAACTATTTCAAAATGGTGCATAAGGCTGAATACTTGGACGACCA	
H2LG-R1	TCAAAGCACCAACCAAACCAGAACCAGAACCAGAACCAGAACCTTCAGATAACCACTCCAGGAATTCAGGGGTAAAGTAACTAATGAT	
H2LG-R2	ATCTTCACCTTCATCAGAAGAATGAATAGCTCTAGATCTCTTTTGCATAACATGCATCAAAGCACCAACCAAACCAGAACCAGAACCA	
H2LG-R3	AGTCGATGAACGATTAATCTTCATCTTCATCACCAGCTTGATCTTCACCTTCATCAGAAGAATGAATAGCTCTAGATCTCTTTTGCAT	
DoH2-F	AGCTGGTGATGAAGATGAAGATTAATCGTTCATCGACTCTGTATTACTTGTACATATTCATATACACCGATTGT	
DoH2-R	TGCTACGGTGCCTGATGTCACTTGGAAAA	
UpH3-F	CGGATTTTCCCCTGTTCGGTCAGAATCGGT	Construction of UpH3-*HEM3-linker-SH3 ligand*-DoH3
UpH3-R	GCTCTGTTCGATTTGGTTCATGTTCTGATAGCTCTCTAGCATAGCTGAGTGGGCT	
H3-F	TATGCTAGAGAGCTATCAGAACATGAACCAAATCGAACAGAGCGGACCCA	
H3LS-R1	AAAGCTGGTGGTGGACCAGAACCAGAACCAGAACCAGAACCATGTAAACCCTCTTCCTTAATCTCTTTGATCTTGTTGAAGTTGATCT	
H3LS-R2	CGTTTTTCTATATAAATGGTAGTCATTATCTTCTTCTCTTTGGTGGCAAAGCTGGTGGTGGACCAGAACCAGAACCA	
DoH3-F	AGCTTTGCCACCAAAGAGAAGAAGATAATGACTACCATTTATATAGAAAAACGCCTAATTGTATAATAGAAGAAGATTCTCA	
DoH3-R	TCTAGCAGCACTAACAATGTAAACAGGTTCACTCATGATT	
UpH4-F	GTTCTCCGTTGATTCATTCTTGTGAGAAGCGGA	Construction of UpH4-*HEM4-linker-PDZ ligand*-DoH4
UpH4-R	AGAATGGCTTTTGGCATGATATGGGACAACAAGAAGACGGTACTATTAACTACGGTCAAAGT	
H4-F	TCTTCTTGTTGTCCCATATCATGCCAAAAGCCATTCTTCTGAAGAATAAAACTACACCGA	

H4LP-R	AAAGATTCCTTAACACCACCAGAACCAGAACCAGAACCAGAACCGTGCACTTTTTGTATAGACAATAGTAAAGAGATAGGCTCTGGCT	
DoH4-F	GTTCTGGTTCTGGTTCTGGTGGTGTTAAGGAATCTTTGGTTTAAAGACAAAATGAGCCTTACTTCAAAATGAGACTGTTGTTGCGT	
DoH4-R	GCAACTTTCAGAATAACCACAGGTTATATTCCAGATTCTATACTTGCAAGT	
UpTg1-F	CCCTGAATGCGTGCTTAATAGTGGACTTTCTTAGGAGA	Construction of UpTg1-P_{GAP}-*kozak-GBD domain-linker-SH3 domain-linker-PDZ domain-AOX1TT*-DoTg1
UpTg1-R	AGACATTTCTACAAAAAGGTTGCCAGAGTTACGTAACCAGATGCTGAGA	
pGAP_G7-Tg1-F	CTGGTTACGTAACTCTGGCAACCTTTTTGTAGAAATGTCTTGGTGTCCTCGTCCAATCAGGT	
pGAP-GSP-R	GCCTTAGTCATCGTTTCGAAATAGTTGTTCAATTGATTGAAATAGGGACAAATAAATTAAATTTAAAGTCTTTGGGTCAGGA	
GSP-F	CCCTATTTCAATCAATTGAACAACTATTTCGAAACGATGACTAAGGCTGATATTGGTACTCCATCTAACTTTCAACATATTGGTCAT	
GSP-R	GGCATTCTGACATCCTCTTGATTACTTAAAGTATGGAGAAACTTCCTTCATGTACTTAACTTCCAAAACAACTTCCT	
AOXTT-GSP-F	GGAAGTTTCTCCATACTTTAAGTAATCAAGAGGATGTCAGAATGCCATTTGCCTGAGAGAT	
AOXTT-Tg1-R	AAGGGGACACCTCATGCATCTCACTTAATCTTCTGTACTCTGAAGAGGAGTGGGAAATACCA	
DoTg1-F	ACAGAAGATTAAGTGAGATGCATGAGGTGTCCCCTTAGTGGGAAAGAGTACT	
DoTg1-R	TGGCTCAATCATGTTGTCACCGTTCCATCCGGA	
UpTg1-F	CCCTGAATGCGTGCTTAATAGTGGACTTTCTTAGGAGA	Construction of UpTg1-P_{G7}-*kozak-GBD domain-linker-SH3 domain-linker-PDZ domain-AOX1TT*-DoTg1
UpTg1-R	AGACATTTCTACAAAAAGGTTGCCAGAGTTACGTAACCAGATGCTGAGA	
pGAP_G7-Tg1-F	CTGGTTACGTAACTCTGGCAACCTTTTTGTAGAAATGTCTTGGTGTCCTCGTCCAATCAGGT	
pG7-GSP-R	GCCTTAGTCATCGTTTCGAAATAGTTGTTCAATTGATTGAAATAGGGACAAATAAATTAATTTTAAAGTCTTTGGGTCAGGA	
GSP-F	CCCTATTTCAATCAATTGAACAACTATTTCGAAACGATGACTAAGGCTGATATTGGTACTCCATCTAACTTTCAACATATTGGTCAT	
GSP-R	GGCATTCTGACATCCTCTTGATTACTTAAAGTATGGAGAAACTTCCTTCATGTACTTAACTTCCAAAACAACTTCCT	
AOXTT-GSP-F	GGAAGTTTCTCCATACTTTAAGTAATCAAGAGGATGTCAGAATGCCATTTGCCTGAGAGAT	
AOXTT-Tg1-R	AAGGGGACACCTCATGCATCTCACTTAATCTTCTGTACTCTGAAGAGGAGTGGGAAATACCA	
DoTg1-F	ACAGAAGATTAAGTGAGATGCATGAGGTGTCCCCTTAGTGGGAAAGAGTACT	
DoTg1-R	TGGCTCAATCATGTTGTCACCGTTCCATCCGGA	
UpH13-F	TTTAATTACTAATTTGCTCCTATATCATGAACTCTGGAATTCTACGTCAT	

UpH13-R	AGGACACCAAGACATTTCTACAAAAACTATTTACTTCTCAATCTAGATCTCTTTCGTTTCGACTTGGTATTAACA	Construction of UpH13-P*GAP*-*kozak*-DoH13
pGAP-H13-F	AGATCTAGATTGAGAAGTAAATAGTTTTTGTAGAAATGTCTTGGTGTCCTCGTCCAATCAGGT	
pGAP-H13-R	AGTCGATGGCCATCGTTTCGAAATAGTTGTTCAATTGATTGAAATAGGGACAAATAAATTAAATTTAAAGTCTTTGGGTCAGGA	
DoH13-F	CAATCAATTGAACAACTATTTCGAAACGATGGCCATCGACTCTGATATCAATCTAAGCTCTCCCA	
DoH13-R	CATATCAACCTTTAAATACTCATCATACCCATTCAATAGGATTTTGTAGTACCT	
UpHMX1-F	ATACAACGAAGATGTCAGTGATGCACCGCCAGAT	Construction of UpHMX1-DoHMX1
UpHMX1-R	ACATAGGAAATAAATAAATCAGCATCAATGTCATCGTGGGTCAGGTAAAACTGGTAGCAGCTTT	
DoHMX1-F	AGTTTTACCTGACCCACGATGACATTGATGCTGATTTATTTATTTCCTATGTATATTAGTATTCAATCATATTGCATCATCGCTT	
DoHMX1-R	ATGTTGTTTCTATAGACCGCTCAAGACCACTGATGCCATTAATAT	
pep4-gRNA-F1	TGAGGACGAAACGAGTAAGCTCGTCACTGGGCAAAGCAATCAATGGTTTTAGAGCTAGAAATAGCAAGTTAAAATAAGGCTAGTCCGTT	Construction of pPIC3.5K-*pep4*-gRNA
Cas9-backbone-R1	TGTTTGCCGGATCAAGAGCTACCAACTCT	
Cas9-backbone-F2	AGAGTTGGTAGCTCTTGATCCGGCAAACA	
pep4-gRNA-R2	CGAGCTTACTCGTTTCGTCCTCACGGACTCATCAGACTGGGTTTGATTTGTTTAGGTAACTTGAACTGGATGTATTAGTTTGGTGACAA	
yps1-gRNA-F1	TGAGGACGAAACGAGTAAGCTCGTCGATCAAAATTACCAATGACGGTTTTAGAGCTAGAAATAGCAAGTTAAAATAAGGCTAGTCCGTT	Construction of pPIC3.5K-*yps1*-gRNA
Cas9-backbone-R1	TGTTTGCCGGATCAAGAGCTACCAACTCT	
Cas9-backbone-F2	AGAGTTGGTAGCTCTTGATCCGGCAAACA	
yps1-gRNA-R2	CGAGCTTACTCGTTTCGTCCTCACGGACTCATCAGGATCAATTTGATTTGTTTAGGTAACTTGAACTGGATGTATTAGTTTGGTGACAA	
prb1-gRNA-F1	TGAGGACGAAACGAGTAAGCTCGTCAAACTCACCAGTATCGTCGGGTTTTAGAGCTAGAAATAGCAAGTTAAAATAAGGCTAGTCCGTT	Construction of pPIC3.5K-*prb1*-gRNA
Cas9-backbone-R1	TGTTTGCCGGATCAAGAGCTACCAACTCT	
Cas9-backbone-F2	AGAGTTGGTAGCTCTTGATCCGGCAAACA	
prb1-gRNA-R2	CGAGCTTACTCGTTTCGTCCTCACGGACTCATCAGAAACTCTTTGATTTGTTTAGGTAACTTGAACTGGATGTATTAGTTTGGTGACAA	
pAOX1UP-gRNA2-F1	TGAGGACGAAACGAGTAAGCTCGTCGCGCCTACAATGATGACATTGTTTTAGAGCTAGAAATAGCAAGTTAAAATAAGGCTAGTCCGTT	Construction of pPIC3.5K-P*AOX1*UP-gRNA2
Cas9-backbone-R1	TGTTTGCCGGATCAAGAGCTACCAACTCT	
Cas9-backbone-F2	AGAGTTGGTAGCTCTTGATCCGGCAAACA	
pAOX1UP-gRNA2-R2	CGAGCTTACTCGTTTCGTCCTCACGGACTCATCAGGCGCCTTTTGATTTGTTTAGGTAACTTGAACTGGATGTATTAGTTTGGTGACAA	

AOXTTDOWN-gRNA-F1	TGAGGACGAAACGAGTAAGCTCGTCTGACGCTTATTATACCCTTTGTTTTAGAGCTAGAAATAGCAAGTTAAAATAAGGCTAGTCCGTT	Construction of pPIC3.5K-*AOXTT*DOWN-gRNA
Cas9-backbone-R1	TGTTTGCCGGATCAAGAGCTACCAACTCT	
Cas9-backbone-F2	AGAGTTGGTAGCTCTTGATCCGGCAAACA	
AOXTTDOWN-gRNA-R2	CGAGCTTACTCGTTTCGTCCTCACGGACTCATCAGTGACGCTTTGATTTGTTTAGGTAACTTGAACTGGATGTATTAGTTTGGTGACAA	
HEM14-gRNA-F1	TGAGGACGAAACGAGTAAGCTCGTCTGAACCTAAAACGGCAATTGGTTTTAGAGCTAGAAATAGCAAGTTAAAATAAGGCTAGTCCGTT	Construction of pPIC3.5K-*HEM14*-gRNA
Cas9-backbone-R1	TGTTTGCCGGATCAAGAGCTACCAACTCT	
Cas9-backbone-F2	AGAGTTGGTAGCTCTTGATCCGGCAAACA	
HEM14-gRNA-R2	CGAGCTTACTCGTTTCGTCCTCACGGACTCATCAGTGAACCTTTGATTTGTTTAGGTAACTTGAACTGGATGTATTAGTTTGGTGACAA	
HEM15-gRNA-F1	TGAGGACGAAACGAGTAAGCTCGTCATAGTGTTCATGAATATGGGGTTTTAGAGCTAGAAATAGCAAGTTAAAATAAGGCTAGTCCGTT	Construction of pPIC3.5K-*HEM15*-gRNA
Cas9-backbone-R1	TGTTTGCCGGATCAAGAGCTACCAACTCT	
Cas9-backbone-F2	AGAGTTGGTAGCTCTTGATCCGGCAAACA	
HEM15-gRNA-R2	CGAGCTTACTCGTTTCGTCCTCACGGACTCATCAGATAGTGTTTGATTTGTTTAGGTAACTTGAACTGGATGTATTAGTTTGGTGACAA	
HEM2-gRNA-F1	TGAGGACGAAACGAGTAAGCTCGTCGTACTCACATAGACAGACATGTTTTAGAGCTAGAAATAGCAAGTTAAAATAAGGCTAGTCCGTT	Construction of pPIC3.5K-*HEM2*-gRNA
Cas9-backbone-R1	TGTTTGCCGGATCAAGAGCTACCAACTCT	
Cas9-backbone-F2	AGAGTTGGTAGCTCTTGATCCGGCAAACA	
HEM2-gRNA-R2	CGAGCTTACTCGTTTCGTCCTCACGGACTCATCAGGTACTCTTTGATTTGTTTAGGTAACTTGAACTGGATGTATTAGTTTGGTGACAA	
HEM3-gRNA-F1	TGAGGACGAAACGAGTAAGCTCGTCGATGTGATGTACCACTCCGTGTTTTAGAGCTAGAAATAGCAAGTTAAAATAAGGCTAGTCCGTT	Construction of pPIC3.5K-*HEM3*-gRNA
Cas9-backbone-R1	TGTTTGCCGGATCAAGAGCTACCAACTCT	
Cas9-backbone-F2	AGAGTTGGTAGCTCTTGATCCGGCAAACA	
HEM3-gRNA-R2	CGAGCTTACTCGTTTCGTCCTCACGGACTCATCAGGATGTGTTTGATTTGTTTAGGTAACTTGAACTGGATGTATTAGTTTGGTGACAA	
HEM4-gRNA-F1	TGAGGACGAAACGAGTAAGCTCGTCGCTGGCTTCGTGGATCTACGGTTTTAGAGCTAGAAATAGCAAGTTAAAATAAGGCTAGTCCGTT	Construction of pPIC3.5K-*HEM4*-gRNA
Cas9-backbone-R1	TGTTTGCCGGATCAAGAGCTACCAACTCT	
Cas9-backbone-F2	AGAGTTGGTAGCTCTTGATCCGGCAAACA	
HEM4-gRNA-R2	CGAGCTTACTCGTTTCGTCCTCACGGACTCATCAGGCTGGCTTTGATTTGTTTAGGTAACTTGAACTGGATGTATTAGTTTGGTGACAA	
pTEF-gRNA1-F1	TGAGGACGAAACGAGTAAGCTCGTCGCAAGATGGTTAAAAGGTGAGTTTTAGAGCTAGAAATAGCAAGTTAAAATAAGGCTAGTCCGTT	Construction of pPIC3.5K-P$_{TEF1}$UP-gRNA1
Cas9-backbone-R1	TGTTTGCCGGATCAAGAGCTACCAACTCT	
Cas9-backbone-F2	AGAGTTGGTAGCTCTTGATCCGGCAAACA	

pTEF-gRNA1-R2	CGAGCTTACTCGTTTCGTCCTCACGGACTCATCAGGCAAGATTTGATTTGTTTAGGTAACTTGAACTGGATGTATTAGTTTGGTGACAA	
HEM13-gRNA-F1	TGAGGACGAAACGAGTAAGCTCGTCGAATGAAACAACTAAGTGTGGTTTTAGAGCTAGAAATAGCAAGTTAAAATAAGGCTAGTCCGTT	Construction of pPIC3.5K-P_{HEM13}-gRNA
Cas9-backbone-R1	TGTTTGCCGGATCAAGAGCTACCAACTCT	
Cas9-backbone-F2	AGAGTTGGTAGCTCTTGATCCGGCAAACA	
HEM13-gRNA-R2	CGAGCTTACTCGTTTCGTCCTCACGGACTCATCAGGAATGATTTGATTTGTTTAGGTAACTTGAACTGGATGTATTAGTTTGGTGACAA	
HMX1-gRNA-F1	TGAGGACGAAACGAGTAAGCTCGTCAGCCACCAAGGGTTACTACGGTTTTAGAGCTAGAAATAGCAAGTTAAAATAAGGCTAGTCCGTT	Construction of pPIC3.5K-*HMX1*-gRNA
Cas9-backbone-R1	TGTTTGCCGGATCAAGAGCTACCAACTCT	
Cas9-backbone-F2	AGAGTTGGTAGCTCTTGATCCGGCAAACA	
HMX1-gRNA-R2	CGAGCTTACTCGTTTCGTCCTCACGGACTCATCAGAGCCACTTTGATTTGTTTAGGTAACTTGAACTGGATGTATTAGTTTGGTGACAA	
PMb-F	TGAAGCTCATCACCATCACCATCACATGGGTTTGTCTGATGGTGAATGGCA	Construction of pPICZαA-(P-Mb)$_{cn=1}$
PMb-R	CTGGCGGCCGCTTAACCTTGAAAACCTAATTCCTTGTACTTTGCAGCCA	
pPICZ-PMb-F	TTTTCAAGGTTAAGCGGCCGCCAGCTTTCTAGAA	
pPICZ-PMb-R	ACCCATGTGATGGTGATGGTGATGAGCTTCAGCCTCTCTTTTCTCGAGAGA	
SHb-F	TGAAGCTCATCACCATCACCATCACATGGGTGCTTTTACTGAAAAACAAGAAGCTTT	Construction of pPICZαA-(S-Hb)$_{cn=1}$
SHb-R	CTGGCGGCCGCTTAAAAAGCTTTCTTAATAGCTGCAGCTAATTCATCATAAGCA	
pPICZ-SHb-F	AGCTTTTTAAGCGGCCGCCAGCTTTCTAGAA	
pPICZ-SHb-R	CCCATGTGATGGTGATGGTGATGAGCTTCAGCCTCTCTTTTCTCGAGAGA	
VHb-F	AAACGCATCACCATCACCATCACATGCTCGACCAACAAACTATAAATATCATCAAAGCT	Construction of pPICZA-(V-Hb)$_{cn=1}$
VHb-R	CTGGCGGCCGCTTATTCCACAGCTTGTGCATAAAGATCGGCCT	
pPICZ-VHb-F	TGTGGAATAAGCGGCCGCCAGCTTTCTAGAA	
pPICZ-VHb-R	AGCATGTGATGGTGATGGTGATGCGTTTCGAATAATTAGTTGTTTTTTGATCTTCTCAAGTTGTCGT	
BM3-F	AAACGCATCACCATCACCATCACATGACCATCAAAGAAATGCCGCAGC	Construction of pPICZA-($BM3_{mut}$)$_{cn=1}$
BM3-R	CTGGCGGCCGCTTAGCCCGCCCAAACATCCTTCG	
pPICZ-BM3-F	GGGCTAAGCGGCCGCCAGCTTTCTAGAA	
pPICZ-BM3-R	GGTCATGTGATGGTGATGGTGATGCGTTTCGAATAATTAGTTGTTTTTTGATCTTCTCAAGTTGTCGT	
HEM1-F	CAATCAATTGAACAACTATTTCGAAATGGAGTTTGTCGCCCGTC	Construction of pGAPZA-*HEM1-linker-mSca*
HEM1-R	CAATCTGACTCCTGATGAGGTTTCGAT	
mScarlet-H1-F	CGAAACCTCATCAGGAGTCAGATTGGGTGCAGGAGCAGGTGCTGGTGCTGGAGCTGTTTCTAAAGGTGAAGCAGTTATTAAAGAATTCA	
mScarlet-R	AGATGAGTTTTTGTTCTAGAAAGCTCTATTTATATAATTCATCCATACCACCTGTACTATGTCTA	

pGAPZ-F	AGCTTTCTAGAACAAAAACTCATCTCAGAAGAG	
pGAPZ-R	TTCGAAATAGTTGTTCAATTGATTGAAATAGGGACA	
HEM2-F	CAATCAATTGAACAACTATTTCGAAATGGTGCATAAGGCTGAATACTTGGAC	Construction of pGAPZA-*HEM2-linker-mSca*
HEM2-R	TTCAGATAACCACTCCAGGAATTCAGGG	
mScarlet-H2-F	TGAATTCCTGGAGTGGTTATCTGAAGGTGCAGGAGCAGGTGCTGGTGCTGGAGCTGTTTCTAAAGGTGAAGCAGTTATTAAAGAATTCA	
mScarlet-R	AGATGAGTTTTTGTTCTAGAAAGCTCTATTTATATAATTCATCCATACCACCTGTACTATGTCTA	
pGAPZ-F	AGCTTTCTAGAACAAAAACTCATCTCAGAAGAG	
pGAPZ-R	TTCGAAATAGTTGTTCAATTGATTGAAATAGGGACA	
HEM3-F	CAATCAATTGAACAACTATTTCGAAATGAACCAAATCGAACAGAGCGG	Construction of pGAPZA-*HEM3-linker-mSca*
HEM3-R	ATGTAAACCCTCTTCCTTAATCTCTTTGATCTTG	
mScarlet-H3-F	AGAGATTAAGGAAGAGGGTTTACATGGTGCAGGAGCAGGTGCTGGTGCTGGAGCTGTTTCTAAAGGTGAAGCAGTTATTAAAGAATTCA	
mScarlet-R	AGATGAGTTTTTGTTCTAGAAAGCTCTATTTATATAATTCATCCATACCACCTGTACTATGTCTA	
pGAPZ-F	AGCTTTCTAGAACAAAAACTCATCTCAGAAGAG	
pGAPZ-R	TTCGAAATAGTTGTTCAATTGATTGAAATAGGGACA	
HEM4-F	CAATCAATTGAACAACTATTTCGAAATGCCAAAAGCCATTCTTCTGAAGAAT	Construction of pGAPZA-*HEM4-linker-mSca*
HEM4-R	GTGCACTTTTTGTATAGACAATAGTAAAGAGATAGGC	
mScarlet-H4-F	ACTATTGTCTATACAAAAAGTGCACGGTGCAGGAGCAGGTGCTGGTGCTGGAGCTGTTTCTAAAGGTGAAGCAGTTATTAAAGAATTCA	
mScarlet-R	AGATGAGTTTTTGTTCTAGAAAGCTCTATTTATATAATTCATCCATACCACCTGTACTATGTCTA	
pGAPZ-F	AGCTTTCTAGAACAAAAACTCATCTCAGAAGAG	
pGAPZ-R	TTCGAAATAGTTGTTCAATTGATTGAAATAGGGACA	
HEM12-F	CAATCAATTGAACAACTATTTCGAAATGAGTAGATTTCCAGAACTGAAGAATGACC	Construction of pGAPZA-*HEM12-linker-mSca*
HEM12-R	TTGAGATCCAATGCGATGACATTCTTGT	
mScarlet-H12-F	AGAATGTCATCGCATTGGATCTCAAGGTGCAGGAGCAGGTGCTGGTGCTGGAGCTGTTTCTAAAGGTGAAGCAGTTATTAAAGAATTCA	
mScarlet-R	AGATGAGTTTTTGTTCTAGAAAGCTCTATTTATATAATTCATCCATACCACCTGTACTATGTCTA	
pGAPZ-F	AGCTTTCTAGAACAAAAACTCATCTCAGAAGAG	
pGAPZ-R	TTCGAAATAGTTGTTCAATTGATTGAAATAGGGACA	
HEM13-F	CAATCAATTGAACAACTATTTCGAAATGGCCATCGACTCTGATATCAATCT	Construction of pGAPZA-*HEM13-linker-mSca*
HEM13-R	TACCCATTCAATAGGATTTTGTAGTACCTGC	
mScarlet-H13-F	ACTACAAAATCCTATTGAATGGGTAGGTGCAGGAGCAGGTGCTGGTGCTGGAGCTGTTTCTAAAGGTGAAGCAGTTATTAAAGAATTCA	

mScarlet-R	AGATGAGTTTTTGTTCTAGAAAGCTCTATTTATATAATTCATCCATACCACCTGTACTATGTCTA	
pGAPZ-F	AGCTTTCTAGAACAAAAACTCATCTCAGAAGAG	
pGAPZ-R	TTCGAAATAGTTGTTCAATTGATTGAAATAGGGACA	
HEM14-F	CAATCAATTGAACAACTATTTCGAAATGCTGAAAAGTCTTGCACCAAATTCC	Construction of pGAPZA-*HEM14-linker-mSca*
HEM14-R	AATGCCACTGAGGGTAGCCGAATCT	
mScarlet-H14-F	AGATTCGGCTACCCTCAGTGGCATTGGTGCAGGAGCAGGTGCTGGTGCTGGAGCTGTTTCTAAAGGTGAAGCAGTTATTAAAGAATTCA	
mScarlet-R	AGATGAGTTTTTGTTCTAGAAAGCTCTATTTATATAATTCATCCATACCACCTGTACTATGTCTA	
pGAPZ-F	AGCTTTCTAGAACAAAAACTCATCTCAGAAGAG	
pGAPZ-R	TTCGAAATAGTTGTTCAATTGATTGAAATAGGGACA	
HEM15-F	CAATCAATTGAACAACTATTTCGAAATGCTTAACCGTCGTTTCCAATCT	Construction of pGAPZA-*HEM15-linker-mSca*
HEM15-R	ATGGGCTCTGAAAAACTCTTTTGGATC	
mScarlet-H15-F	TCCAAAAGAGTTTTTCAGAGCCCATGGTGCAGGAGCAGGTGCTGGTGCTGGAGCTGTTTCTAAAGGTGAAGCAGTTATTAAAGAATTCA	
mScarlet-R	AGATGAGTTTTTGTTCTAGAAAGCTCTATTTATATAATTCATCCATACCACCTGTACTATGTCTA	
pGAPZ-F	AGCTTTCTAGAACAAAAACTCATCTCAGAAGAG	
pGAPZ-R	TTCGAAATAGTTGTTCAATTGATTGAAATAGGGACA	
Δ52HEM1-F	CAATCAATTGAACAACTATTTCGAAATGTCTCAACCTTCCAAGCCAG	Construction of pGAPZA-*HEM1*$_{53-561}$-*linker-mSca*
HEM1-R	CAATCTGACTCCTGATGAGGTTTCGAT	
mScarlet-H1-F	CGAAACCTCATCAGGAGTCAGATTGGGTGCAGGAGCAGGTGCTGGTGCTGGAGCTGTTTCTAAAGGTGAAGCAGTTATTAAAGAATTCA	
mScarlet-R	AGATGAGTTTTTGTTCTAGAAAGCTCTATTTATATAATTCATCCATACCACCTGTACTATGTCTA	
pGAPZ-F	AGCTTTCTAGAACAAAAACTCATCTCAGAAGAG	
pGAPZ-R	TTCGAAATAGTTGTTCAATTGATTGAAATAGGGACA	
Δ20HEM14-F	CAATCAATTGAACAACTATTTCGAAATGTTGACTTTCAGCTTTTTTTTGAATCGGTTGC	Construction of pGAPZA-*HEM14*$_{21-561}$-*linker-mSca*
HEM14-R	AATGCCACTGAGGGTAGCCGAATCT	
mScarlet-H14-F	AGATTCGGCTACCCTCAGTGGCATTGGTGCAGGAGCAGGTGCTGGTGCTGGAGCTGTTTCTAAAGGTGAAGCAGTTATTAAAGAATTCA	
mScarlet-R	AGATGAGTTTTTGTTCTAGAAAGCTCTATTTATATAATTCATCCATACCACCTGTACTATGTCTA	
pGAPZ-F	AGCTTTCTAGAACAAAAACTCATCTCAGAAGAG	
pGAPZ-R	TTCGAAATAGTTGTTCAATTGATTGAAATAGGGACA	
Δ40HEM14-F	CAATCAATTGAACAACTATTTCGAAATGAAATCCAAGCAGGTTGGAGGATGG	Construction of pGAPZA-*HEM14*$_{41-561}$-*linker-mSca*
HEM14-R	AATGCCACTGAGGGTAGCCGAATCT	
mScarlet-H14-F	AGATTCGGCTACCCTCAGTGGCATTGGTGCAGGAGCAGGTGCTGGTGCTGGAGCTGTTTCTAAAGGTGAAGCAGTTATTAAAGAATTCA	

mScarlet-R	AGATGAGTTTTTGTTCTAGAAAGCTCTATTTATATAATTCATCCATACCACCTGTACTATGTCTA	
pGAPZ-F	AGCTTTCTAGAACAAAAACTCATCTCAGAAGAG	
pGAPZ-R	TTCGAAATAGTTGTTCAATTGATTGAAATAGGGACA	
Δ60HEM14-F	CAATCAATTGAACAACTATTTCGAAATGAAGGGACCCAGAACTTTGAGAGG	Construction of pGAPZA-*HEM14*$_{61\text{-}561}$-*linker-mSca*
HEM14-R	AATGCCACTGAGGGTAGCCGAATCT	
mScarlet-H14-F	AGATTCGGCTACCCTCAGTGGCATTGGTGCAGGAGCAGGTGCTGGTGCTGGAGCTGTTTCTAAAGGTGAAGCAGTTATTAAAGAATTCA	
mScarlet-R	AGATGAGTTTTTGTTCTAGAAAGCTCTATTTATATAATTCATCCATACCACCTGTACTATGTCTA	
pGAPZ-F	AGCTTTCTAGAACAAAAACTCATCTCAGAAGAG	
pGAPZ-R	TTCGAAATAGTTGTTCAATTGATTGAAATAGGGACA	
Δ70HEM14-F	CAATCAATTGAACAACTATTTCGAAATGACGGGTACCTTGATGTTGTTGGAT	Construction of pGAPZA-*HEM14*$_{71\text{-}561}$-*linker-mSca*
HEM14-R	AATGCCACTGAGGGTAGCCGAATCT	
mScarlet-H14-F	AGATTCGGCTACCCTCAGTGGCATTGGTGCAGGAGCAGGTGCTGGTGCTGGAGCTGTTTCTAAAGGTGAAGCAGTTATTAAAGAATTCA	
mScarlet-R	AGATGAGTTTTTGTTCTAGAAAGCTCTATTTATATAATTCATCCATACCACCTGTACTATGTCTA	
pGAPZ-F	AGCTTTCTAGAACAAAAACTCATCTCAGAAGAG	
pGAPZ-R	TTCGAAATAGTTGTTCAATTGATTGAAATAGGGACA	
Δ80HEM14-F	CAATCAATTGAACAACTATTTCGAAATGACCAAGATAGGAGCAAATGACAAGGT	Construction of pGAPZA-*HEM14*$_{81\text{-}561}$-*linker-mSca*
HEM14-R	AATGCCACTGAGGGTAGCCGAATCT	
mScarlet-H14-F	AGATTCGGCTACCCTCAGTGGCATTGGTGCAGGAGCAGGTGCTGGTGCTGGAGCTGTTTCTAAAGGTGAAGCAGTTATTAAAGAATTCA	
mScarlet-R	AGATGAGTTTTTGTTCTAGAAAGCTCTATTTATATAATTCATCCATACCACCTGTACTATGTCTA	
pGAPZ-F	AGCTTTCTAGAACAAAAACTCATCTCAGAAGAG	
pGAPZ-R	TTCGAAATAGTTGTTCAATTGATTGAAATAGGGACA	
Δ20HEM15-F	CAATCAATTGAACAACTATTTCGAAATGGTGTTCATGAATATGGGTGGTCCCT	Construction of pGAPZA-*HEM15*$_{21\text{-}375}$-*linker-mSca*
HEM15-R	ATGGGCTCTGAAAAACTCTTTTGGATC	
mScarlet-H15-F	TCCAAAAGAGTTTTTCAGAGCCCATGGTGCAGGAGCAGGTGCTGGTGCTGGAGCTGTTTCTAAAGGTGAAGCAGTTATTAAAGAATTCA	
mScarlet-R	AGATGAGTTTTTGTTCTAGAAAGCTCTATTTATATAATTCATCCATACCACCTGTACTATGTCTA	
pGAPZ-F	AGCTTTCTAGAACAAAAACTCATCTCAGAAGAG	
pGAPZ-R	TTCGAAATAGTTGTTCAATTGATTGAAATAGGGACA	
Δ40HEM15-F	CAATCAATTGAACAACTATTTCGAAATGCTTTTCTCGGACGGAGATTTAATCCCG	Construction of pGAPZA-*HEM15*$_{41\text{-}375}$-*linker-mSca*
HEM15-R	ATGGGCTCTGAAAAACTCTTTTGGATC	
mScarlet-H15-F	TCCAAAAGAGTTTTTCAGAGCCCATGGTGCAGGAGCAGGTGCTGGTGCTGGAGCTGTTTCTAAAGGTGAAGCAGTTATTAAAGAATTCA	

mScarlet-R	AGATGAGTTTTTGTTCTAGAAAGCTCTATTTATATAATTCATCCATACCACCTGTACTATGTCTA	
pGAPZ-F	AGCTTTCTAGAACAAAAACTCATCTCAGAAGAG	
pGAPZ-R	TTCGAAATAGTTGTTCAATTGATTGAAATAGGGACA	
Δ60HEM15-F	CAATCAATTGAACAACTATTTCGAAATGATTGCAAGTAGAAGAACACCCAAAATTGAAT	Construction of pGAPZA-*HEM15*$_{61\text{-}375}$-*linker-mSca*
HEM15-R	ATGGGCTCTGAAAAACTCTTTTGGATC	
mScarlet-H15-F	TCCAAAAGAGTTTTTCAGAGCCCATGGTGCAGGAGCAGGTGCTGGTGCTGGAGCTGTTCTAAAGGTGAAGCAGTTATTAAAGAATTCA	
mScarlet-R	AGATGAGTTTTTGTTCTAGAAAGCTCTATTTATATAATTCATCCATACCACCTGTACTATGTCTA	
pGAPZ-F	AGCTTTCTAGAACAAAAACTCATCTCAGAAGAG	
pGAPZ-R	TTCGAAATAGTTGTTCAATTGATTGAAATAGGGACA	
Δ70HEM15-F	CAATCAATTGAACAACTATTTCGAAATGTCCTACTACAAAGCTATCGGAGGTGG	Construction of pGAPZA-*HEM15*$_{71\text{-}375}$-*linker-mSca*
HEM15-R	ATGGGCTCTGAAAAACTCTTTTGGATC	
mScarlet-H15-F	TCCAAAAGAGTTTTTCAGAGCCCATGGTGCAGGAGCAGGTGCTGGTGCTGGAGCTGTTCTAAAGGTGAAGCAGTTATTAAAGAATTCA	
mScarlet-R	AGATGAGTTTTTGTTCTAGAAAGCTCTATTTATATAATTCATCCATACCACCTGTACTATGTCTA	
pGAPZ-F	AGCTTTCTAGAACAAAAACTCATCTCAGAAGAG	
pGAPZ-R	TTCGAAATAGTTGTTCAATTGATTGAAATAGGGACA	
Δ80HEM15-F	CAATCAATTGAACAACTATTTCGAAATGCCTATCCGAAAGTGGTCTGAATACCAGA	Construction of pGAPZA-*HEM15*$_{81\text{-}375}$-*linker-mSca*
HEM15-R	ATGGGCTCTGAAAAACTCTTTTGGATC	
mScarlet-H15-F	TCCAAAAGAGTTTTTCAGAGCCCATGGTGCAGGAGCAGGTGCTGGTGCTGGAGCTGTTCTAAAGGTGAAGCAGTTATTAAAGAATTCA	
mScarlet-R	AGATGAGTTTTTGTTCTAGAAAGCTCTATTTATATAATTCATCCATACCACCTGTACTATGTCTA	
pGAPZ-F	AGCTTTCTAGAACAAAAACTCATCTCAGAAGAG	
pGAPZ-R	TTCGAAATAGTTGTTCAATTGATTGAAATAGGGACA	
pPICZ_HEM1-F	AGATCAAAAAACAACTAATTATTCGAAACGATGGAGTTTGTCGCCCGTCAGT	Construction of pPICZA-*HEM1*
pPICZ_HEM1-R	TGTTCTAGAAAGCTGGCGGCCGCCTACAATCTGACTCCTGATGAGGTTTCGATCTGCT	
pPICZ-F	GCGGCCGCCAGCTTTCTAGAACA	
pPICZ-R	CGTTTCGAATAATTAGTTGTTTTTTGATCTTCTCAAGTTGTCGT	
pPICZ_Δ52HEM1-F	AGATCAAAAAACAACTAATTATTCGAAACGATGTCTCAACCTTCCAAGCCAGCCC	Construction of pPICZA-*HEM1*$_{53\text{-}561}$
pPICZ_HEM1-R	TGTTCTAGAAAGCTGGCGGCCGCCTACAATCTGACTCCTGATGAGGTTTCGATCTGCT	
pPICZ-F	GCGGCCGCCAGCTTTCTAGAACA	
pPICZ-R	CGTTTCGAATAATTAGTTGTTTTTTGATCTTCTCAAGTTGTCGT	
pGAPZ_HEM1-F	CAATCAATTGAACAACTATTTCGAAATGGAGTTTGTCGCCCGTC	

pGAPZ_HEM1-R	AGATGAGTTTTTGTTCTAGAAAGCTCTACAATCTGACTCCTGATGAGGTTTCG	Construction of pGAPZA-*HEM1*
pGAPZ-F	AGCTTTCTAGAACAAAAACTCATCTCAGAAGAG	
pGAPZ-R	TTCGAAATAGTTGTTCAATTGATTGAAATAGGGACA	
pGAPZ_Δ52HEM1-F	CAATCAATTGAACAACTATTTCGAAATGTCTCAACCTTCCAAGCCAG	Construction of pGAPZA-*HEM1*$_{53-561}$
pGAPZ_HEM1-R	AGATGAGTTTTTGTTCTAGAAAGCTCTACAATCTGACTCCTGATGAGGTTTCG	
pGAPZ-F	AGCTTTCTAGAACAAAAACTCATCTCAGAAGAG	
pGAPZ-R	TTCGAAATAGTTGTTCAATTGATTGAAATAGGGACA	
pPICZ_GluTR$_E^{fbr}$-F	AGATCAAAAAACAACTAATTATTCGAAACGATGACCAAGAAGCTTTTAGCACTCGGTATCAACCATAAAACGGCACC	Construction of pPICZA-$GluTR_E^{fbr}$
pPICZ_GluTR$_E^{fbr}$-R	TGTTCTAGAAAGCTGGCGGCCGCCTACTCCAGCCCGAGGCTGTCG	
pPICZ-F	GCGGCCGCCAGCTTTCTAGAACA	
pPICZ-R	CGTTTCGAATAATTAGTTGTTTTTTGATCTTCTCAAGTTGTCGT	
pPICZ_GSAM$_E$-F	AGATCAAAAAACAACTAATTATTCGAAACGATGAGTAAGTCTGAAAATCTTTACAGCGCAGCGC	Construction of pPICZA-$GSAM_E$
pPICZ_GSAM$_E$-R	TGTTCTAGAAAGCTGGCGGCCGCTCACAACTTCGCAAACACCCGACGTG	
pPICZ-F	GCGGCCGCCAGCTTTCTAGAACA	
pPICZ-R	CGTTTCGAATAATTAGTTGTTTTTTGATCTTCTCAAGTTGTCGT	
pGAPZ_GluTR$_E^{fbr}$-F	CAATCAATTGAACAACTATTTCGAAATGACCAAGAAGCTTTTAGCACTCGGTATCAACCATAAAACG	Construction of pGAPZA-$GluTR_E^{fbr}$
pGAPZ_GluTR$_E^{fbr}$-R	AGATGAGTTTTTGTTCTAGAAAGCTCTACTCCAGCCCGAGGCTG	
pGAPZ-F	AGCTTTCTAGAACAAAAACTCATCTCAGAAGAG	
pGAPZ-R	TTCGAAATAGTTGTTCAATTGATTGAAATAGGGACA	
pGAPZ_GSAM$_E$-F	CAATCAATTGAACAACTATTTCGAAATGAGTAAGTCTGAAAATCTTTACAGCGC	Construction of pGAPZA-$GSAM_E$
pGAPZ_GSAM$_E$-R	AGATGAGTTTTTGTTCTAGAAAGCTTCACAACTTCGCAAACACCCGA	
pGAPZ-F	AGCTTTCTAGAACAAAAACTCATCTCAGAAGAG	
pGAPZ-R	TTCGAAATAGTTGTTCAATTGATTGAAATAGGGACA	
pPICZ_GluTR$_B^{fbr}$-F	AGATCAAAAAACAACTAATTATTCGAAACGATGCATAAGAAGATACTTGTTGTGGGAGTAGATTATAAATCCGCCCCT	Construction of pPICZA-$GluTR_B^{fbr}$
pPICZ_GluTR$_B^{fbr}$-R	TGTTCTAGAAAGCTGGCGGCCGCTCACTCACTTACAAGTGGGCTAAAGCCCG	
pPICZ-F	GCGGCCGCCAGCTTTCTAGAACA	
pPICZ-R	CGTTTCGAATAATTAGTTGTTTTTTGATCTTCTCAAGTTGTCGT	
pPICZ_GSAM$_B$-F	AGATCAAAAAACAACTAATTATTCGAAACGATGAGAAGCTATGAAAAATCAAAAACGGCTTTTAAAGAAGCGC	Construction of pPICZA-$GSAM_B$
pPICZ_GSAM$_B$-R	TGTTCTAGAAAGCTGGCGGCCGCTTATCTGCGGCTGATCTCAGCAAATACTTTCTCAGC	
pPICZ-F	GCGGCCGCCAGCTTTCTAGAACA	

pPICZ-R	CGTTTCGAATAATTAGTTGTTTTTTGATCTTCTCAAGTTGTCGT	
pGAPZ_GluTR$_B^{fbr}$-F	CAATCAATTGAACAACTATTTCGAAATGCATAAGAAGATACTTGTTGTGGGAGTAGATTATAAATCCGC	Construction of pGAPZA-*GluTR$_B^{fbr}$*
pGAPZ_GluTR$_B^{fbr}$-R	AGATGAGTTTTTGTTCTAGAAAGCTTCACTCACTTACAAGTGGGCTAAAGC	
pGAPZ-F	AGCTTTCTAGAACAAAAACTCATCTCAGAAGAG	
pGAPZ-R	TTCGAAATAGTTGTTCAATTGATTGAAATAGGGACA	
pGAPZ_GSAM$_B$-F	CAATCAATTGAACAACTATTTCGAAATGAGAAGCTATGAAAAATCAAAAACGGCT	Construction of pGAPZA-*GSAM$_B$*
pGAPZ_GSAM$_B$-R	AGATGAGTTTTTGTTCTAGAAAGCTTTATCTGCGGCTGATCTCAGCAAAT	
pGAPZ-F	AGCTTTCTAGAACAAAAACTCATCTCAGAAGAG	
pGAPZ-R	TTCGAAATAGTTGTTCAATTGATTGAAATAGGGACA	
RT_ARG4-F	TAGAAGTTCCTCCGGTGGCAGT	Quantitative Real-Time PCR (RT-PCR)
RT_ARG4-R	TCCATTGACTCCCGTTTTGAGGC	
RT_(P-Mb)-F	GAAGCAGAATTGACTCCATTAGCTCA	
RT_(P-Mb)-R	TCAGCACCAAAGTCACCAGGATG	
yz-pGAP-F	AGCCTCACATGCGACTATTATCGATCAATGAAATCC	Verification of the integration of expression cassettes based on pGAPZA and pPICZA
yz-pGAP-R	CGAAACCGTTAATACCGACAGTGATAGCCAT	
yz-pAOX-F	GCCTACAATGATGACATTTGGATTTGGTTGACTCAT	
yz-pAOX-R	AACTAGGATATCAAACTCTTCGGGGATAGCCAT	

[a] F, forward design of primers (top strand); R, reverse design of primers (bottom strand).
[b] The names of plasmids and gene knock-in/out cassettes can be found in Table S2 and Table S3.

Table S6. Strains used in this study

Strains	Description	Source
X33	Wild type	Invitrogen
KM71	*his4*, *aox1::ARG4*, *arg4*	Invitrogen
SMD1168	*his4*, *pep4*	Invitrogen
GS115	*his4*	Invitrogen
X33-P_{AOX1}-α-(P-Mb)$_{cn=1}$	Plasmid pPICZαA-(P-Mb)$_{cn=1}$ integrated into the 5′*AOX1* locus of X33; Zeo^R	This study
KM71-P_{AOX1}-α-(P-Mb)$_{cn=1}$	Plasmid pPICZαA-(P-Mb)$_{cn=1}$ integrated into the 5′*AOX1* locus of KM71; Zeo^R	This study
SMD1168-P_{AOX1}-α-(P-Mb)$_{cn=1}$	Plasmid pPICZαA-(P-Mb)$_{cn=1}$ integrated into the 5′*AOX1* locus of SMD1168; Zeo^R	This study
GS115-P_{AOX1}-α-(P-Mb)$_{cn=1}$	Plasmid pPICZαA-(P-Mb)$_{cn=1}$ integrated into the 5′*AOX1* locus of GS115; Zeo^R	This study
X33-P_{AOX1}-α-(P-Mb)$_{cn=2}$	Plasmid pPICZαA-(P-Mb)$_{cn=2}$ integrated into the 5′*AOX1* locus of X33; Zeo^R	This study
X33-P_{AOX1}-α-(P-Mb)$_{cn=3}$	Plasmid pPICZαA-(P-Mb)$_{cn=3}$ integrated into the 5′*AOX1* locus of X33; Zeo^R	This study
X33-*Δku70*	X33 with the *ku70* gene deleted	This study
X33-*Δku70-Δpep4*	X33-*Δku70* with the *pep4* gene deleted	This study
X33-*Δku70-Δyps1*	X33-*Δku70* with the *yps1-1* gene deleted	This study
X33-*Δku70-Δprb1*	X33-*Δku70* with the *prb1* gene deleted	This study
X33-*Δku70-Δpep4-Δyps1*	X33-*Δku70-Δpep4* with the *yps1-1* gene deleted	This study
X33-*Δku70-Δpep4-Δyps1-Δprb1*	X33-*Δku70-Δpep4-Δyps1* with the *prb1* gene deleted	This study
X33-*Δku70*-(P-Mb)	Plasmid pPICZαA-(P-Mb)$_{cn=1}$ integrated into the 5′*AOX1* locus of X33-*Δku70*; Zeo^R	This study
X33-*Δku70-Δpep4*-(P-Mb)	Plasmid pPICZαA-(P-Mb)$_{cn=1}$ integrated into the 5′*AOX1* locus of X33-*Δku70-Δpep4*; Zeo^R	This study
X33-*Δku70-Δyps1*-(P-Mb)	Plasmid pPICZαA-(P-Mb)$_{cn=1}$ integrated into the 5′*AOX1* locus of X33-*Δku70-Δyps1*; Zeo^R	This study
X33-*Δku70-Δprb1*-(P-Mb)	Plasmid pPICZαA-(P-Mb)$_{cn=1}$ integrated into the 5′*AOX1* locus of X33-*Δku70-Δprb1*; Zeo^R	This study
X33-*Δku70-Δpep4-Δyps1*-(P-Mb)	Plasmid pPICZαA-(P-Mb)$_{cn=1}$ integrated into the 5′*AOX1* locus of X33-*Δku70-Δpep4-Δyps1*; Zeo^R	This study
X33-*Δku70-Δpep4-Δyps1-Δprb1*-(P-Mb)	Plasmid pPICZαA-(P-Mb)$_{cn=1}$ integrated into the 5′*AOX1* locus of X33-*Δku70-Δpep4-Δyps1-Δprb1*; Zeo^R	This study
X33-*Δku70*-P_{GAP}-*PDI*	The *PDI* gene integrated into the P_{AOX1}UP-gRNA2 locus of X33-*Δku70*	This study
X33-*Δku70*-P_{GAP}-*KAR2*	The *KAR2* gene integrated into the P_{AOX1}UP-gRNA2 locus of X33-*Δku70*	This study
X33-*Δku70*-P_{GAP}-*Mxr1*	The *Mxr1* gene integrated into the P_{AOX1}UP-gRNA2 locus of X33-*Δku70*	This study
X33-*Δku70*-P_{GAP}-*Mit1*	The *Mit1* gene integrated into the P_{AOX1}UP-gRNA2 locus of X33-*Δku70*	This study
X33-*Δku70*-P_{GAP}-*Prm1*	The *Prm1* gene integrated into the P_{AOX1}UP-gRNA2 locus of X33-*Δku70*	This study
X33-*Δku70*-P_{GAP}-*PDI*-(P-Mb)	Plasmid pPICZαA-(P-Mb)$_{cn=1}$ integrated into the 5′*AOX1* locus of X33-*Δku70*-P_{GAP}-*PDI*; Zeo^R	This study

X33-*Δku70*-P$_{GAP}$-*KAR2*-(P-Mb)	Plasmid pPICZαA-(P-Mb)$_{cn=1}$ integrated into the 5′*AOX1* locus of X33-*Δku70*-P$_{GAP}$-*KAR2*; Zeo^{R}	This study
X33-*Δku70*-P$_{GAP}$-*Mxr1*-(P-Mb)	Plasmid pPICZαA-(P-Mb)$_{cn=1}$ integrated into the 5′*AOX1* locus of X33-*Δku70*-P$_{GAP}$-*Mxr1*; Zeo^{R}	This study
X33-*Δku70*-P$_{GAP}$-*Mit1*-(P-Mb)	Plasmid pPICZαA-(P-Mb)$_{cn=1}$ integrated into the 5′*AOX1* locus of X33-*Δku70*-P$_{GAP}$-*Mit1*; Zeo^{R}	This study
X33-*Δku70*-P$_{GAP}$-*Prm1*-(P-Mb)	Plasmid pPICZαA-(P-Mb)$_{cn=1}$ integrated into the 5′*AOX1* locus of X33-*Δku70*-P$_{GAP}$-*Prm1*; Zeo^{R}	This study
P1	The *Mit1* gene integrated into the P$_{AOX1}$UP-gRNA2 locus of X33-*Δku70*-*Δyps1*	This study
P1-(P-Mb)	Plasmid pPICZαA-(P-Mb)$_{cn=1}$ integrated into the 5′*AOX1* locus of P1; Zeo^{R}	This study
P1-(S-Hb)	Plasmid pPICZαA-(S-Hb)$_{cn=1}$ integrated into the 5′*AOX1* locus of P1; Zeo^{R}	This study
P1-(V-Hb)	Plasmid pPICZA-(V-Hb)$_{cn=1}$ integrated into the 5′*AOX1* locus of P1; Zeo^{R}	This study
P1-BM3$_{mut}$	Plasmid pPICZA-(BM3$_{mut}$)$_{cn=1}$ integrated into the 5′*AOX1* locus of P1; Zeo^{R}	This study
X33-*Δku70*-P$_{GAP}$-*HEM1-linker-mSca*	Plasmid pGAPZA-*HEM1-linker-mSca* integrated into the *5′GAP* locus of X33-*Δku70*; Zeo^{R}	This study
X33-*Δku70*-P$_{GAP}$-*HEM2-linker-mSca*	Plasmid pGAPZA-*HEM2-linker-mSca* integrated into the *5′GAP* locus of X33-*Δku70*; Zeo^{R}	This study
X33-*Δku70*-P$_{GAP}$-*HEM3-linker-mSca*	Plasmid pGAPZA-*HEM3-linker-mSca* integrated into the *5′GAP* locus of X33-*Δku70*; Zeo^{R}	This study
X33-*Δku70*-P$_{GAP}$-*HEM4-linker-mSca*	Plasmid pGAPZA-*HEM4-linker-mSca* integrated into the *5′GAP* locus of X33-*Δku70*; Zeo^{R}	This study
X33-*Δku70*-P$_{GAP}$-*HEM12-linker-mSca*	Plasmid pGAPZA-*HEM12-linker-mSca* integrated into the *5′GAP* locus of X33-*Δku70*; Zeo^{R}	This study
X33-*Δku70*-P$_{GAP}$-*HEM13-linker-mSca*	Plasmid pGAPZA-*HEM13-linker-mSca* integrated into the *5′GAP* locus of X33-*Δku70*; Zeo^{R}	This study
X33-*Δku70*-P$_{GAP}$-*HEM14-linker-mSca*	Plasmid pGAPZA-*HEM14-linker-mSca* integrated into the *5′GAP* locus of X33-*Δku70*; Zeo^{R}	This study
X33-*Δku70*-P$_{GAP}$-*HEM15-linker-mSca*	Plasmid pGAPZA-*HEM15-linker-mSca* integrated into the *5′GAP* locus of X33-*Δku70*; Zeo^{R}	This study
X33-*Δku70*-P$_{GAP}$-*HEM1*$_{53\text{-}561}$-*linker-mSca*	Plasmid pGAPZA-*HEM1*$_{53\text{-}561}$-*linker-mSca* integrated into the *5′GAP* locus of X33-*Δku70*; Zeo^{R}	This study
X33-*Δku70*-P$_{GAP}$-*HEM14*$_{21\text{-}561}$-*linker-mSca*	Plasmid pGAPZA-*HEM14*$_{21\text{-}561}$-*linker-mSca* integrated into the *5′GAP* locus of X33-*Δku70*; Zeo^{R}	This study
X33-*Δku70*-P$_{GAP}$-*HEM14*$_{41\text{-}561}$-*linker-mSca*	Plasmid pGAPZA-*HEM14*$_{41\text{-}561}$-*linker-mSca* integrated into the *5′GAP* locus of X33-*Δku70*; Zeo^{R}	This study

X33-*Δku70*-P_{GAP}-*HEM14*$_{61-561}$-*linker-mSca*	Plasmid pGAPZA-*HEM14*$_{61-561}$-*linker-mSca* integrated into the *5′GAP* locus of X33-*Δku70*; Zeo^R	This study
X33-*Δku70*-P_{GAP}-*HEM14*$_{71-561}$-*linker-mSca*	Plasmid pGAPZA-*HEM14*$_{71-561}$-*linker-mSca* integrated into the *5′GAP* locus of X33-*Δku70*; Zeo^R	This study
X33-*Δku70*-P_{GAP}-*HEM14*$_{81-561}$-*linker-mSca*	Plasmid pGAPZA-*HEM14*$_{81-561}$-*linker-mSca* integrated into the *5′GAP* locus of X33-*Δku70*; Zeo^R	This study
X33-*Δku70*-P_{GAP}-*HEM15*$_{21-375}$-*linker-mSca*	Plasmid pGAPZA-*HEM15*$_{21-375}$-*linker-mSca* integrated into the *5′GAP* locus of X33-*Δku70*; Zeo^R	This study
X33-*Δku70*-P_{GAP}-*HEM15*$_{41-375}$-*linker-mSca*	Plasmid pGAPZA-*HEM15*$_{41-375}$-*linker-mSca* integrated into the *5′GAP* locus of X33-*Δku70*; Zeo^R	This study
X33-*Δku70*-P_{GAP}-*HEM15*$_{61-375}$-*linker-mSca*	Plasmid pGAPZA-*HEM15*$_{61-375}$-*linker-mSca* integrated into the *5′GAP* locus of X33-*Δku70*; Zeo^R	This study
X33-*Δku70*-P_{GAP}-*HEM15*$_{71-375}$-*linker-mSca*	Plasmid pGAPZA-*HEM15*$_{71-375}$-*linker-mSca* integrated into the *5′GAP* locus of X33-*Δku70*; Zeo^R	This study
X33-*Δku70*-P_{GAP}-*HEM15*$_{81-375}$-*linker-mSca*	Plasmid pGAPZA-*HEM15*$_{81-375}$-*linker-mSca* integrated into the *5′GAP* locus of X33-*Δku70*; Zeo^R	This study
X33-*Δku70*-P_{GAP}-*HEM1*$_{53-561}$	Plasmid pGAPZA-*HEM1*$_{53-561}$ integrated into the 5′*GAP* locus of X33-*Δku70*; Zeo^R	This study
X33-*Δku70*-P_{AOX1}-*HEM1*$_{53-561}$	Plasmid pPICZA-*HEM1*$_{53-561}$ integrated into the 5′*AOX1* locus of X33-*Δku70*; Zeo^R	This study
X33-*Δku70*-P_{GAP}-*GluTR*$_E^{fbr}$-P_{GAP}-*GSAM*$_E$	Plasmid pGAPZA-*GluTR*$_E^{fbr}$-*GSAM*$_E$ integrated into the 5′*GAP* locus of X33-*Δku70*; Zeo^R	This study
X33-*Δku70*-P_{AOX1}-*GluTR*$_E^{fbr}$-P_{AOX1}-*GSAM*$_E$	Plasmid pPICZA-*GluTR*$_E^{fbr}$-*GSAM*$_E$ integrated into the 5′*AOX1* locus of X33-*Δku70*; Zeo^R	This study
X33-*Δku70*-P_{GAP}-*GluTR*$_B^{fbr}$-P_{GAP}-*GSAM*$_B$	Plasmid pGAPZA-*GluTR*$_B^{fbr}$-*GSAM*$_B$ integrated into the 5′*GAP* locus of X33-*Δku70*; Zeo^R	This study
X33-*Δku70*-P_{AOX1}-*GluTR*$_B^{fbr}$-P_{AOX1}-*GSAM*$_B$	Plasmid pPICZA-*GluTR*$_B^{fbr}$-*GSAM*$_B$ integrated into the 5′*AOX1* locus of X33-*Δku70*; Zeo^R	This study
X33-*Δku70*-P_{GAP}-*HEM1*	Plasmid pGAPZA-*HEM1* integrated into the 5′*GAP* locus of X33-*Δku70*; Zeo^R	This study
X33-*Δku70*-P_{AOX1}-*HEM1*	Plasmid pPICZA-*HEM1* integrated into the 5′*AOX1* locus of X33-*Δku70*; Zeo^R	This study
HEME-1	The *HEM1* gene integrated into the *AOXTT*DOWN-gRNA locus of X33-*Δku70*	This study
HEME-2	The native *HEM14* and *HEM15* in the X33-*Δku70* genome were replaced by their MLS-truncated version (*HEM14*$_{71-561}$ and *HEM15*$_{71-375}$)	This study
HEME-3	The native *HEM2*, *HEM3*, and *HEM4* in the X33-*Δku70* genome were replaced by *HEM2-linker-GBD ligand*, *HEM3-linker-SH3 ligand*, and *HEM4-linker-PDZ ligand*, respectively	This study

HEME-4	The protein scaffold P_{GAP}-*GBD domain-linker-SH3 domain-linker-PDZ domain* integrated into P_{TEF1}UP-gRNA1 locus of HEME-3	This study
HEME-5	The protein scaffold P_{G7}-*GBD domain-linker-SH3 domain-linker-PDZ domain* integrated into P_{TEF1}UP-gRNA1 locus of HEME-3	This study
HEME-6	The strategies used in HEME-1 and HEME-2 strains were applied in the HEME-5 strain	This study
HEME-7	The native promoter of *HEM13* in the HEME-6 genome was replaced by P_{GAP}	This study
HEME-8	HEME-6 with the *HMX1* gene deleted	This study
HEME-9	HEME-7 with the *HMX1* gene deleted	This study
P1H9	HEME-9 with the *Mit1* gene integrated into P_{AOX1}UP-gRNA2 locus and the *yps1-1* gene deleted	This study
P1H9-(P-Mb)	Plasmid pPICZαA-(P-Mb)$_{cn=1}$ integrated into 5′*AOX1* locus of P1H9	This study
P1H9-(S-Hb)	Plasmid pPICZαA-(S-Hb)$_{cn=1}$ integrated into 5′*AOX1* locus of P1H9	This study
P1H9-(V-Hb)	Plasmid pPICZA-(V-Hb)$_{cn=1}$ integrated into 5′*AOX1* locus of P1H9	This study
P1H9-BM3$_{mut}$	Plasmid pPICZA-(BM3$_{mut}$)$_{cn=1}$ integrated into 5′*AOX1* locus of P1H9	This study

Table S7. Theoretical amplification length of colony PCR for the single integration of expression cassettes based on pPICZA and pGAPZA

NO.	Strains	Theoretical length (kb)
1	X33-P$_{AOX1}$-α-(P-Mb)$_{cn=1}$	5.1
2	KM71-P$_{AOX1}$-α-(P-Mb)$_{cn=1}$	5.1
3	SMD1168-P$_{AOX1}$-α-(P-Mb)$_{cn=1}$	5.1
4	GS115-P$_{AOX1}$-α-(P-Mb)$_{cn=1}$	5.1
5	X33-P$_{AOX1}$-α-(P-Mb)$_{cn=2}$	7.2
6	X33-P$_{AOX1}$-α-(P-Mb)$_{cn=3}$	9.3
7	X33-*Δku70*-(P-Mb)	5.1
8	X33-*Δku70-Δpep4*-(P-Mb)	5.1
9	X33-*Δku70-Δyps1*-(P-Mb)	5.1
10	X33-*Δku70-Δprb1*-(P-Mb)	5.1
11	X33-*Δku70-Δpep4-Δyps1*-(P-Mb)	5.1
12	X33-*Δku70-Δpep4-Δyps1-Δprb1*-(P-Mb)	5.1
13	X33-*Δku70*-P$_{GAP}$-*PDI*-(P-Mb)	5.1
14	X33-*Δku70*-P$_{GAP}$-*KAR2*-(P-Mb)	5.1
15	X33-*Δku70*-P$_{GAP}$-*Mxr1*-(P-Mb)	5.1
16	X33-*Δku70*-P$_{GAP}$-*Mit1*-(P-Mb)	5.1
17	X33-*Δku70*-P$_{GAP}$-*Prm1*-(P-Mb)	5.1
18	P1-(P-Mb)	5.1
19	P1-(S-Hb)	5.1
20	P1-(V-Hb)	4.8
21	P1-BM3$_{mut}$	7.5
22	X33-*Δku70*-P$_{GAP}$-*HEM1-linker-mSca*	5.8
23	X33-*Δku70*-P$_{GAP}$-*HEM2-linker-mSca*	5.2
24	X33-*Δku70*-P$_{GAP}$-*HEM3-linker-mSca*	5.2
25	X33-*Δku70*-P$_{GAP}$-*HEM4-linker-mSca*	4.9
26	X33-*Δku70*-P$_{GAP}$-*HEM12-linker-mSca*	5.2
27	X33-*Δku70*-P$_{GAP}$-*HEM13-linker-mSca*	5.1
28	X33-*Δku70*-P$_{GAP}$-*HEM14-linker-mSca*	5.8
29	X33-*Δku70*-P$_{GAP}$-*HEM15-linker-mSca*	5.3
30	X33-*Δku70*-P$_{GAP}$-*HEM1*$_{53\text{-}561}$-*linker-mSca*	5.7
31	X33-*Δku70*-P$_{GAP}$-*HEM14*$_{21\text{-}561}$-*linker-mSca*	5.8
32	X33-*Δku70*-P$_{GAP}$-*HEM14*$_{41\text{-}561}$-*linker-mSca*	5.7
33	X33-*Δku70*-P$_{GAP}$-*HEM14*$_{61\text{-}561}$-*linker-mSca*	5.6
34	X33-*Δku70*-P$_{GAP}$-*HEM14*$_{71\text{-}561}$-*linker-mSca*	5.6
35	X33-*Δku70*-P$_{GAP}$-*HEM14*$_{81\text{-}561}$-*linker-mSca*	5.6
36	X33-*Δku70*-P$_{GAP}$-*HEM15*$_{21\text{-}375}$-*linker-mSca*	5.2
37	X33-*Δku70*-P$_{GAP}$-*HEM15*$_{41\text{-}375}$-*linker-mSca*	5.2
38	X33-*Δku70*-P$_{GAP}$-*HEM15*$_{61\text{-}375}$-*linker-mSca*	5.1
39	X33-*Δku70*-P$_{GAP}$-*HEM15*$_{71\text{-}375}$-*linker-mSca*	5.1
40	X33-*Δku70*-P$_{GAP}$-*HEM15*$_{81\text{-}375}$-*linker-mSca*	5.0
41	X33-*Δku70*-P$_{GAP}$-*HEM1*$_{53\text{-}561}$	4.9
42	X33-*Δku70*-P$_{AOX1}$-*HEM1*$_{53\text{-}561}$	5.9
43	X33-*Δku70*-P$_{GAP}$-*GluTR*$_{E}^{fbr}$-P$_{GAP}$-*GSAM*$_{E}$	6.9
44	X33-*Δku70*-P$_{AOX1}$-*GluTR*$_{E}^{fbr}$-P$_{AOX1}$-*GSAM*$_{E}$	8.2
45	X33-*Δku70*-P$_{GAP}$-*GluTR*$_{B}^{fbr}$-P$_{GAP}$-*GSAM*$_{B}$	7.0
46	X33-*Δku70*-P$_{AOX1}$-*GluTR*$_{B}^{fbr}$-P$_{AOX1}$-*GSAM*$_{B}$	8.4
47	X33-*Δku70*-P$_{GAP}$-*HEM1*	5.1
48	X33-*Δku70*-P$_{AOX1}$-*HEM1*	6.0
49	P1H9-(P-Mb)	5.1
50	P1H9-(S-Hb)	5.1

51	P1H9-(V-Hb)	4.8
52	P1H9-BM3$_{mut}$	7.5

Supplementary Notes

Heterologous genes, P_{G7} promoter, and original CRISPR/Cas9 plasmid sequences used in this study (5'-3').

1. Gene sequence of P-Mb
ATGGGTTTGTCTGATGGTGAATGGCAATTGGTTTTAAATGTTTGGGGTAAAGTTG
AAGCTGATGTTGCAGGTCATGGTCAAGAAGTTTTGATCAGATTGTTTAAAGGTCA
TCCAGAAACTTTGGAAAAGTTCGATAAGTTTAAACATTTGAAGTCTGAAGATGAA
ATGAAGGCTTCAGAAGATTTGAAGAAACATGGTAACACTGTTTTGACAGCTTTGG
GTGGTATTTTGAAAAAGAAAGGTCATCATGAAGCAGAATTGACTCCATTAGCTCA
ATCTCATGCAACAAAGCATAAGATCCCTGTTAAGTATTTGGAATTCATTTCTGAA
GCAATCATCCAAGTTTTACAATCAAAACATCCTGGTGACTTTGGTGCTGATGCAC
AAGGTGCTATGTCAAAGGCATTGGAATTGTTTAGAAACGATATGGCTGCAAAGTA
CAAGGAATTAGGTTTTCAAGGTTAA

2. Gene sequence of S-Hb
ATGGGTGCTTTTACTGAAAAACAAGAAGCTTTGGTTTCTTCATCTTTCGAAGCTTT
TAAAGCTAACATCCCACAATACTCAGTTGTTTTCTATACATCTATCTTAGAAAAAG
CTCCAGCTGCAAAGGATTTGTTTTCATTTTTATCTAACGGTGTTGATCCATCAAAT
CCAAAATTGACTGGTCATGCAGAAAAGTTGTTCGGTTTAGTTAGAGATTCTGCAG
GTCAATTGAAAGCTAATGGTACAGTTGTTGCTGATGCTGCATTAGGTTCAATTCAT
GCACAAAAAGCTATCACTGATCCACAATTCGTTGTTGTTAAGGAAGCATTGTTGA
AGACAATTAAAGAAGCTGTTGGTGACAAATGGTCTGATGAATTGTCATCTGCATG
GGAAGTTGCTTATGATGAATTAGCTGCAGCTATTAAGAAAGCTTTTTAA

3. Gene sequence of V-Hb
ATGCTCGACCAACAAACTATAAATATCATCAAAGCTACTGTGCCCGTCCTGAAAG
AGCACGGCGTTACAATTACCACGACGTTCTACAAGAACCTGTTTGCGAAGCACCC
GGAGGTACGGCCACTTTTCGACATGGGAAGACAAGAATCTTTAGAACAGCCCAA
AGCACTAGCTATGACAGTTTTGGCGGCGGCGCAGAATATAGAAAACTTACCAGC
AATATTACCGGCGGTTAAAAAGATTGCTGTCAAGCACTGTCAGGCAGGGGTCGCC
GCTGCACATTATCCTATCGTGGGCCAAGAATTGTTAGGGGCCATTAAAGAGGTAC
TAGGTGACGCGGCAACCGATGATATCCTCGACGCTTGGGGAAAGGCCTACGGTGT
TATTGCCGATGTATTTATACAGGTAGAGGCCGATCTTTATGCACAAGCTGTGGAA
TAA

4. Gene sequence of $BM3_{mut}$ (A82F/A328F)
ATGACCATCAAAGAAATGCCGCAGCCGAAGACCTTTGGCGAGCTGAAAAACCTG
CCGCTGCTGAACACCGACAAGCCGGTGCAAGCGCTGATGAAAATCGCGGATGAA
CTGGGCGAGATTTTCAAGTTTGAGGCGCCGGGCCGTGTTACCCGTTACCTGAGCA
GCCAGCGTCTGATCAAAGAGGCGTGCGACGAAAGCCGTTTCGATAAGAACCTGA
GCCAAGCGCTGAAATTTGTGCGTGACTTCTTTGGTGATGGCCTGTTCACCAGCTG
GACCCACGAAAAGAACTGGAAGAAAGCGCACAACATCCTGCTGCCGAGCTTCAG
CCAGCAAGCGATGAAGGGTTATCACGCGATGATGGTGGACATTGCGGTGCAGCT
GGTTCAAAAATGGGAGCGTCTGAACGCGGATGAACACATCGAGGTTCCGGAAGA
CATGACCCGTCTGACCCTGGATACCATTGGCCTGTGCGGTTTTAACTACCGTTTCA
ACAGCTTTTATCGTGACCAGCCGCACCCGTTCATCACCAGCATGGTTCGTGCGCT
GGATGAAGCGATGAACAAGCTGCAACGTGCGAACCCGGACGATCCGGCGTACGA
CGAAAACAAGCGTCAGTTTCAAGAGGATATTAAAGTGATGAACGACCTGGTTGA

TAAGATCATTGCGGACCGTAAAGCGAGCGGCGAGCAGAGCGACGATCTGCTGAC
CCACATGCTGAACGGTAAAGATCCGGAGACCGGCGAACCGCTGGACGATGAAAA
CATCCGTTACCAAATCATTACCTTCCTGATTGCGGGTCATGAGACCACCAGCGGT
CTGCTGAGCTTCGCGCTGTATTTTCTGGTGAAGAACCCGCACGTGCTGCAAAAGG
CGGCGGAGGAAGCGGCGCGTGTGCTGGTTGACCCGGTGCCGAGCTACAAGCAGG
TTAAACAACTGAAGTATGTGGGTATGGTTCTGAACGAAGCGCTGCGTCTGTGGCC
GACCTTCCCGGCGTTTAGCCTGTACGCGAAAGAGGACACCGTGCTGGGTGGCGAG
TATCCGCTGGAAAAAGGTGACGAGCTGATGGTTCTGATCCCGCAGCTGCACCGTG
ATAAGACCATTTGGGGCGACGATGTGGAGGAGTTCCGTCCGGAGCGTTTTGAAAA
CCCGAGCGCGATCCCGCAGCACGCGTTCAAACCGTTTGGTAACGGCCAACGTGCG
TGCATTGGTCAGCAATTTGCGCTGCACGAAGCGACCCTGGTTCTGGGCATGATGC
TGAAGCACTTCGACTTTGAGGATCACACCAACTACGAACTGGACATCAAGGAGA
CCCTGACCCTGAAACCGGAGGGTTTCGTGGTTAAGGCGAAAAGCAAGAAAATCC
CGCTGGGTGGCATTCCGAGCCCGAGCACCGAACAGAGCGCGAAGAAAGTGCGTA
AGAAAGCGGAGAACGCGCACAACACCCCGCTGCTGGTTCTGTACGGCAGCAACA
TGGGCACCGCGGAGGGCACCGCGCGTGACCTGGCGGACATCGCGATGAGCAAAG
GCTTTGCGCCGCAAGTGGCGACCCTGGACAGCCATGCGGGTAACCTGCCGCGTGA
GGGTGCGGTGCTGATTGTTACCGCGAGCTATAACGGTCACCCGCCGGATAACGCG
AAGCAGTTCGTTGACTGGCTGGATCAAGCGAGCGCGGACGAAGTGAAAGGCGTT
CGTTACAGCGTGTTTGGTTGCGGCGATAAGAACTGGGCGACCACCTATCAGAAAG
TTCCGGCGTTCATTGATGAGACCCTGGCGGCGAAGGGTGCGGAAAACATTGCGG
ACCGTGGCGAGGCGGATGCGAGCGACGATTTTGAAGGCACCTACGAGGAATGGC
GTGAGCACATGTGGAGCGATGTGGCGGCGTATTTCAACCTGGACATCGAGAACA
GCGAAGATAACAAGAGCACCCTGAGCCTGCAATTTGTTGACAGCGCGGCGGATA
TGCCGCTGGCGAAGATGCACGGTGCGTTCAGCACCAACGTGGTTGCGAGCAAAG
AGCTGCAACAACCGGGCAGCGCGCGTAGCACCCGTCACCTGGAAATCGAGCTGC
CGAAAGAAGCGAGCTACCAAGAGGGTGACCACCTGGGCGTGATCCCGCGTAACT
ATGAAGGTATTGTGAACCGTGTTACCGCGCGTTTTGGCCTGGATGCGAGCCAGCA
AATTCGTCTGGAGGCGGAGGAAGAGAAGCTGGCGCACCTGCCGCTGGCGAAAAC
CGTGAGCGTTGAAGAGCTGCTGCAATACGTGGAGCTGCAAGACCCGGTTACCCGT
ACCCAGCTGCGTGCGATGGCGGCGAAGACCGTGTGCCCGCCGCACAAAGTTGAA
CTGGAGGCGCTGCTGGAAAAACAGGCGTACAAGGAGCAAGTTCTGGCGAAGCGT
CTGACCATGCTGGAGCTGCTGGAAAAGTATCCGGCGTGCGAAATGAAATTCAGC
GAGTTTATCGCGCTGCTGCCGAGCATTCGTCCGCGTTACTATAGCATCAGCAGCA
GCCCGCGTGTGGACGAAAAGCAGGCGAGCATTACCGTTAGCGTGGTTAGCGGTG
AAGCGTGGAGCGGTTACGGCGAGTATAAAGGCATCGCGAGCAACTATCTGGCGG
AGCTGCAAGAGGGTGACACCATCACCTGCTTCATTAGCACCCCGCAAAGCGAATT
TACCCTGCCGAAAGATCCGGAGACCCCGCTGATTATGGTTGGTCCGGGCACCGGT
GTTGCGCCGTTCCGTGGCTTTGTGCAGGCGCGTAAACAACTGAAGGAACAGGGTC
AAAGCCTGGGCGAGGCGCACCTGTATTTCGGTTGCCGTAGCCCGCACGAGGACTA
CCTGTATCAGGAAGAGCTGGAAAACGCGCAAAGCGAGGGCATCATTACCCTGCA
CACCGCGTTCAGCCGTATGCCGAACCAGCCGAAGACCTATGTGCAGCACGTTATG
GAACAAGACGGTAAGAAACTGATCGAGCTGCTGGATCAGGGCGCGCACTTCTAC
ATTTGCGGTGATGGTAGCCAAATGGCTCCGGCGGTGGAAGCGACCCTGATGAAA
AGCTATGCGGACGTGCACCAAGTTAGCGAGGCGGATGCGCGTCTGTGGCTGCAA
CAACTGGAAGAGAAAGGTCGTTACGCGAAGGATGTTTGGGCGGGCTAA

5. Gene sequence of m-Scarlet
ATGGTTTCTAAAGGTGAAGCAGTTATTAAAGAATTCATGAGATTCAAAGTTCATA
TGGAAGGTAGTATGAATGGTCATGAATTTGAAATTGAAGGTGAAGGTGAAGGTA
GACCATATGAAGGTACTCAAACTGCTAAATTGAAAGTTACTAAAGGTGGTCCATT

ACCATTTTCATGGGATATTTTGTCACCACAATTCATGTATGGTTCAAGAGCTTTTA
TTAAACATCCAGCTGATATTCCAGATTATTATAAACAATCTTTTCCAGAAGGTTTT
AAATGGGAAAGAGTTATGAATTTCGAAGATGGTGGTGCTGTTACTGTTACTCAAG
ATACTTCATTAGAAGATGGTACTTTGATCTATAAAGTTAAATTGAGAGGTACTAA
TTTTCCACCAGATGGTCCAGTTATGCAAAAGAAAACTATGGGTTGGGAAGCTTCT
ACTGAAAGATTATATCCAGAAGATGGTGTTTTGAAAGGAGATATTAAAATGGCTT
TGAGATTGAAAGATGGTGGTAGATATTTGGCTGATTTCAAAACTACTTATAAAGC
TAAGAAACCAGTTCAAATGCCAGGTGCTTATAATGTTGATAGAAAATTGGATATT
ACTTCACATAATGAAGATTATACTGTTGTTGAACAATATGAAAGAAGTGAAGGTA
GACATAGTACAGGTGGTATGGATGAATTATATAAATAG

6. Gene sequence of GluTR$_E^{fbr}$

ATGACCAAGAAGCTTTTAGCACTCGGTATCAACCATAAAACGGCACCTGTATCGC
TGCGAGAACGTGTATCGTTTTCGCCGGATAAGCTCGATCAGGCGCTTGACAGCCT
GCTTGCGCAGCCGATGGTGCAGGGCGGCGTGGTGCTGTCGACGTGCAACCGCAC
GGAACTTTATCTTAGCGTTGAAGAGCAGGATAACCTGCAAGAGGCGTTAATCCGC
TGGCTTTGCGATTATCACAATCTTAATGAAGAAGATCTGCGTAAAAGCCTCTACT
GGCATCAGGATAACGACGCGGTTAGCCATTTAATGCGTGTTGCCAGCGGCCTGGA
TTCATTGGTTCTTGGGGAGCCGCAGATCCTCGGTCAGGTTAAAAAAGCGTTTGCC
GATTCGCAAAAAGGCCATATGAAGGCCAGCGAACTGGAACGCATGTTCCAGAAA
TCTTTCTCTGTAGCGAAACGCGTTCGCACTGAAACAGATATCGGTGCCAGCGCTG
TGTCTGTCGCTTTTGCGGCTTGTACGCTGGCGCGGCAGATCTTTGAATCGCTCTCT
ACGGTCACAGTGTTGCTGGTAGGCGCGGGCGAAACCATCGAGCTGGTAGCGCGT
CATCTGCGCGAACATAAAGTACAGAAGATGATTATCGCCAACCGCACTCGCGAA
CGTGCCCAAATACTGGCAGATGAAGTTGGCGCGGAAGTGATTGCCCTGAGTGAG
ATCGACGAACGTCTGCGCGAAGCCGATATCATCATCAGTTCCACCGCCAGCCCGT
TACCGATTATCGGGAAAGGCATGGTGGAGCGCGCATTAAAAAGCCGTCGCAACC
AACCAATGCTGTTGGTGGATATTGCCGTTCCGCGCGATGTTGAGCCGGAAGTTGG
CAAACTGGCGAATGCTTATCTTTATAGCGTGGACGATCTGCAAAGCATCATTTCG
CACAACCTGGCGCAGCGTAAAGCCGCAGCGGTTGAGGCGGAAACTATTGTCGCT
CAGGAAACCAGCGAATTTATGGCGTGGCTGCGAGCACAAAGCGCCAGCGAAACC
ATTCGCGAGTATCGCAGCCAGGCAGAGCAAGTTCGCGATGAGTTAACCGCCAAA
GCGTTAGCGGCCCTTGAGCAGGGCGGCGACGCGCAAGCCATTATGCAGGATCTG
GCATGGAAACTGACTAACCGCTTGATCCATGCGCCAACGAAATCACTTCAACAGG
CCGCCCGTGACGGGGATAACGAACGCCTGAATATTCTGCGCGACAGCCTCGGGCT
GGAGTAG

7. Gene sequence of GSAM$_E$

ATGAGTAAGTCTGAAAATCTTTACAGCGCAGCGCGCGAGCTGATCCCTGGCGGTG
TGAACTCCCCTGTTCGCGCCTTTACTGGCGTGGGCGGCACTCCACTGTTTATCGAA
AAAGCGGACGGCGCTTATCTGTACGATGTTGATGGCAAAGCCTATATCGATTATG
TCGGTTCCTGGGGGCCGATGGTGCTGGGCCATAACCATCCGGCAATCCGCAATGC
CGTGATTGAAGCCGCCGAGCGTGGTTTAAGCTTTGGTGCACCAACCGAAATGGAA
GTGAAAATGGCGCAACTGGTGACTGAACTGGTCCCGACCATGGATATGGTGCGC
ATGGTGAACTCCGGCACCGAGGCGACGATGAGCGCCATCCGCCTGGCCCGTGGTT
TTACCGGTCGCGACAAAATTATTAAATTTGAAGGTTGTTACCACGGTCACGCTGA
CTGCCTGCTGGTGAAAGCCGGTTCTGGCGCACTCACGTTAGGCCAGCCAAACTCG
CCGGGCGTTCCGGCAGATTTCGCCAAACATACCTTAACCTGTACTTATAACGATC
TGGCTTCTGTACGCGCCGCGTTTGAGCAATACCCGCAAGAGATTGCCTGTATTAT
CGTCGAGCCGGTGGCAGGCAATATGAACTGCGTTCCACCGCTGCCAGAGTTCCTG
CCAGGTCTGCGTGCGCTGTGCGACGAATTTGGCGCATTGCTGATCATCGATGAAG

TAATGACCGGCTTCCGCGTGGCACTGGCTGGCGCACAGGATTATTACGGTGTGGA
ACCGGATCTCACCTGCCTGGGCAAAATCATCGGCGGTGGAATGCCGGTAGGCGC
ATTCGGTGGTCGTCGTGATGTAATGGATGCGCTGGCCCCGACGGGTCCGGTCTAT
CAGGCGGGTACGCTTTCCGGTAACCCAATTGCGATGGCAGCGGGTTTCGCCTGTC
TGAATGAAGTCGCGCAGCCGGGCGTTCACGAAACGTTGGATGAGCTGACATCAC
GTCTGGCAGAAGGTCTGCTGGAAGCGGCAGAAGAAGCCGGAATTCCGCTGGTCG
TTAACCACGTTGGCGGCATGTTCGGTATTTCTTTACCGACGCCGAGTCCGTGACG
TGCTATCAGGATGTGATGGCCTGTGACGTGGAACGCTTTAAGCGTTTCTTCCATAT
GATGCTGGACGAAGGTGTTTACCTGGCACCGTCAGCGTTTGAAGCGGGCTTTATG
TCCGTGGCGCACAGCATGGAAGATATCAATAACACCATCGATGCTGCACGTCGGG
TGTTTGCGAAGTTGTGA

8. Gene sequence of GluTR$_B^{fbr}$

ATGCATAAGAAGATACTTGTTGTGGGAGTAGATTATAAATCCGCCCCTATTGAGA
TACGTGAAAAAGTAAGTTTTCAGCCGAATGAGCTGGCAGAAGCAATGGTGCAGC
TGAAAGAAGAGAAAAGCATTCTTGAAAACATCATTGTCTCAACCTGCAACCGCAC
TGAAATTTATGCGGTAGTCGACCAGCTTCATACCGGCCGTTATTATATAAAAAAG
TTTTTAGCTGATTGGTTTCAATTAAGCAAAGAAGAGCTGTCACCGTTCTTAACGTT
TTATGAGAGCGATGCCGCTGTTGAGCATTTATTCCGTGTAGCCTGCGGACTTGATT
CTATGGTGATTGGCGAAACGCAGATTCTCGGACAGGTACGCGACAGCTTTAAAAC
AGCTCAGCAAGAAAAAACGATCGGGACTATTTTTAATGAGCTGTTTAAGCAGGCA
GTTACAGTGGGCAAACGGACTCACGCCGAAACAGACATTGGCTCAAATGCGGTG
TCAGTAAGCTATGCTGCAGTTGAACTTGCCAAAAAAATCTTCGGAAATCTTTCAA
GCAAGCACATATTGATTCTCGGTGCGGGAAAAATGGGCGAGCTTGCTGCGGAAA
ACCTGCACGGACAGGGAATCGGCAAGGTCACTGTCATTAACCGAACATACTTGA
AAGCGAAGGAGCTTGCAGACCGTTTTTCAGGTGAAGCGAGAAGCTTGAATCAGC
TTGAAAGCGCGCTTGCGGAGGCTGATATTTTAATCAGTTCAACCGGTGCAAGTGA
ATTTGTCGTGTCCAAAGAGATGATGGAAAACGCGAATAAGCTTCGCAAGGGACG
TCCGCTGTTTATGGTCGACATTGCCGTGCCTAGAGATCTTGATCCGGCGCTGAATG
ATCTTGAAGGTGTTTTTCTTTATGATATCGACGATCTGGAAGGCATTGTAGAAGC
GAACATGAAAGAGCGGAGAGAAACAGCTGAAAAAGTTGAACTGTTAATTGAAGA
AACCATTGTGGAATTTAAACAATGGATGAATACACTTGGTGTTGTGCCTGTTATTT
CTGCATTGCGCGAAAAGGCGCTTGCCATCCAGTCAGAAACGATGGACAGCATTG
AGCGTAAGCTGCCTCACTTAAGCACAAGAGAGAAAAAACTGTTGAACAAACACA
CCAAAAGTATTATTAACCAAATGCTTCGTGATCCGATTTTAAAGGTGAAAGAGCT
TGCGGCAGATGCTGATTCTGAAGAAAAGCTCGCGTTGTTTATGCAGATTTTTGAT
ATTGAAGAAGCTGCGGGCCGTCAAATGATGAAAACCGTTGAAAGCAGCCAGAAG
GTCCACTCTTTTAAGAAGGCTGAATCAAAAGCGGGCTTTAGCCCACTTGTAAGTG
AGTGA

9. Gene sequence of GSAM$_B$

ATGAGAAGCTATGAAAAATCAAAAACGGCTTTTAAAGAAGCGCAAAAACTCATG
CCGGGCGGTGTGAACAGTCCCGTTCGCGCATTTAAATCGGTAGACATGGACCCGA
TTTTTATGGAGCGCGGAAAAGGCTCGAAAATCTTTGATATTGACGGGAATGAATA
TATTGACTACGTCTTGTCATGGGGGCCTTTAATTTTAGGGCATACAAATGACCGC
GTCGTAGAAAGCCTCAAAAAAGTGGCTGAATACGGGACAAGCTTTGGTGCTCCG
ACTGAAGTAGAAAATGAACTGGCTAAGCTCGTCATTGATCGTGTGCCATCTGTAG
AAATTGTACGAATGGTAAGCTCCGGAACAGAGGCTACAATGAGTGCCCTCCGTTT
GGCAAGGGGCTATACGGGCCGCAACAAGATTTTAAAATTTGAGGGCTGCTACCA
CGGACACGGCGATTCTCTCTTGATTAAAGCTGGTTCAGGTGTTGCCACTCTCGGTC
TGCCTGACAGCCCGGGGGTGCCTGAAGGCATTGCGAAAAACACCATCACCGTTCC

GTACAATGATTTAGAAAGTGTAAAGCTTGCTTTCCAGCAATTCGGTGAAGACATT
GCGGGAGTCATTGTAGAGCCAGTTGCCGGAAATATGGGTGTTGTTCCGCCGCAAG
AAGGTTTCCTTCAGGGTCTGCGTGATATCACTGAGCAGTACGGCTCCCTGCTGATT
TTTGATGAAGTGATGACTGGCTTCCGGGTCGATTATAACTGCGCTCAAGGCTACT
TCGGCGTAACGCCTGATCTGACTTGTTTAGGAAAAGTAATCGGGGGCGGACTTCC
TGTCGGCGCTTATGGCGGAAAGGCAGAAATCATGGAGCAGATCGCTCCAAGCGG
TCCGATCTATCAAGCTGGTACATTGTCAGGCAACCCGCTTGCGATGACGGCTGGC
TTAGAGACATTGAAACAGCTGACACCTGAATCCTACAAGAATTTCATCAAAAAAG
GCGACAGACTGGAAGAAGGAATTTCAAAAACCGCCGGGGCTCATGGCATTCCGC
ATACCTTTAACCGTGCAGGTTCGATGATCGGTTTCTTCTTTACAAACGAACCAGTC
ATCAATTATGAAACAGCGAAATCATCTGATTTGAAGCTGTTCGCAAGCTATTATA
AAGGGATGGCAAATGAAGGGGTATTCCTTCCGCCATCACAATTCGAAGGTCTTTT
CCTCTCAACGGCCCATACTGATGAAGATATTGAAAACACAATCCAGGCAGCTGAG
AAAGTATTTGCTGAGATCAGCCGCAGATAA

10. Gene sequence of GBD domain-Linker-SH3 domain-Linker-PDZ domain
ATGACTAAGGCTGATATTGGTACTCCATCTAACTTTCAACATATTGGTCATGTTGG
TTGGGATCCAAACACTGGTTTTGATTTGAACAACTTGGATCCAGAATTGAAGAAC
TTGTTTGATATGTGTGGTATTTCTGAAGCTCAATTGAAGGATAGAGAAACTTCTA
AGGTTATTTACGATTTTATTGAAAAGACTGGTGGTGTTGAAGCTGTTAAGAACGA
ATTGAGAAGACAAGCTCCAGGTTCTGGTTCTGGTTCTGGTTCTGGTGAAGCTGAA
TACGTTAGAGCTTTGTTTGATTTTAACGGTAACGATGAAGAAGATTTGCCATTTAA
GAAGGGTGATATTTTGAGAATTAGAGATAAGCCAGAAGAACAATGGTGGAACGC
TGAAGATTCTGAAGGTAAGAGAGGTATGATTCCAGTTCCATACGTTGAAAAGTAC
GGTTCTGGTTCTGGTTCTGGTTCTGGTTTGCAAAGAAGAAGAGTTACTGTTAGAA
AGGCTGATGCTGGTGGTTTGGGTATTTCTATTAAGGGTGGTAGAGAAAACAAGAT
GCCAATTTTGATTTCTAAGATTTTTAAGGGTTTGGCTGCTGATCAAACTGAAGCTT
TGTTTGTTGGTGATGCTATTTTGTCTGTTAACGGTGAAGATTTGTCTTCTGCTACTC
ATGATGAAGCTGTTCAAGCTTTGAAGAAGACTGGTAAGGAAGTTGTTTTGGAAGT
TAAGTACATGAAGGAAGTTTCTCCATACTTTAAGTAA

11. Gene sequence of GBD ligand
ATGTTGGTTGGTGCTTTGATGCATGTTATGCAAAAGAGATCTAGAGCTATTCATTC
TTCTGATGAAGGTGAAGATCAAGCTGGTGATGAAGATGAAGATTAA

12. Gene sequence of SH3 ligand
ATGCCACCACCAGCTTTGCCACCAAAGAGAAGAAGATAA

13. Gene sequence of PDZ ligand
ATGGGTGTTAAGGAATCTTTGGTTTAA

14. Gene sequence of P_{G7} promoter
TTTTTGTAGAAATGTCTTGGTGTCCTCGTCCAATCAGGTAGCCATCTCTGAAATAT
CTGGCTCCGTTGCAACTCCGAACGACCTGCTGGCAACGTAAAATTCTCCGGGGTA
AAACTTTAATGTGGAGTAATGGAACCAGAAACGTCTCTTCCCTTCTCTCTCCTTCC
ACCGCCCGTTACCGTCCCTAGGAAATTTTACTCTGCGGGAGAGCTTCTCCTACGG
CCCCCTTGCAGCAATGCTCTCCCCAGAATTACGTCGCGGGTAAAACGGAGGTCGT
GTACCCGACCTAGCAGCCCAGGGATGGAAAAGTCCCGGCCGTCGCTGGCAATAA
TAGCGGGCGGGCGCATGTCATGAGACTATTGGAAACCACCAGAATCGAATATAA
AAGGCGAACACCTTTCCCTATTTTGGTTTCTCCTGACCCAAAGACTTTAAAATTAA
TTTATTTGTCCCTATTTCAATCAATTGAACAACTAT

15. pPIC3.5K-*ku70*-gRNA1 map and sequence

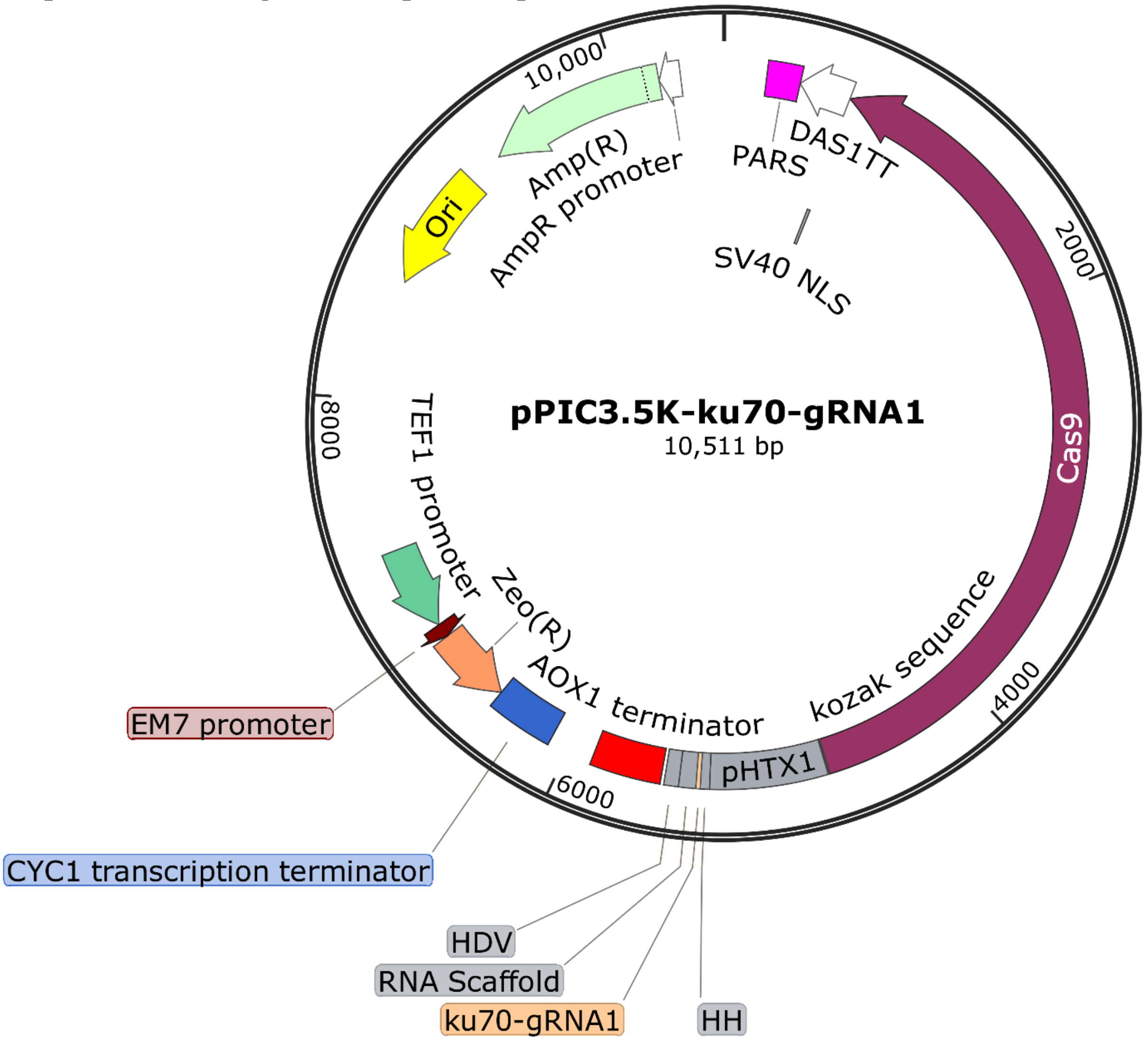

AGATCTAACATCCAAAGACGAAAGGTTGAATGAAACCTTTTTGCCATCCGACATC
CACAGGTCCATTCTCACACATAAGTGCCAAACGCAACAGGAGGGGATACACTAG
CAGCAGACCGTTGCAAACGCAGGACCTCCACTCCTCTTCTCCTCAACACCCACTTT
TGCCATCGAAAAACCAGCCCAGTTATTGGGCTTGATTGGAGCTTCGACAATTAAT
ATTTACTTATTTTGGTCAACCCCAAATAGGTTGATTTCATACTTGGTTCATTCAAA
AATAAGTAGTCTTTTGAGATCTTTCAATATTATAATAAATATACTATAACAGCCG
ACTTGTTTCATTTTCGCGAATGTTCCCCCAGCTTATCTCGAGACCCTTGTGACTGA
CACTTTGGGAGTCCCTATTCTACTTAGTCTCATATCGCATGAAACTTTTGATAAAT
TATTTTCTGATAGGAATTTTTCATCAGATATTATCATCGCGGCTTACGTAATAACA
AAAAAAATTGATGGAGTCTATACTAGGCTAACATAAACTAAGTTATTAATTAAAC
AAAACAAAACGTACTAGCATTACTGTCATATATAAGGGCTCCTAACTAAAACTGT
AAAGACTTCCCGTTCACACCTTCCTCTTCTTCTTGGGGTCAGCCCTGCTGTCTCCA
CCGAGCTGAGAGAGGTCGATTCTTGTTTCATAGAGCCCCGTAATTGACTGATGAA
TCAGTGTGGCGTCCAGGACCTCCTTTGTAGAGGTGTACCGCTTTCTGTCTATGGTG
GTGTCGAAGTACTTGAAGGCTGCAGGCGCGCCCAAGTTGGTCAGAGTAAACAAG
TGGATAATGTTTTCTGCCTGCTCCCTGATGGGCTTATCCCTGTGCTTATTGTAAGC
AGAAAGCACCTTATCGAGGTTAGCGTCGGCGAGGATCACTCTTTTGGAGAATTCG
CTTATTTGCTCGATGATCTCATCAAGGTAGTGTTTGTGTTGTTCCACGAACAGCTG
CTTCTGCTCATTATCTTCGGGAGACCCTTTGAGCTTTTCATAGTGGCTGGCCAGAT

ACAAGAAATTAACGTATTTAGAGGGCAGTGCCAGCTCGTTACCTTTCTGCAGCTC
GCCCGCACTAGCGAGCATTCGTTTCCGGCCGTTTTCAAGCTCAAAGAGAGAGTAC
TTGGGAAGCTTAATGATGAGGTCTTTTTTGACCTCTTTATATCCTTTCGCCTCGAG
AAAGTCGATGGGGTTTTTTTCGAAGCTTGATCGCTCCATGATTGTGATGCCCAGC
AGTTCCTTGACGCTTTTGAGTTTTTTAGACTTCCCTTTCTCCACTTTGGCCACAACC
AGTACACTGTAAGCGACTGTAGGAGAATCGAATCCGCCGTATTTCTTGGGGTCCC
AATCTTTTTTGCGTGCGATCAGCTTGTCGCTGTTCCTTTTCGGGAGGATACTTTCCT
TGGAGAAGCCTCCGGTCTGTACTTCGGTCTTTTTAACGATGTTCACCTGCGGCATG
GACAGGACCTTCCGGACTGTCGCGAAATCCCTACCCTTGTCCCACACGATTTCTCC
TGTTTCTCCGTTTGTTTCGATAAGTGGTCGCTTCCGAATCTCTCCATTGGCCAGTG
TAATCTCGGTCTTGAAAAAATTCATAATATTGCTGTAAAAGAAGTACTTAGCGGT
GGCCTTGCCTATTTCCTGCTCAGACTTTGCGATCATTTTCCTAACATCGTACACTTT
ATAGTCTCCGTAAACAAATTCAGATTCAAGCTTGGGATATTTTTTGATAAGTGCA
GTGCCTACCACTGCATTCAGGTAGGCATCATGCGCATGGTGGTAATTGTTGATCT
CTCTCACCTTATAAAACTGAAAGTCCTTTCTGAAATCTGAGACCAGCTTAGACTTC
AGAGTAATAACTTTCACCTCTCGAATCAGTTTGTCATTTTCATCGTACTTGGTGTT
CATGCGTGAATCGAGAATTTGGGCCACGTGCTTGGTGATCTGGCGTGTCTCAACA
AGCTGCCTTTTGATGAAGCCGGCTTTATCCAACTCAGACAGGCCACCTCGTTCAG
CCTTAGTCAGATTATCGAACTTCCGTTGTGTGATCAGTTTGGCGTTCAGCAGCTGC
CGCCAATAATTTTTCATTTTCTTGACAACTTCTTCTGAGGGGACGTTATCACTCTT
CCCTCTATTTTTATCGGATCTTGTCAACACTTTATTATCAATAGAATCATCTTTGA
GAAAAGACTGGGGCACGATATGATCCACGTCGTAGTCGGAGAGCCGATTGATGT
CCAGTTCCTGATCCACGTACATGTCCCTGCCGTTCTGCAGGTAGTACAGGTAGAG
CTTCTCATTCTGAAGCTGGGTGTTTTCAACTGGGTGTTCCTTAAGGATTTGGGACC
CCAGTTCTTTTATACCCTCTTCAATCCTCTTCATCCTTTCCCTACTGTTCTTCTGTCC
CTTCTGGGTAGTTTGGTTCTCTCGGGCCATCTCGATAACGATATTCTCGGGCTTAT
GCCTTCCCATTACTTTGACGAGTTCATCCACGACCTTAACGGTCTGCAGTATTCCC
TTTTTGATAGCTGGGCTACCTGCAAGATTAGCGATGTGCTCGTGAAGACTGTCCC
CCTGGCCAGAAACTTGTGCTTTCTGGATGTCCTCCTTAAAGGTGAGAGAGTCATC
ATGGATCAACTGCATGAAGTTCCGGTTGGCAAATCCATCGGACTTAAGAAAATCC
AGGATTGTCTTTCCACTCTGCTTGTCTCGGATCCCATTGATCAGTTTTCTTGACAG
CCGCCCCCATCCTGTATATCGGCGCCTCTTGAGCTGTTTCATGACTTTGTCGTCGA
AGAGATGAGCGTAAGTTTTCAAGCGTTCTTCAATCATCTCCCTATCTTCAAACAAC
GTAAGGGTGAGGACAATGTCCTCAAGAATGTCCTCGTTCTCCTCATTGTCCAGGA
AGTCCTTGTCTTTAATGATTTTCAGGAGATCGTGATACGTTCCCAGGGATGCGTTG
AAGCGATCCTCCACTCCGCTGATTTCAACAGAGTCGAAACATTCAATCTTTTTGA
AATAGTCTTCTTTGAGCTGTTTCACGGTAACTTTCCGGTTCGTCTTGAAGAGGAGG
TCCACGATAGCTTTCTTCTGCTCTCCAGACAGGAATGCTGGCTTTCTCATCCCTTC
TGTGACGTATTTGACCTTGGTGAGCTCGTTATAAACTGTGAAGTACTCGTACAGC
AGAGAGTGTTTAGGAAGCACCTTTTCGTTAGGCAGATTTTTATCAAAGTTAGTCA
TCCTTTCGATGAAGGACTGGGCAGAGGCCCCCTTATCCACGACTTCCTCGAAGTT
CCAGGGAGTGATGGTCTCTTCTGATTTGCGAGTCATCCACGCGAATCTGGAATTT
CCCCGGGCGAGGGGGCCTACATAGTAGGGTATCCGAAATGTGAGGATTTTCTCAA
TCTTTTCCCTGTTATCTTTCAAAAAGGGGTAGAAATCCTCTTGCCGCCTGAGGATA
GCGTGCAGTTCGCCCAGGTGAATCTGGTGGGGGATGCTTCCATTGTCGAAAGTGC
GCTGTTTGCGCAACAGATCTTCTCTGTTAAGCTTTACCAGCAGCTCCTCGGTGCCG
TCCATTTTTTCCAAGATGGGCTTAATAAATTTGTAAAATTCCTCCTGGCTTGCTCC
GCCGTCAATGTATCCGGCGTAGCCATTTTTAGACTGATCGAAGAAAATTTCCTTGT
ACTTCTCAGGCAGTTGCTGTCTGACAAGGGCCTTCAGCAAAGTCAAGTCTTGGTG
GTGCTCATCATAGCGCTTGATCATACTAGCGCTCAGCGGAGCTTTGGTGATCTCC
GTGTTCACTCGCAGAATATCACTCAGCAGAATGGCGTCTGACAGGTTCTTTGCCG

CCAAAAAAAGGTCTGCGTACTGGTCGCCGATCTGGGCCAGCAGATTGTCGAGATC
ATCATCGTAGGTGTCTTTGCTCAGTTGAAGCTTGGCATCTTCGGCCAGGTCGAAGT
TAGATTTAAAGTTGGGGGTCAGCCCGAGTGACAGGGCGATAAGATTACCAAACA
GGCCGTTCTTCTTCTCCCCAGGGAGCTGTGCGATGAGGTTTTCGAGCCGCCGGGA
TTTGGACAGCCTAGCGCTCAGGATTGCTTTGGCGTCAACTCCGGATGCGTTGATC
GGGTTCTCTTCGAAAAGCTGATTGTAAGTCTGAACCAGTTGGATAAAGAGTTTGT
CGACATCGCTGTTGTCTGGGTTCAGGTCCCCCTCGATGAGGAAGTGTCCCCGAAA
TTTGATCATATGCGCCAGCGCGAGATAGATCAACCGCAAGTCAGCCTTATCAGTA
CTGTCTACAAGCTTCTTCCTCAGATGATATATGGTTGGGTACTTTTCATGGTACGC
CACCTCGTCCACGATATTGCCAAAGATTGGGTGGCGCTCGTGCTTTTTATCCTCCT
CCACCAAAAAGGACTCCTCCAGCCTATGGAAGAAAGAGTCATCCACCTTAGCCAT
CTCATTACTAAAGATCTCCTGCAGGTAGCAGATCCGATTCTTTCTGCGGGTATATC
TGCGCCGTGCTGTTCTTTTGAGCCGCGTGGCTTCGGCCGTCTCCCCGGAGTCGAAC
AGGAGGGCGCCAATGAGGTTCTTCTTTATGCTGTGGCGATCGGTATTGCCCAGAA
CTTTGAATTTTTTGCTCGGCACCTTGTACTCGTCCGTAATGACGGCCCAACCGACG
CTGTTTGTGCCGATATCGAGCCCAATGGAGTACTTCTTGTCCATCGTTTCGTGTTG
TAGTTTTAATATAGTTTGAGTATGAGATGGAACTCAGAACGAAGGAATTATCACC
AGTTTATATATTCTGAGGAAAGGGTGTGTCCTAAATTGGACAGTCACGATGGCAA
TAAACGCTCAGCCAATCAGAATGCAGGAGCCATAAATTGTTGTATTATTGCTGCA
AGATTTATGTGGGTTCACATTCCACTGAATGGTTTTCACTGTAGAATTGGTGTCCT
AGTTGTTATGTTTCGAGATGTTTTCAAGAAAAACTAAAATGCACAAACTGACCAA
TAATGTGCCGTCGCGCTTGGTACAAACGTCAGGATTGCCACCACTTTTTTCGCACT
CTGGTACAAAAGTTCGCACTTCCCACTCGTATGTAACGAAAAACAGAGCAGTCTA
TCCAGAACGAGACAAATTAGCGCGTACTGTCCCATTCCATAAGGTATCATAGGAA
ACGAGAGTCCTCCCCCCATCACGTATATATAAACACACTGATATCCCACATCCGC
TTGTCACCAAACTAATACATCCAGTTCAAGTTACCTAAACAAATCAAAAAGATGC
TGATGAGTCCGTGAGGACGAAACGAGTAAGCTCGTCCATCTTAGAGAATGTCAGT
GGTTTTAGAGCTAGAAATAGCAAGTTAAAATAAGGCTAGTCCGTTATCAACTTGA
AAAAGTGGCACCGAGTCGGTGCTTTTGGCCGGCATGGTCCCAGCCTCCTCGCTGG
CGCCGGCTGGGCAACATGCTTCGGCATGGCGAATGGGACGGCCGCGAATTAATTC
GCCTTAGACATGACTGTTCCTCAGTTCAAGTTGGGCACTTACGAGAAGACCGGTC
TTGCTAGATTCTAATCAAGAGGATGTCAGAATGCCATTTGCCTGAGAGATGCAGG
CTTCATTTTTGATACTTTTTTATTTGTAACCTATATAGTATAGGATTTTTTTTGTCA
TTTTGTTTCTTCTCGTACGAGCTTGCTCCTGATCAGCCTATCTCGCAGCTGATGAA
TATCTTGTGGTAGGGGTTTGGGAAAATCATTCGAGTTTGATGTTTTTCTTGGTATT
TCCCACTCCTCTTCAGAGTACAGAAGATTAAGTGAGACGTTCGTTTGTGCAAGCT
TATCGATAAGCTTTAATGCGGTAGTTTATCACAGTTAAATTGCTAACGCAGTCAG
GCACCGTGTATGAAATCTAACAATGCGCTCATCGTCATCCTCGGCACCGTCACCC
TGGATGCTGTAGGCATAGGCTTGGTTATGCCGGTACTGCCGGGCCTCTTGCGGGA
TATCGTCCATTCCGACAGCATCGCCAGTCACTATGGCGTGCTGCTAGCAGCTTGC
AAATTAAAGCCTTCGAGCGTCCCAAAACCTTCTCAAGCAAGGTTTTCAGTATAAT
GTTACATGCGTACACGCGTCTGTACAGAAAAAAAAGAAAAATTTGAAATATAAA
TAACGTTCTTAATACTAACATAACTATAAAAAAATAAATAGGGACCTAGACTTCA
GGTTGTCTAACTCCTTCCTTTTCGGTTAGAGCGGATGTGGGGGGAGGGCGTGAAT
GTAAGCGTGACATAACTAATTACATGATATCGACAAAGGAAAAGGGGGACGGAT
CTCCGAGGCCTGGGACCCGTGGGCCGCCGTCGGACGTGTCAGTCCTGCTCCTCGG
CCACGAAGTGCACGCAGTTGCCGGCCGGGTCGCGCAGGGCGAACTCCCGCCCCC
ACGGCTGCTCGCCGATCTCGGTCATGGCCGGCCCGGAGGCGTCCCGGAAGTTCGT
GGACACGACCTCCGACCACTCGGCGTACAGCTCGTCCAGGCCGCGCACCCACACC
CAGGCCAGGGTGTTGTCCGGCACCACCTGGTCCTGGACCGCGCTGATGAACAGGG
TCACGTCGTCCCGGACCACACCGGCGAAGTCGTCCTCCACGAAGTCCCGGGAGAA

CCCGAGCCGGTCGGTCCAGAACTCGACCGCTCCGGCGACGTCGCGCGCGGTGAG
CACCGGAACGGCACTGGTCAACTTGGCCATGGTTTAGTTCCTCACCTTGTCGTATT
ATACTATGCCGATATACTATGCCGATGATTAATTGTCAACACCGCCCCTTAGATTA
GATTGCTATGCTTTCTTTCTAATGAACAAGAAGTAAAAAAAGTTGTAATAGAACA
AGAAAAATGAAACTGAAACTTGAGAAATTGAAGACCGTTTATTAACTTAAATATC
AATGGAGGTCACTGAAAGAGAAAAAAACTAAAAAAAAAAAATTTCAAGAAAAAG
AAACGTGATAAAAATTTTTATTGCCTTTTTCGACGAAGAAAAAGAAACGAGGCGG
TCTCTTTTTTCTTTTCCAAACCTTTAGTACGGGTAATTAACGACACCCTAGAGGAA
GAAAGAGGGAAAATTTAGTATGCTGTGCTTGGGTGTTTTGAAGTGGTACGGCGAT
GCGCGGAGTCCGAGAAAATCTGGAAGAGTAAAAAAGGAGTAGAAACATTTTGAA
GCTATGGTGTGTGGGGCATGCACCATTCCTTGCGGCGGCGGTGCTCAACGGCCTC
AACCTACTACTGGGCTGCTTCCTAATGCAGGAGTCGCATAAGGGAGAGCGTCGAG
TATCTATGATTGGAAGTATGGGAATGGTGATACCCGCATTCTTCAGTGTCTTGAG
GTCTCCTATCAGATTATGCCCAACTAAAGCAACCGGAGGAGGAGATTTCATGGTA
AATTTCTCTGACTTTTGGTCATCAGTAGACTCGAACTGTGAGACTATCTCGGTTAT
GACAGCAGAAATGTCCTTCTTGGAGACAGTAAATGAAGTCCCACCAATAAAGAA
ATCCTTGTTATCAGGAACAAACTTCTTGTTTCGAACTTTTTCGGTGCCTTGAACTA
TAAAATGTAGAGTGGATATGTCGGGTAGGAATGGAGCGGGCAAATGCTTACCTTC
TGGACCTTCAAGAGGTATGTAGGGTTTGTAGATACTGATGCCAACTTCAGTGACA
ACGTTGCTATTTCGTTCAAACCATTCCGAATCCAGAGAAATCAAAGTTGTTTGTCT
ACTATTGATCCAAGCCAGTGCGGTCTTGAAACTGACAATAGTGTGCTCGTGTTTT
GAGGTCATCTTTGTATGAATAAATCTAGTCTTTGATCTAAATAATCTTGACGAGCC
AAGGCGATAAATACCCAAATCTAAAACTCTTTTAAAACGTTAAAAGGACAAGTAT
GTCTGCCTGTATTAAACCCCAAATCAGCTCGTAGTCTGATCCTCATCAACTTGAGG
GGCACTATCTTGTTTTAGAGAAATTTGCGGAGATGCGATATCGAGAAAAAGGTAC
GCTGATTTTAAACGTGAAATTTATCTCAAGATCTCTGCCTCGCGCGTTTCGGTGAT
GACGGTGAAAACCTCTGACACATGCAGCTCCCGGAGACGGTCACAGCTTGTCTGT
AAGCGGATGCCGGGAGCAGACAAGCCCGTCAGGGCGCGTCAGCGGGTGTTGGCG
GGTGTCGGGGCGCAGCCATGACCCAGTCACGTAGCGATAGCGGAGTGTATACTG
GCTTAACTATGCGGCATCAGAGCAGATTGTACTGAGAGTGCACCATATGCGGTGT
GAAATACCGCACAGATGCGTAAGGAGAAAATACCGCATCAGGCGCTCTTCCGCTT
CCTCGCTCACTGACTCGCTGCGCTCGGTCGTTCGGCTGCGGCGAGCGGTATCAGC
TCACTCAAAGGCGGTAATACGGTTATCCACAGAATCAGGGGATAACGCAGGAAA
GAACATGTGAGCAAAAGGCCAGCAAAAGGCCAGGAACCGTAAAAAGGCCGCGTT
GCTGGCGTTTTTCCATAGGCTCCGCCCCCCTGACGAGCATCACAAAAATCGACGC
TCAAGTCAGAGGTGGCGAAACCCGACAGGACTATAAAGATACCAGGCGTTTCCC
CCTGGAAGCTCCCTCGTGCGCTCTCCTGTTCCGACCCTGCCGCTTACCGGATACCT
GTCCGCCTTTCTCCCTTCGGGAAGCGTGGCGCTTTCTCAATGCTCACGCTGTAGGT
ATCTCAGTTCGGTGTAGGTCGTTCGCTCCAAGCTGGGCTGTGTGCACGAACCCCC
CGTTCAGCCCGACCGCTGCGCCTTATCCGGTAACTATCGTCTTGAGTCCAACCCG
GTAAGACACGACTTATCGCCACTGGCAGCAGCCACTGGTAACAGGATTAGCAGA
GCGAGGTATGTAGGCGGTGCTACAGAGTTCTTGAAGTGGTGGCCTAACTACGGCT
ACACTAGAAGGACAGTATTTGGTATCTGCGCTCTGCTGAAGCCAGTTACCTTCGG
AAAAAGAGTTGGTAGCTCTTGATCCGGCAAACAAACCACCGCTGGTAGCGGTGG
TTTTTTTGTTTGCAAGCAGCAGATTACGCGCAGAAAAAAAGGATCTCAAGAAGAT
CCTTTGATCTTTTCTACGGGGTCTGACGCTCAGTGGAACGAAAACTCACGTTAAG
GGATTTTGGTCATGAGATTATCAAAAAGGATCTTCACCTAGATCCTTTTAAATTAA
AAATGAAGTTTTAAATCAATCTAAAGTATATATGAGTAAACTTGGTCTGACAGTT
ACCAATGCTTAATCAGTGAGGCACCTATCTCAGCGATCTGTCTATTTCGTTCATCC
ATAGTTGCCTGACTCCCCGTCGTGTAGATAACTACGATACGGGAGGGCTTACCAT
CTGGCCCCAGTGCTGCAATGATACCGCGAGACCCACGCTCACCGGCTCCAGATTT

ATCAGCAATAAACCAGCCAGCCGGAAGGGCCGAGCGCAGAAGTGGTCCTGCAAC
TTTATCCGCCTCCATCCAGTCTATTAATTGTTGCCGGGAAGCTAGAGTAAGTAGTT
CGCCAGTTAATAGTTTGCGCAACGTTGTTGCCATTGCTGCAGGCATCGTGGTGTC
ACGCTCGTCGTTTGGTATGGCTTCATTCAGCTCCGGTTCCCAACGATCAAGGCGA
GTTACATGATCCCCCATGTTGTGCAAAAAAGCGGTTAGCTCCTTCGGTCCTCCGAT
CGTTGTCAGAAGTAAGTTGGCCGCAGTGTTATCACTCATGGTTATGGCAGCACTG
CATAATTCTCTTACTGTCATGCCATCCGTAAGATGCTTTTCTGTGACTGGTGAGTA
CTCAACCAAGTCATTCTGAGAATAGTGTATGCGGCGACCGAGTTGCTCTTGCCCG
GCGTCAACACGGGATAATACCGCGCCACATAGCAGAACTTTAAAAGTGCTCATCA
TTGGAAAACGTTCTTCGGGGCGAAAACTCTCAAGGATCTTACCGCTGTTGAGATC
CAGTTCGATGTAACCCACTCGTGCACCCAACTGATCTTCAGCATCTTTTACTTTCA
CCAGCGTTTCTGGGTGAGCAAAAACAGGAAGGCAAAATGCCGCAAAAAAGGGAA
TAAGGGCGACACGGAAATGTTGAATACTCATACTCTTCCTTTTTCAATATTATTGA
AGCATTTATCAGGGTTATTGTCTCATGAGCGGATACATATTTGAATGTATTTAGAA
AAATAAACAAATAGGGGTTCCGCGCACATTTCCCCGAAAAGTGCCACCTGACGTC
TAAGAAACCATTATTATCATGACATTAACCTATAAAAATAGGCGTATCACGAGGC
CCTTTCGTCTTCAAGAATTAATTCTCATGTTTGACAGCTTATCATCGATAAGCTGA
CTCATGTTGGTATTGTGAAATAGACGCAGATCGGGAACACTGAAAAATAACAGTT
ATTATTCG

Supplementary References

[1] Q. Liu, X. Shi, L. Song, H. Liu, X. Zhou, Q. Wang, Y. Zhang, M. Cai, *Microb. Cell Fact.* **2019**, *18*, 144.

[2] B. Hu, H. Yu, J. Zhou, J. Li, J. Chen, G. Du, S. Y. Lee, X. Zhao, *Adv. Sci.* **2023**, *10*, e2205580.

[3] J. E. Dueber, G. C. Wu, G. R. Malmirchegini, T. S. Moon, C. J. Petzold, A. V. Ullal, K. L. Prather, J. D. Keasling, *Nat. Biotechnol.* **2009**, *27*, 753.

[4] X. Qin, J. Qian, G. Yao, Y. Zhuang, S. Zhang, J. Chu, *Appl. Environ. Microbiol.* **2011**, *77*, 3600.
